# Comprehensive Methylome Characterization of *Mycoplasma genitalium* and *Mycoplasma pneumoniae* at Single-Base Resolution

**DOI:** 10.1371/journal.pgen.1003191

**Published:** 2013-01-03

**Authors:** Maria Lluch-Senar, Khai Luong, Verónica Lloréns-Rico, Javier Delgado, Gang Fang, Kristi Spittle, Tyson A. Clark, Eric Schadt, Stephen W. Turner, Jonas Korlach, Luis Serrano

**Affiliations:** 1 EMBL/CRG Systems Biology Research Unit, Centre for Genomic Regulation (CRG), Barcelona, Spain; 2 Universitat Pompeu Fabra (UPF), Barcelona, Spain; 3 Pacific Biosciences, Menlo Park, California, United States of America; 4 Department of Genetics and Genomic Sciences, Mount Sinai School of Medicine, New York, New York, United States of America; 5 Institució Catalana de Recerca i Estudis Avançats (ICREA), Barcelona, Spain; Progentech, United States of America

## Abstract

In the bacterial world, methylation is most commonly associated with restriction-modification systems that provide a defense mechanism against invading foreign genomes. In addition, it is known that methylation plays functionally important roles, including timing of DNA replication, chromosome partitioning, DNA repair, and regulation of gene expression. However, full DNA methylome analyses are scarce due to a lack of a simple methodology for rapid and sensitive detection of common epigenetic marks (ie N^6^-methyladenine (6 mA) and N^4^-methylcytosine (4 mC)), in these organisms. Here, we use Single-Molecule Real-Time (SMRT) sequencing to determine the methylomes of two related human pathogen species, *Mycoplasma genitalium* G-37 and *Mycoplasma pneumoniae* M129, with single-base resolution. Our analysis identified two new methylation motifs not previously described in bacteria: a widespread 6 mA methylation motif common to both bacteria (5′-CT*A*T-3′), as well as a more complex Type I m6A sequence motif in *M. pneumoniae* (5′-G*A*N_7_TAY-3′/3′-CTN_7_ *A*TR-5′). We identify the methyltransferase responsible for the common motif and suggest the one involved in *M. pneumoniae* only. Analysis of the distribution of methylation sites across the genome of *M. pneumoniae* suggests a potential role for methylation in regulating the cell cycle, as well as in regulation of gene expression. To our knowledge, this is one of the first direct methylome profiling studies with single-base resolution from a bacterial organism.

## Author Summary

DNA methylation in bacteria plays important roles in cell division, DNA repair, regulation of gene expression, and pathogenesis. Here, we use a novel sequencing technique, Single-Molecule Real-Time (SMRT) sequencing, to determine the methylomes of two related human pathogen species, *Mycoplasma genitalium* G-37 and *Mycoplasma pneumoniae* M129. Our analysis identified two novel methylation motifs, one of them present uniquely in *M. pneumoniae* and the other common to both bacteria. We also identify the methyltransferase responsible for the common methylation motif and suggest the one associated with the *M. pneumoniae* unique motif. Functional analysis of the data suggests a potential role for methylation in regulating the cell cycle of *M. pneumoniae*, as well as in regulation of gene expression. To our knowledge, this is one of the first genome-wide approaches to study the biological role of methylation in a bacterial organism.

## Introduction

Among a few documented mechanisms, methylation of specific DNA sequences by DNA methyltransferases provides one way by which epigenetic inheritance can be orchestrated [1]. For instance, in many eukaryotes, methylated cytosine residues at 5′-CG-3′ (CpG) sequences are recognized by methyl-CpG binding proteins that usually repress the transcription of local DNA regions [2]–[5]. In the bacterial world, methylation is most commonly associated with restriction-modification (R-M) systems that provide a defense mechanism against invading foreign genomes [6]. In addition, it is known that a variety of enzymes capable of methylating DNA at adenine [7] and cytosine [8], [9] play functionally important roles, including timing of DNA replication, chromosome partitioning, DNA repair, transposition and conjugal transfer of plasmids, and regulation of gene expression [7], [10]–[16]. Phenomena involving inheritance of DNA methylation patterns are also known in bacteria. These systems use DNA methylation patterns to pass on information regarding the phenotypic expression state of the mother cell to the daughter cells. Methylation can alter the DNA structure and affect the binding of regulatory protein(s) to its DNA target site, thereby controlling gene expression [17], [18]. Notably, most adhesion genes in *Escherichia coli* are regulated by DNA methylation patterns [19], [20]. Little is known about how widespread heritable epigenetic control is in the bacterial world or the roles that epigenetic regulatory systems play in bacterial biology, including pathogenesis. For instance, it has been shown that DNA methylation in *Streptococcus mutans* up-regulates the expression of virulence factors like gbpC and bacteriocins [21]. It has also been shown that in *E. coli*, the expression of the Type IV secretion gene cluster is regulated by a non-stochastic epigenetic switch that depends on methylation of the Fur binding box [22].

In some gram-positive and gram-negative species that have been studied, adenine methylation plays a critical role in regulating chromosome replication. Adenine is generally methylated by members of the Dam family of methyltransferases, such as Dam in *E. coli* and *Dpn*II in *Streptococcus pneumoniae*, that recognize the sequence motif 5′-GATC-3′ [23]. In these bacteria, the protein SeqA binds to hemi-methylated DNA target sites (5′-GATC-3′) clustered at the origin of replication (*oriC*) and sequesters the origin from replication initiation. SeqA also binds to hemi-methylated 5′-GATC-3′ sites in the *dnaA* promoter, blocking the synthesis of DnaA protein, which is necessary for replication initiation [24]–[27]. All of these events use the hemi-methylated state of newly replicated DNA as a signal. This hemi-methylated DNA is generated by semi-conservative replication of a fully methylated DNA molecule. Because of the transient nature of the hemi-methylation state, none of these phenomena are heritable. However, this mechanism is not universal, and other bacteria, like *Bacillus subtilis*, lack the Dam methyltransferase and SeqA proteins that *E. coli* employs to repress (sequester) its *oriC* during replication [28].

While there are many studies demonstrating the potential roles of methylation in epigenetic control of bacteria, the number of studies is significantly smaller than those for eukaryotes. This dearth of studies on bacterial epigenetics is partly due to a lack of a simple methodology that would allow rapid and sensitive detection of common epigenetic markers, such as N^6^-methyladenine (6 mA) and N^4^-methylcytosine (4 mC), in these organisms. Through bisulfite treatment, 5-methylcytosine (5 mC) was the only base modification detectable with efficiency and sensitivity suitable for genome wide epigenetic studies [29], [30]. Recently, Single-Molecule, Real-Time (SMRT) sequencing was described to provide the capability of directly detecting different base modifications beyond the canonical A, C, G, and T bases, in addition to yielding the sequence information [31]. The technique has been successfully demonstrated to identify methyltransferase specificities on plasmids [32].

Here, we use SMRT sequencing to comprehensively determine the methylomes of two mycoplasma species, *Mycoplasma genitalium* and *Mycoplasma pneumoniae*, with single-base and -strand resolution. *M. pneumoniae* and *M. genitalium* are closely related human pathogens that cause atypical pneumonia and non-gonococcal urethritis, respectively [33], [34]. These bacteria are members of the Mollicutes class characterized by the lack of a cell wall and by their reduced genomes with a low GC content. The genome sizes of *M. pneumoniae* and *M. genitalium* are 816 kb and 580 kb, respectively [35], [36]. *M. genitalium* is widely considered to have the smallest genome of any bacteria that can be grown in a test tube in the absence of host cells [37]. Our analysis identified a widespread 6 mA methylation sequence motif common to both bacteria (5′-CT*A*T-3′, with m6A in italics), as well as a more complex Type I m6A sequence motif in *M. pneumoniae* (5′-G*A*N_7_TAY-3′/3′-CTN_7_ *A*TR-5′). Analysis of the chromosome distribution pattern of the first motif in *M. pneumoniae* suggests that methylation is involved in regulating cell division. To our knowledge, this work is one of the first comprehensive methylome analysis of bacteria.

## Results

### Putative restriction modification systems in *M. pneumoniae* and *M. genitalium*

We analyzed the genomes of *M. pneumoniae* and *M. genitalium* for all the putative methyltransferase genes using comparative sequence analysis and our previous functional assignment [38]. In the *M. pneumoniae* genome, we identified different putative Type I and Type II restriction modification systems. Type I involves a complex consisting of three polypeptides: R (restriction), M (modification), and S (specificity). The resulting complex can both cleave and methylate DNA. The S subunit determines the specificity of both restriction and methylation [39]. *M. pneumoniae* Type I system includes a methyltransferase (*mpn342*), a DNA specific recognition protein that brings the methyltransferase to the target DNA (HdsS, *mpn343*), and a restriction enzyme that cleaves unmethylated DNA (HdsR, *mpn345*). The restriction protein HdsR gene contains three frameshift mutations which likely make it inactive (additional protein fragments could be coded by *mpn346* and *mpn347*). There are also some isolated genes encoding duplicated copies of the specificity determining subunit HdsS (*mpn089*, *mpn289, mpn290*, *mpn365*, *mpn507*, *mpn615*, and *mpn638*). In the Type II, methyltransferase and endonuclease are typically encoded as two separate proteins and act independently [39]. In *M. pneumoniae*, Type II systems could consist of the methyltransferase protein (HsdM, *mpn107*, *mpn108* or *mpn111*) and the restriction enzyme (HsdR, *mpn109* or *mpn110*). Additionally, a putative uncharacterized methyltransferase (mte1; *mpn198*), annotated as an *Eco*RI-like methylase in Uniprot and not associated with any R-M system, was identified. *Eco*RI restriction/modification system (R/M) is a Type II system that has been well characterized *in vivo* and *in vitro* [40], [41]. *M. genitalium* has an orthologous of *mpn198* (*mg184*) and only one of the Type II-specificity determining subunits HdsS, *mpn638 (mg438)* (Table 1).

**Table 1:** Levels of RNA and protein for different proteins of M-R systems of *M. pneumoniae*.

Operon	ORF	RNA Micro arrays 6 h	RNA Micro arrays 96 h	Tiling (96 h)	Protein 6 h	Protein 96 h	Protein name	δEssentiality
		Log2	Log2	Log2	Copy Number per cell	Copy Number per cell		
132	MPN342*	12.0	11.2	8.0	20	9	M.MpnII	NE
133	MPN343	9.6	9.3	8.4	3	-	S.MpnII	NE
136	MPN345	8.8	7.8	6.6	-	-	HdsR	NE
136	MPN346	7.9	9.1	6.9	-	-	HdsR	NE
137	MPN347	8.0	8.4	6.1	-	-	HdsR	NE
	MPN089	11.2	11.3	7.8	-	-	HdsS	NE
118	MPN289	5.5	11.9	7.0	-	-	HdsS	NE
118	MPN290	9.7	10.2	6.8	-	-	HdsS	NE
146	MPN365*	0.0	12.1	8.1	3	-	HdsS	NE
216	MPN507*	9.9	9.3	6.3	14	2	HdsS	NE
333	MPN615*	9.0	9.2	6.9	3	1	HdsS	NE
256	MPN638*	10.2	11.8	9.8	322	277	HdsS	NE
49	MPN107	9.6	10.3	7.4	-	-	HsdM	NE
49	MPN108	9.3	8.4	6.9	-	-	HsdM	NE
50	MPN109	9.0	10.3	7.3	42	41	HdsR	NE
50	MPN110	7.0	7.5	6.5	-	-	HdsR	NE
51	MPN111	7.7	7.3	6.6	-	-	HsdM	NE
80	MPN198*	8.7	6.9	7.2	5	2	M.MpnI	E

Data for microarrays and tiling was taken from Guell et al [43]. Data regarding protein copy number was taken from Maier et al. [44] and from unpublished MS analysis done by M. Lluch-Senar. ORFs labeled with an asterisk indicate proteins found to bind to DNA by doing affinity chromatography with a DNA column followed by salt elution and MS (manuscript in preparation). HsdM (Methyltransferase), HdsS (DNA specificity recognition protein), HdsR (Restriction enzyme), M.MpnI (name assigned to the methyltransferase that recognizes 5′-CT*A*T-3′), M.MpnII (name assigned to the putative methyltransferase of the Type I motif) and S.MpnII (HsdS subunit associated to Type I restriction modification system).

δ Essential genome of *M. pneumoniae* has been determined by using a library of minitransposon mutants (manuscript in preparation Lluch-Senar, M. et al.). NE, non essential gene; E, essential gene.

We looked at the transcript and protein levels for the putative genes involved in methylation systems by using information of the transcriptome [42], [43] and proteome [44] of *M. pneumoniae* (Table 1). Although we could detect transcripts in the tiling array for all genes, albeit at very low level for many of them, we could identify in multiple MS experiments unique peptides for only six of them: *mpn109, mpn198*, *mpn342*, *mpn343, mpn615*, and *mpn638* (Table 1). Of these, *mpn198, mpn342, mpn615* and *mpn638* were found to bind DNA by doing affinity chromatography with a DNA column followed by salt elution and MS analysis (manuscript in preparation). Only *mpn198* (mte1; *Eco*RI-like) and *mpn342* (Type I) are putative DNA adenine methyltransferases.

### Methylome characterization of *M. pneumoniae* and *M. genitalium* by SMRT sequencing

**Figure 1:**
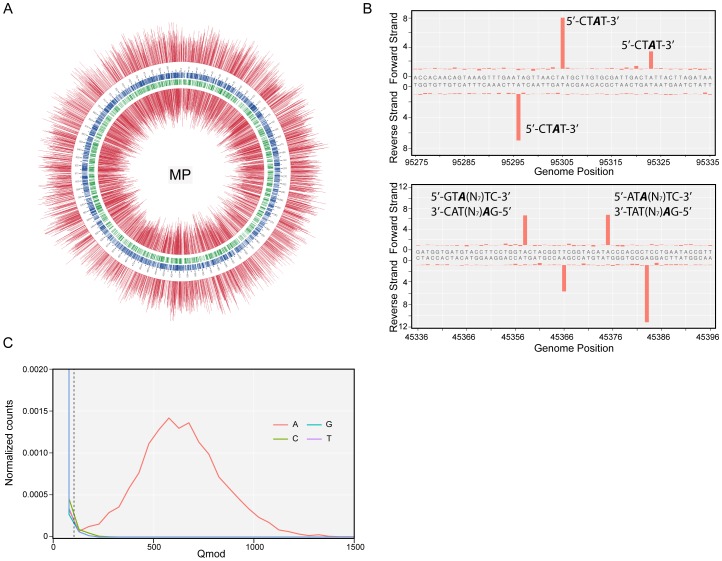
Methylome determination of *M. pneumoniae* by SMRT sequencing. (*A*) Circos plot of kinetic variation across the genome. Red tracks represent the Qmod (−10log(*P*value) from t-test) values of the forward (outer track) and reverse (inner track) strands. Blue and green tracks represent the location of the 5′-CT*A*T-3′ and Type I motifs discovered by filtering on Qmod values as shown in (*C*). (*B*) Example of IPD ratio plots of the two discovered motifs for a section of the genome. The top plot shows 3 instances of 5′-CT*A*T-3′, two of them are asymmetrically methylated. The bottom plot shows 2 Type I examples, where each one is fully methylated; that is each Type I recognition site is methylated on both strands. (*C*) Qmod distribution showing the filtering threshold of 100 used (black dash line) for determining modified positions.

Identification of methylated bases in *M. pneumoniae* and *M. genitalium* genomes was performed by SMRT sequencing at exponential (6 h) and stationary phases (96 h). Figure 1A shows the results of the genome-wide base modification detection analysis for the *M. pneumoniae* genome in stationary phase. The inner and outer most tracks in the Circos plot are the modification values (Qmod) of polymerase kinetics for the reverse and forward strands of the genome relative to an unmodified WGA (whole genome amplification) control. Qmod is the −10log(*P*value) from a t-test and described in further details in the Materials and Methods section. The plot shows many significant peaks which correspond to methylated template positions. Figure 1B shows examples of the IPD (interpulse duration) ratios of a representative genomic section, highlighting both the base and strand resolutions of the technique. The statistically significant peaks, which were defined as Qmod >100 (Figure 1C; see Methods), were clustered as a function of sequence context to determine the recognition motifs of the methyltransferases responsible for the observed signals. The clustering results for *M. pneumoniae* identified >99.9% of all detected genomic positions as falling into two distinct sequence motifs: 5′-CT*A*T-3′ and 5′-G*A*N_7_TAY-3′/3′-CTN_7_ *A*TR-5′ (Y = T or C and R = A or G, with m6A in italics). The first motif is found in both bacteria and is methylated on only one of the two DNA strands. In the second motif, the first adenines in the plus and minus strands are methylated (Figure 1B). The stretch of degenerate bases that separates the two recognition elements in the motif is characteristic of Type I methyltransferase signatures (Figure 1C) [45]. Despite the fact that the second sequence motif appears 1825 times per strand in *M. genitalium* (Table 2), there was no instance where it was detected as methylated. In contrast, this motif appears 1681 times in the genome of *M. pneumoniae* and 1678 are methylated (99,8%, Table 2). Approximately 1–2% of the assigned peaks were secondary peaks of the primary detected m6A and treated as redundant information for the tabulation in Table 2 [31].

**Table 2:** Summary of discovered methylation motifs in *M. genitalium* and *M.pneumoniae*.

Motif	*M. genitalium* number of detected methylations	*M. genitalium* % detected methylations	*M. pneumoniae* number detected methylations	*M. pneumoniae* % detected methylations
5′-CT*A*T-3′	4568	99.6	3306	99.8
5′-G*A*N_7_TAY-3′	-	-	1678	99.8
3′-CTN_7_ *A*TR-5′	-	-	1676	99.7

% detected methylations indicates the percentage of detected methylated sites as compared to all occurrences of the sequence motif in the genome.

Analysis of two biological replicates of *M. pneumoniae* grown for 96 hours showed a reproducibility of 99.88% in the assignment of methylated positions.

### Validation of methylation motifs and assignment to specific methyltransferase genes

Putative Type II independent methyltransferases (HsdM) (*mpn198, mpn107*, and *mpn108*) without an associated DNA recognition partner (HsdS), considered as possible candidates for the methylation of 5′-CT*A*T-3′ motif, were cloned into pRSS vector and then transformed into a methyltransferase-free *E. coli* ER2796 (DB24) [46] (Table S9b) following procedures described previously [32]. *Mpn111* was discarded because it is a duplication of *mpn108*.

After cloning, the different plasmids were isolated and analyzed by SMRT sequencing. Of the three putative single proteins with methyltransferase activity, only *mpn198* was capable of modifying the 5′-CT*A*T-3′ sequence. Interestingly, this is the only one of this group of methyltransferases that was found to be expressed by mass spectroscopy (MS) analyses (Table 1). As expected, no methyltransferase was identified by this approach for the Type I 5′-G*A*N_7_TAY-3′/3′- CTN_7_ *A*TR-5′motif, since Type I motifs also require the DNA recognition protein HsdS [45]. These results agree with the finding that both mycoplasma species are methylated at the same motif (5′-CT*A*T-3′) and share a common methyltransferase, namely, *mpn198* in *M. pneumoniae* and *mg184* in *M. genitalium*.

The fact that our MS analysis in *M. pneumoniae* detected protein expression only for DNA methylases MPN198 and MPN343, together with the lack of a *mpn343* ortologue and the absence of the 5′-G*A*N_7_TAY-3′/3′-CTN_7_ *A*TR-5′ methylated motif in *M. genitalium*, suggest that MPN343 could be responsible for the methylation of the 5′-G*A*N_7_TAY-3′/3′-CTN_7_ *A*TR-5′ motif. These results validated the motifs observed for *M. genitalium* and *M. pneumoniae* and identified them as the recognition sequences of previously unassigned methyltransferases.

The new identified methyltransferases have been submitted in the REBASE and re-named using the standard nomenclature (*mpn198*: M.MpnI, *mpn342*: M.MpnII, *mpn343*: S.MpnII, *mg184*: M.MgeI). M indicates methyltransferase; S refers to the specificity subunit for Type I system; Mpn indicates *M. pneumoniae* and Mge indicates *M. genitalium*.

### Genome-wide methylome analysis

We next focused on *M. pneumoniae* to study the role of methylation in regulating gene expression and DNA replication, since the transcriptome and proteome data are currently available for it [42], [43].

**Figure 2:**
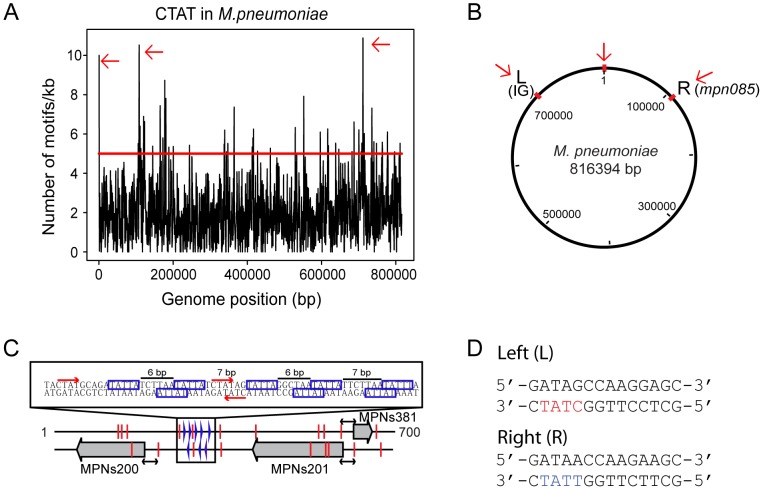
Genome-wide distribution of 5′-CT ***A*** **T-3′** **motif.** (*A*) Hot spots of methylation for the 5′-CT*A*T-3′ motif. The graph represents the number of motifs per 1 kb window. The red line indicates the threshold (5). Regions with more than five 5′-CT*A*T-3′ motifs are considered “hot spots of methylation”. Red arrows indicate the most enriched regions. (*B*) Circular representation of the *M. pneumoniae* genome. Red marks indicate genome locations of the three main enriched regions of methylation. (*C*) Methylation in the putative origin of replication of *M. pneumoniae*. Blue boxes indicate putative DnaA boxes. Red arrows and lines indicate methylation sites. Black arrows indicated a common distance (24 bp) from methylation sites to the TSSs of the three MPNs. (*D*) Motif sequences of two putative cell division “check points” (L, left and R, right). Noteworthy only in L motif contains the recognition motif 5′-CT*A*T-3′ (showed in red letters) on the complementary strand. In contrast, the R motif contains 5′-TTAT-3′ (showed in blue letters) on the complementary strand instead. The three large grey arrows are the three non-coding RNAs (MPNs200, MPNs201 and MPNs381).

To study the putative role of methylation in DNA replication, we analyzed the density distribution of the 5′-CT*A*T-3′ methylation motifs in a sliding window of 1 kb along the *M. pneumoniae* genome (Figure 2A). The mean number of 5′-CT*A*T-3′motifs per 1 kb window is two (±1.6 standard deviation). Regions with more than five 5′-CT*A*T-3′ motifs (*P*value<0.01) were considered to be “hot spots of methylation” for 5′-CT*A*T-3′ (Table S2b). A functional enrichment analysis of all the genes in *M. pneumoniae* present at the 5′-CT*A*T-3′ hotspots showed two functional categories of clusters of orthologous groups (COGs) over-represented: defense mechanisms (*P*value = 0.025) and genes coding for membrane proteins or lipoproteins (*P*value = 9×10^−4^) (Table S4a). Of the hot spots, there are three regions that have more than 10 motifs/kb. Interestingly, these regions are symmetrically distributed around the first kb of the genome (Figure 2B). This region of the genome comprises an intergenic region of 687 bp with three non-coding RNAs (MPNs200, MPNs201, and MPNs381) that frame eight repetitive 5′-TATTA-3′ sequences (identified as DnaA boxes based on Chip-seq analysis; Yus et al manuscript in preparation; Figure 2C [47]). There are three 5′-CT*A*T-3′ methylation motifs, two of them in overlapping and opposite strands of the region with the putative DnaA boxes suggesting that DNA methylation, although different from *E. coli*, could play a role in DNA replication. The other two regions are located at approximately 105 kb to the left and right from the putative origin of replication (Figure 2B). Search of common motifs in these two methylation hot spots revealed a common motif of 14 bp (5′-GATAG/ACCAAGG/AAGC-3′) (Figure 2D). This motif is found at opposite strands in the two regions, but only the left side region contains the 5′-CT*A*T-3′ sequence overlapping.

**Figure 3:**
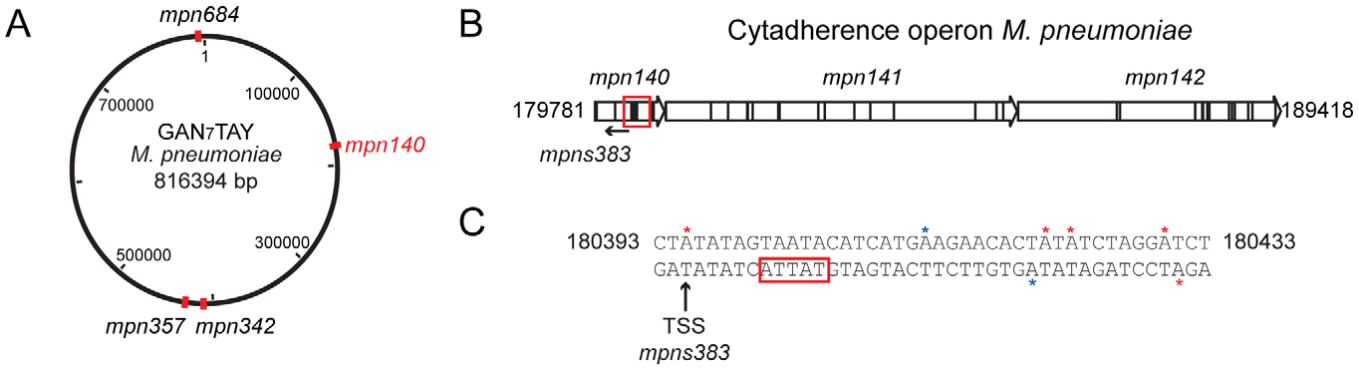
Genome-wide distribution of 5′-G*A*N_7_TAY-3′/3′-CTN_7_ *A*TR-5′ motif. (*A*) Map of the genome of *M. pneumoniae* representing the enriched regions for the 5′-G*A*N_7_TAY-3′/3′-CTN_7_ *A*TR-5′ motif. (*B*) Schematic representation of the cytadherence operon. The red square indicates the main “hot spot” of methylation for the Type I motif. (*C*) Upstream sequence of MPNs383. Red stars indicate the methylation by M.MpnI and blue stars indicate methylation by M.MpnII. The black arrow shows the transcriptional start site (TSS) of the MPNs383 and the red box the promoter sequence of this antisense RNA.

We also analyzed the genome-wide distribution of the Type I motif. The average distribution for the 5′-G*A*N_7_TAY-3′/3′- CTN_7_ *A*TR-5′motif in 1 kb is 1 motif/kb (±1.1 standard deviation), and hot spot regions were considered to be those with more than 3 motifs within 1 kb (*P*value<0.01). Most of the genes that overlap with these hotspots are of unknown function with a *P*value of 0.04 (Table S4b). There are four 1 kb regions in the genome that have more than five instances of 5′-G*A*N_7_TAY-3′/3′-CTN_7_ *A*TR-5′methylation (Figure 3A, and Table S2a). Interestingly, this highly methylated region with the most motifs (6 in 582 bp), is within *mpn140*, the first gene of the cytadherence operon that contains one of the main virulence factors of *M. pneumoniae* (Figure 3B). These motifs are located just upstream of the transcriptional start site (TSS) of an antisense transcript (MPNs383) that could be involved in regulating the expression of *mpn140* (Figure 3C). The other three enriched regions correspond to *mpn684* (that encodes a conserved hypothetical protein), *mpn357* (DNA ligase), and *mpn358* (conserved hypothetical protein) and, surprisingly, to the region containing *mpn342* (M.MpnII) and *mpn343* (S.MpnII). As mentioned above, M.MpnII is the putative methyltransferase responsible for 5′-G*A*N_7_TAY-3′/3′-CTN_7_ *A*TR-5′ methylation.

### Analysis of unmethylated motifs

**Figure 4:**
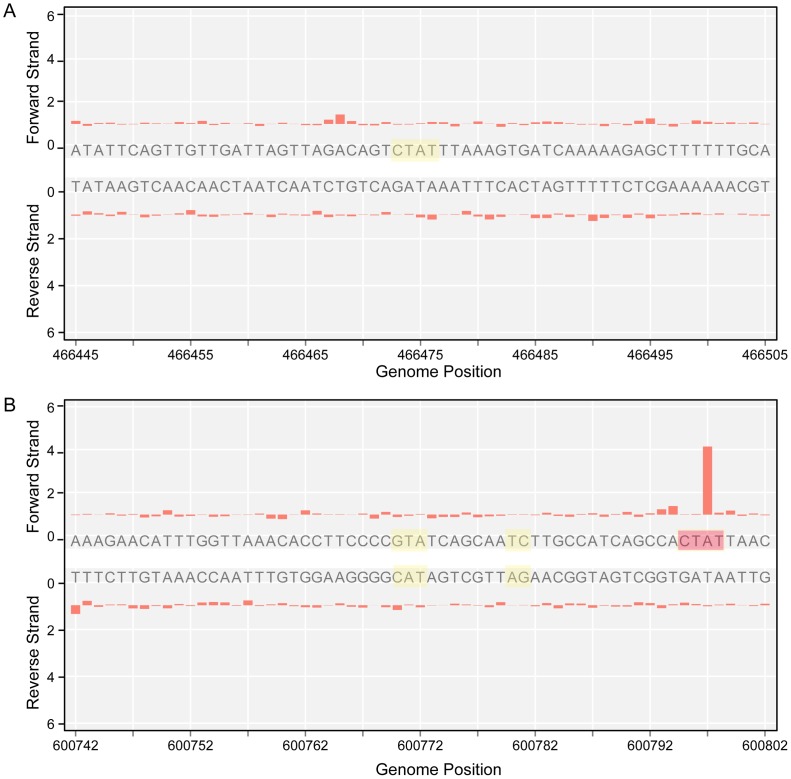
Unmethylated sites. A) IPD ratio plot of a 5′-CT*A*T-3′ site not detected as methylated (shadowed in yellow). B) IPD ratio plot of an unmethylated 5′-G*A*N_7_TAY-3′/3′-CTN_7_ *A*TR-5′ motif (shadowed in yellow). For comparison of signal intensity, a methylated 5′-CT*A*T-3′ is also shown in the bottom plot (shadowed in red).

The genome-wide access to methylation information allows for the interrogation of genomic locations which match the methyltransferases sequence targets, but are kept in an unmethylated state by the bacterium. The results in Table 3 show 5′-CT*A*T-3′ and 5′-G*A*N_7_TAY-3′/3′-CTN_7_ *A*TR-5′ sites that are always unmethylated, two examples are shown in Figure 4. Only one unmethylated 5′-CT*A*T-3′ site was identified (genome position: 466475). This motif is overlapping with the stop codon of the *mpn390* gene that codifies for the dihydrolipoamide dehydrogenase (PdhD). This gene together with *mpn391* (PdhC, dihydrolipoamide acetyltransferase) constitute an operon involved in pyruvate metabolism. Also, three 5′-G*A*N_7_TAY-3′/3′-CTN_7_ *A*TR-5′ unmethylated sites were detected. One is located in an intergenic region and the other two sites are located inside *mpn493* (UlaD, 3-keto-L-gulonate-6-phosphate decarboxylase) involved in ascorbate and aldarate metabolism and *mpn503* (cytadherence protein) (Table 3). We hypothesize that these unmethylated sites indicate the presence of an interacting protein or a DNA structure that is protecting from methylation along the different phases of growth.

**Table 3:** Instances of genomic positions in *M. pneumoniae* consistently detected as lacking methylation across all three samples sequenced.

Motif	Reference Position	Genome Location
5′-AT*A*N_7_TC-3′ 3′-TATN_7_ *A*G-5′	335301335309	MPN282-MPN283
5′-GT*A*N_7_TC-3′ 3′-CATN_7_ *A*G-5′	600772600780	MPN493
5′-G*A*N_7_TAC-3′ 3′-CTN_7_ *A*TG-5′	612097612105	MPN503
5′-CT*A*T-3′	466475	MPN390

### Functional correlations of the *M. pneumoniae* methylome

Recent identification of TSSs in *M. pneumoniae* [42] allowed us to study methylation patterns in promoter regions. We analyzed the regions comprising 40 bp upstream from the TSS (e.g. the promoter region) for 663 transcripts with TSS assigned and found 197 that were methylated in the promoter region (Table S5), with a total of 162 5′-CT*A*T-3′ and 74 5′-G*A*N_7_TAY-3′/3′-CTN_7_ *A*TR-5′ motifs (located on both strands at the context site). Of these 197 transcripts, 103 are for non-coding RNAs (MPNs) and 89 correspond to ORFs. Fisher's exact test shows that there is a strong enrichment in methylation of MPNs promoters, with a *P*value of 8.98×10^−11^. No functional enrichment is found for genes or MPNs (considering coding genes that overlap) methylated at the promoter regions (Table S6a).

**Figure 5:**
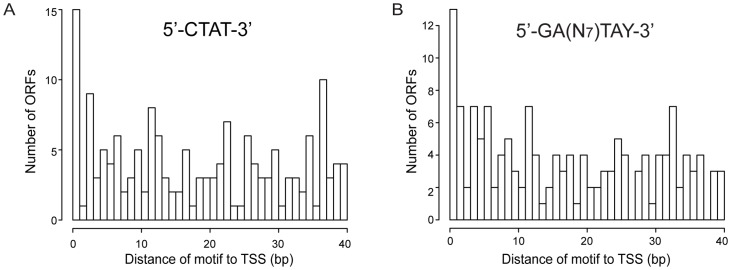
Histogram of distances of methylation motifs to TSS in the promoter regions. (*A*) Distances for the 5′-CT*A*T-3′ motif to TSS. (*B*) Distances for the 5′-G*A*N_7_TAY-3′/3′-CTN_7_ *A*TR-5′ motif to TSS.

Figure 5 shows the distribution in promoter regions of the distances from the methylation site (located upstream) to the TSS. Both motifs show that the highest frequency of methylation is at positions near the TSS and the Pribnow box (∼10–12 bases) (*P*value of 0.03 for the 5′-CT*A*T-3′ motif, and of 0.005 for the Type I motif). These results could suggest the methylation has a potential role in transcription by affecting interaction of the sigma70, or of specific transcription factors, with the promoter.

We have also investigated the methylation pattern of 5′UTR regions encompassing the DNA sequences between the TSS and the translational start codon longer than 40 bp (long 5′UTR). Ninety two of 154 ORFs that have long 5′UTR regions showed methylation (Table S7). COG analysis of genes showing methylation in long 5′UTRs (Table S6b) revealed that genes involved in defense mechanism were three times more represented, with a *P*value of 0.02. Interestingly, *mpn342* gene (M.MpnII) has a 56 bp 5′UTR with two5′-G*A*N_7_TAY-3′/3′-CTN_7_ *A*TR-5′ motifs, with 11 bp distance between the TSS and the motifs. As mentioned above, this gene could be responsible for methylating the 5′-G*A*N_7_TAY-3′/3′-CTN_7_ *A*TR-5′motif, suggesting an autoregulatory gene expression mechanism.

### Changes in methylation status as a function of growth phase

**Figure 6:**
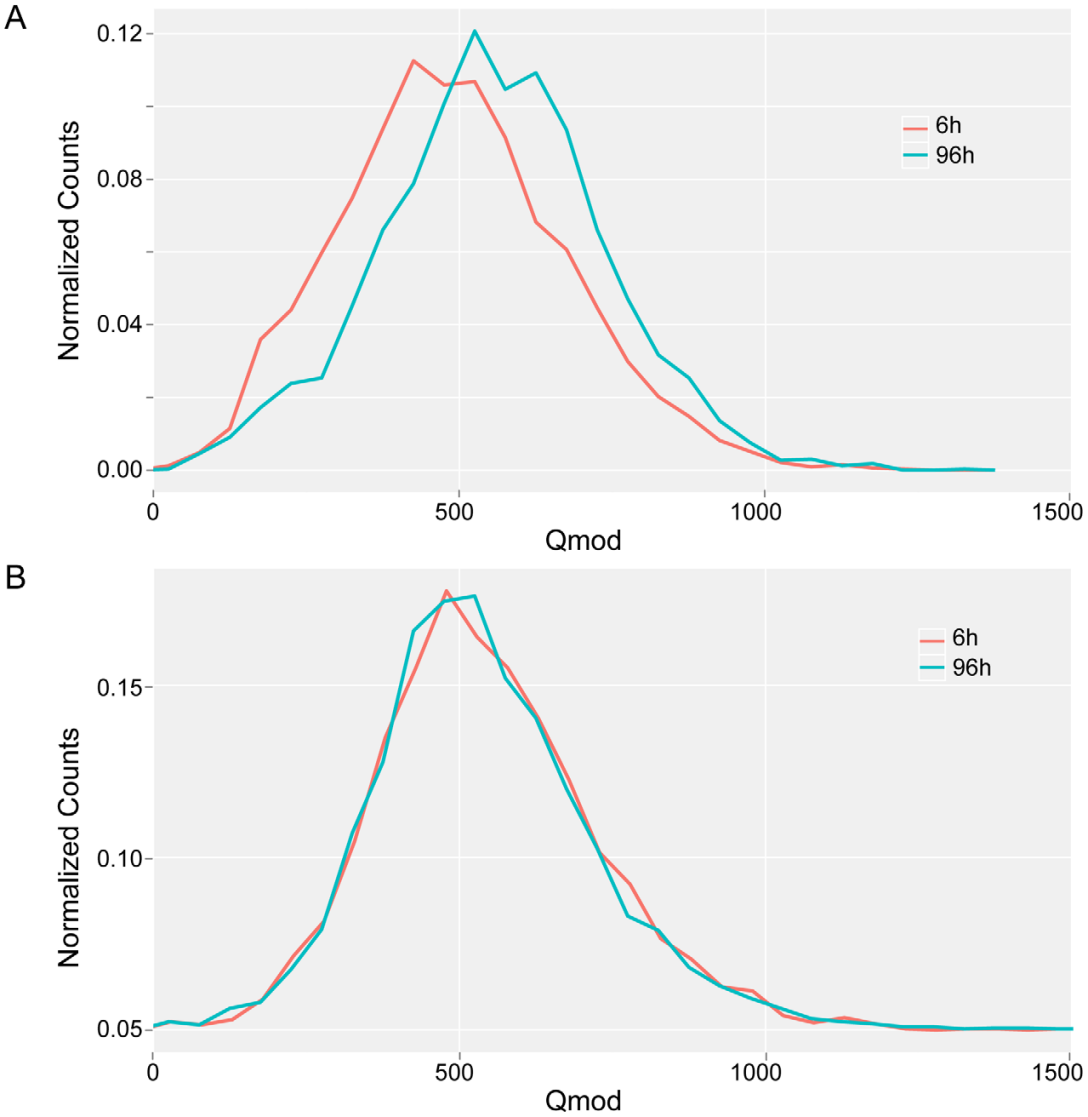
Qmod distributions. Qmod distributions of 5′-CT*A*T-3′ (*A*) and 5′-G*A*N_7_TAY-3′/3′-CTN_7_ *A*TR-5′ motifs (*B*) for *M. pneumoniae* genome at exponential (6 h, red line) and stationary (96 h, blue line) phases of growth.

Although the majority of the 5′-CT*A*T-3′ sites were methylated in both exponential (6 h) and stationary (96 h) phases, using the conservative Qmod threshold of 100, a few sites were identified as having significantly different Qmod values which would suggest a change in methylation fraction at the given sites. Figure 6 illustrates the decrease in the 5′CT*A*T-3′ Qmod distributions from stationary to exponential growth samples, while the 5′-G*A*N_7_TAY-3′/3′-CTN_7_ *A*TR-5′ Qmod distributions remain unchanged. This drop in the Qmod values points to a potential decrease in the methylation fraction at some 5′-CT*A*T-3′ sites at exponential growth as compared to stationary phase. To address this question of methylation changes at any given 5′-CT*A*T-3′ site between the growth phases at 6 h vs 96 h, we performed a direct comparison analysis between *M. pneumoniae* 6 h and 96 h. From this analysis, there are 35 5′-CT*A*T-3′ sites that were unmethylated at 6 h but became methylated by 96 h (Qmod≥60), indicating a change in methylation status between exponential and stationary phases of growth (Table S3). Twenty-five of the 35 methylation motifs are inside genes coding for membrane proteins, one in a 5′UTR, and the rest in intergenic regions. Analyzing the transcriptome for these 25 genes at 6 h and 96 h showed that their expression levels did not significantly change (Table S3), suggesting that this change in methylation state inside the genes is not related to the regulation of gene expression at different phases of growth. It was also observed that the fraction of methylation increased from 6 h to 96 h but not vice versa, further suggesting that the methylation in these regions are dependent on the phase of growth. It is noteworthy that M.MpnI reaches its maximal level of expression at exponential growth [39]. No general increase or decrease in gene expression was found associated with methylation. However, some specific cases, such as MPNs111, displayed an increase in promoter methylation with a significant decrease in transcript levels (fold change log_2_ = 2.93) (Table S8).

## Discussion

Previous analysis of DNA methylation in several mycoplasma species by HPLC revealed the presence of 6 mA in all of them, and of 5 mC in *Mycoplasma hyorhinis* [48]. Further studies performed in *Mycoplasma arthritidis*, to increase the efficiency of transformation, revealed methylated cytosine residues at 5′-AG*C*T-3′ and 5′-G*C*GC-3′ sites [49], [50]. Our current bioinformatic analysis in *M. pneumoniae* and *M. genitalium* did not find any evidence for 5 mC and only detected 6 mA. The study of proteome data (Table S1), together with a comparative analysis of gene conservation between these two species, suggest that there is an adenine methyltransferase (M.MpnI in *M. pneumoniae*, and M.MgI in *M. genitalium*) common to both genomes, and a putative Type I system in *M. pneumoniae* (*mpn342* for HsdM (M.MpnII), *mpn343* for HdsS (S.MpnII), and *mpn345* for HdsR). It also revealed other putative methyltransferases in *M. pneumoniae*, and parts of the Type I system identified at the genome level, but these were not detected by proteome analysis of extracts from the bacteria exposed to different stresses or along the growth curve [44], or from SDS gels. These results suggest that there are two functional methylation systems in *M. pneumoniae*, and one in *M. genitalium*.

We employed SMRT sequencing to test these hypotheses by comprehensively characterizing the methylomes of *M. pneumoniae* and *M. genitalium*. The unique capability of SMRT sequencing to have both base and DNA strand specificities in base modification detection enable whole microbial methylome profiling with unprecedented resolution. We identified an asymmetric adenine methylation motif common to both bacteria, 5′-CT*A*T-3′, and a Type I motif with methylated adenines in both strands (5′-G*A*N_7_TAY-3′/3′-CTN_7_ *A*TR-5′) found only in *M. pneumoniae*. The role of M.MpnI in the methylation of the 5′-CT*A*T-3′ motif was experimentally validated by expressing the methyltransferase in an *E. coli* strain devoid of methyltransferases [32].

The 5′-CT*A*T-3′motif was found enriched at the putative origin of replication (ORI) in *M. pneumoniae* as well as at two sites ∼100 kbs distant on both sides of the ORI which could be putative replication checkpoints, like the ψ sites described in *B. subtilis* [51]. The presence of two methylated 5′-CT*A*T-3′sites on the top and bottom strands at the mid-position of the putative DNA boxes at the ORI suggests a role for methylation in regulating DNA replication by M.MpnI. This hypothesis is reinforced by the fact that we did not find a restriction enzyme associated to this gene like in a classical *Eco*RI Type II system, similar to Dam methyltransferase in *E. coli*. The *ori*C of *E. coli* also contains an enriched region of methylated motifs (5′-G*A*TC-3′). SeqA preferentially binds to clusters of two or more hemimethylated 5′-G*A*TC-3′sites, delaying re-methylation and preventing binding of DnaA, which controls the initiation of DNA replication [52], [53]. No orthologous to *E. coli* SeqA protein has been identified in *M. pneumoniae*. However, a fundamental difference is found between the Dam system of *E. coli* and the M.MpnI methyltransferase of *M. pneumoniae*: in *M. pneumoniae*, only the one strand harboring the motif at any given genomic position is methylated, while in *E. coli*, both strands of the 5′-G*A*TC-3′ motif can be methylated. Thus, it is not expected that *M. pneumoniae* will use a similar system with SeqA as *E. coli* to control DNA duplication. In fact, the *M pneumoniae* firmicute relative *B. subtilis* also lacks *seqA* and *dam* orthologous but contains several other proteins, like Spo0, that regulate *oriC* [54], [55]. Interestingly, analysis of transcript levels along the growth curve shows that M.MpnI correlates with genes involved in transcription like *mpn515* (*rpoC*) and *mpn516* (*rpoB*), DNA duplication (*mpn003* [*gyrB*] and *mpn004* [*gyrA*]) and growth (ribosomal proteins like *mpn538*, *mpn539*, and *mpn540*) (Figure S1). This suggests a coordination between expression of M.MpnI and other genes involved in cell division and growth. Additionally, M.MpnI is the only methyltransferase that is essential for *M. pneumoniae* growth reinforcing its key role in cell cycle regulation.

Analysis of COG categories for ORFs located in regions enriched for 5′-CT*A*T-3′ showed that these are involved in virulence, similar to previously described adhesion genes regulated by DNA methylation in *E. coli* [19], [20]. We also found genes in *M. pneumoniae* methylated at their promoter or 5′UTR regions that have orthologous known to be regulated by methylation in other bacteria, such as *trpS* [56] and the SOS regulon [57] in *E. coli*, and ClpB in *Streptococus mutans*. However, no relationship between methylation and transcription levels was observed when we studied the correlation between M.MpnI and ORFs with methylation in their regulatory sequences. Nonetheless, this apparent lack of correlation may be due to the lack of synchrony in the bacterial population, which may therefore exhibit different phenotypic properties. The high number of antisense RNAs that show methylation in promoter regions could imply that in the absence of regulatory proteins, methylation could serve as a mechanism to regulate the expression of the antisense strand and, consequently, any overlapping genes.

In most active R-M systems, all sites recognized by the restriction enzyme are protected by methylation in order to prevent the microbe's own defense mechanism from damage to its genome. However, there are incidences in which a protein protects certain sites from restriction digestion or methylation. For example, a 5′-G*A*TC-3′sequence within the regulatory region of the *car* operon in *E. coli* was found to be protected from Dam methylation [58]. Indeed, CarP and IHF were shown to bind in this regulatory region and protect the 5′-G*A*TC-3′ site from methylation [59].We have detected unmethylated 5′-G*A*N_7_TAY-3′/3′-CTN_7_ *A*TR-5′ and 5′-CT*A*T-3′ sites, which could indicate that there is a protein interacting with these regions. A comparative study of the transcriptome at 6 h and 96 h in *M. pneumoniae* did not reveal any difference in transcription of genes containing unmethylated motifs when they are compared with the rest of the genes in the genome. Thus, these regions could be interaction sites for DNA-binding proteins that protect the DNA from methylation; in this case, methylation could play a role in transcription when the interacting protein is not occupying the region [60], [61]. However, interaction of structural elements that determine the structure of chromosome cannot be ruled out. Studies of protein occupancy could help to reveal why these regions are protected from methylation.

### Conclusion

In conclusion, using SMRT DNA sequencing, we were able to directly observe and analyze with single-base and strand resolution the genome-wide methylomes of *M. genitalium* and *M. pneumoniae*. The two strains share an analogous methlytransferase that targets the sequence 5′-CT*A*T-3. *M. pneumoniae* additionally has a Type I methyltransferase with a 5′-G*A*N_7_TAY-3′/5′-CTN_7_ *A*TR-3′ specificity. Together, these 2 motifs correspond to more than 99.9% of all sites directly detected by SMRT sequencing as modified. While ongoing work involving methyltransferase knock-out and over-expression studies are underway to help establish the relationship, this work demonstrates the unique capability of SMRT sequencing to directly sequence and profile the methylome of a whole microbial genome, allowing for unprecedented progress towards understanding the role of epigenomics in the world of prokaryotes.

## Materials and Methods

### Bacterial strains and growth conditions

*Escherichia coli* TOP 10 strain (Invitrogen) and *E. coli* ER2796 (DB24) [46] deficient in methyltransferases, also called DB24 (New England Biolabs), were grown at 37°C in LB broth or LB agar plates containing 100 µgml^−1^ ampicillin. The *M. genitalium* G-37 WT and *M. pneumoniae* M129 strains were grown in SP-4 and Hayflick media, respectively [62] at 37°C under 5% CO_2_ in tissue culture flasks (TPP). Cells were grown for 96 h for the stationary phase of growth. Alternatively, after 96 h of growth, the media was removed and replaced by fresh media, and the cells were scraped and re-grown for 6 h (exponential phase of growth).

Genomic DNA of *M. genitalium* and *M. pneumoniae* was isolated using the Illustra bacteria genomic Prep Mini Spin Kit (GE Healthcare). Plasmid DNA was obtained using the QIAprep Spin Miniprep Kit (Qiagen). All primers and plasmids used in this work are summarized in Table S9a and S9b. PCR products and digested fragments from agarose gels were purified using the QIAquick PCR purification Kit (Qiagen).

### Sequencing library preparation and SMRT sequencing

Genomic and plasmid samples of *M. genitalium* and *M. pneumoniae* were prepared for SMRT sequencing following standard SMRTbell template preparation protocols for base modification detection on the PacBio *RS* [63]. In brief, each genomic sample was used to construct two SMRTbell template libraries: a ∼500 bp randomly sheared insert library of native genomic DNA, and a whole-genome-amplified (WGA) library of the same insert size to remove any existing base modifications in the genomic DNA. The WGA sample served as a control. SMRT sequencing was performed using C2 chemistry. At 2–4 SMRTCells each, all samples achieved ∼500× average sequencing coverage across the genome.

### SMRT sequencing analysis

The principle of base modification detection using SMRT sequencing by synthesis was detailed in previous publications [31], [32]. The technique relies on the sensitivity of the polymerase kinetics to the DNA template structure as DNA synthesis is recorded in real time. It was observed that the time between base incorporations, or interpulse duration (IPD), is on average longer when the nucleotide incorporation occurs opposite of a methylated base in the DNA template, as compared to an incorporation opposite of a canonical base.

In previous studies, the analysis involved computing the ratio of the mean IPD of the native sample to the mean IPD of the WGA control sample for every reference template position, and setting a threshold to call certain template positions as methylated. The data analysis implemented here uses a t-test with a log-normal distribution model for the IPDs and associated *P*value at every position for identifying the methylated sites. The null hypothesis in this analysis is that the IPDs from the native and WGA samples are part of the same population, and the alternate hypothesis is that the native set of IPDs stems from a population with larger IPDs, namely from incorporations opposite of a methylated rather than canonical template base. A threshold value of 100 for the log-transformed *P*value from the t-test (called Qmod = −10log(*P*value)) at each reference position was used for assigning the given position as methylated. The value of 100 was chosen based on the Qmod distribution observed in the data, where there was a clear bimodal distribution arising from unmodified background and modified positions. Furthermore, a Qmod≥100 corresponds to better than the Bonferroni corrected *P*value of 0.0001 for the 816 kb genome.

To detect relative changes of the methylation status between samples grown for different time periods, the two native samples were directly compared against each other, rather than against a WGA control sample, thus highlighting the methylome difference between those samples. This analysis is performed after whole methylome analysis of the genome of interest. Hence, all sites of the discovered motifs were used as the n independent test sites giving a Bonferroni corrected *P*value of better than 0.01 (0.0067) at Qmod≥60. Plots were made using Circos [64].

Both modes of analysis were carried out using SMRT Portal (http://www.smrtcommunity.com/SMRT-Analysis/Software/SMRT-Portal), while sequence motif cluster analysis was done using Pacific Biosciences's Motif Finder (http://www.smrtcommunity.com/CodeShare_Project?id=a1q70000000GtatAAC). Data sets containing kinetic values for each reference position and DNA strand are available at http://www.pacbiodevnet.com/Share/Datasets/Senar-et-al.

### Molecular cloning

*M. pneumoniae mpn107* gene was obtained by PCR using genomic DNA as template and specific primers (Table S9a). 5′-end oligonucleotides incorporated a *Pst*I site followed by the sequence 5′-TTAAGG-3′ (to terminate translation of the *lac* α-peptide reading frame of the pRSS plasmid vector and to reinitiate translation of the cloned methyltransferse (MTase) genes, followed by an eight nucleotide spacer sequence 5′-TTAATCAT-3′ and sequences complementary to the 5′-end of the relevant MTase coding sequence. 3′-end oligonucleotides were complementary to the 3′-end of the MTase coding sequences, including translation termination codons and a *Bam*HI restriction site. Since the TGA codon encodes tryptophan in *Mycoplasma* but an opal stop codon in *E. coli*, the *mpn198* and *mpn108* genes having several opal codons were codon-transformed and synthesized by GeneScript. After PCR amplification, the different genes were cloned into a *Pst*I-*Bam*HI digested pRSS vector. The resulting vectors were termed pRSS*107*, pRSS*198*, and pRSS*108* (Table S9b).

### Confirmation of methylation motifs by SMRT sequencing

The vectors described above were used to transform the *E. coli* deficient in methyltransferases ER2796 strain (kindly provided by R. Roberts, NEB). The plasmid DNA of every transformed strain was analyzed by SMRT sequencing as described previously [32].

### Transcriptome data

Transcriptional start sites of the *M.pneumoniae* transcriptome have been described recently [42]. This information was used to define the 5′-UTR (RNA sequences from transcriptional start site to translational start codon). Transcription levels of *M. pneumoniae* genes at 6 h and 96 h were previously determined by tiling and ultrasequencing [43]. These data were used to study the relation between methylation and transcription in *M. pneumoniae* (Table S1).

## Supporting Information

Figure S1 Heatmap of expression data of 13 genes involved in transcription and replication obtained by RNA-seq. The heatmap shows the correlation in gene expression among these genes, using data from 12 different time points from exponential and stationary growth phases. *mpn003* and *mpn004* codify for the subunits of the DNA gyrase; *mpn198* (M.MpnI); *mpn515* and *mpn516* for the subunits of the RNA polymerase; MPN538, MPN539, MPN540 and MPN541 for ribosomal proteins; *mpn001* for the DnaN subunit of the DNA polymerase; *mpn686* codifies for the DnaA helicase; *mpn024* for the delta subunit of the RNA polymerase and *mpn192* for a ribosomal protein.
(PDF)

Table S1 Transcriptome and proteome data. MPNr is the nomenclature used for ribosomal RNAs and MPNt is the nomenclature used for tRNAs. MPNs are the non coding RNAs.
(PDF)

Table S2 Enriched regions for 5′-G*A*N_7_TAY-3′/3′-CTN_7_ *A*TR-5′ (a) and 5′-CT*A*T-3′ (b) motifs. Table S2c is the legend for the functions assigned to the different COG categories.
(PDF)

Table S3 ORFs with a 5′-CT*A*T-3′ motif that changed from non-methylated to methylated state from 6 to 96 h.
(PDF)

Table S4 Functional analysis of genes located in “hot spots of methylation”. COG categories of genes located in enriched regions for 5′-CT*A*T-3′ (a) and 5′-G*A*N_7_TAY-3′/3′-CTN_7_ *A*TR-5′ (b) motifs have been compared to those categories in whole genome by using Fisher's test. A functional enrichment is considered significant when the *P*value<0.05.
(PDF)

Table S5 Methylation in promoter sequences. Genomic regions 40 bp upstream from the TSS are considered as putative promoter sequences. This table shows the 197 out 663 ORFs with assigned TSS that showed methylation at the promoter region. First column indicates the ORF name. The rest of columns indicate the sequence of the motifs and the genome positions, as well as, the strand for these motifs. The two last columns indicate the function and the COG category respectively. If the ORF is a new identified RNA (MPNs) the overlapping ORF is indicated in the function column and the COG category of the overlapping ORF is annotated in the last column.
(PDF)

Table S6 Functional enrichment of COG categories for promoter (a) and 5′ UTR regions (b). The functional enrichment was measured by comparing all the function of all the ORFs that have an associated promoter sequence or 5′UTR with those that have these regions methylated. Enrichment is measured by Fisher's test and significant enrichment is considered when the *P*value<0.05 (marked with “*”).
(PDF)

Table S7 Methylation in 5′UTR regions. Start and End regions are the genome positions comprising the 5′UTR region. Column named “motifs” indicates the number of motifs identified. Str is the abbreviation for strand and indicates the gene orientation (“+” forward strand, “−” reverse strand). The rest of columns indicate the sequence of the motifs and the genome positions as well as, the strand for these motifs. The two last columns indicate the function and the COG category respectively.
(PDF)

Table S8 Study of transcription in ORFs containing 5′-CT*A*T-3′ motifs that showed changes in methylation between 96 and 6 h. First column indicates methylated positions that show an increase of methylation in different phases of growth. Second column shows the strand where the motif is found. Third column indicates the IPD ratio between 6 h and 96 h. Fourth column, the Qmod values. Two next columns, show the genome location. Columns named as regions indicate if the region is intergenic (IG) coding or promoter. The columns named as “name” indicate the protein name of overlapping ORFs and the ones named as “category” the associated COG categories. The values of gene expression determined by Deep Sequencing Strand Specific (DSSS) at 6 and 96 hours are indicated in the columns named as ExpDSS 6 h and ExpDSS 96 hours respectively. Two last columns indicate changes in gene expression. Significant increase in expression is considered when the difference in expression between 96 and 6 hours higher than 1,5 exp increase). Significant decrease is considered when this difference is lower than −1,5 (Exp decrease) and no significant changes are considered when the value of the difference is between −1.5 and 1.5.
(PDF)

Table S9 a) Table of primers used in this study. b) Table of vectors used to clone and express different putative methyltransferases of *M. pneumoniae* in ER2796 strain.
(PDF)
