# Supplementary figures and images for: Comprehensive Methylome Characterization of Mycoplasma genitalium and Mycoplasma pneumoniae at Single-Base Resolution
Source: PLoS Genet. 2013 Jan 3;9(1):e1003191. doi: 10.1371/journal.pgen.1003191 (PMC3536716; doi:10.1371/journal.pgen.1003191)

Figure S1

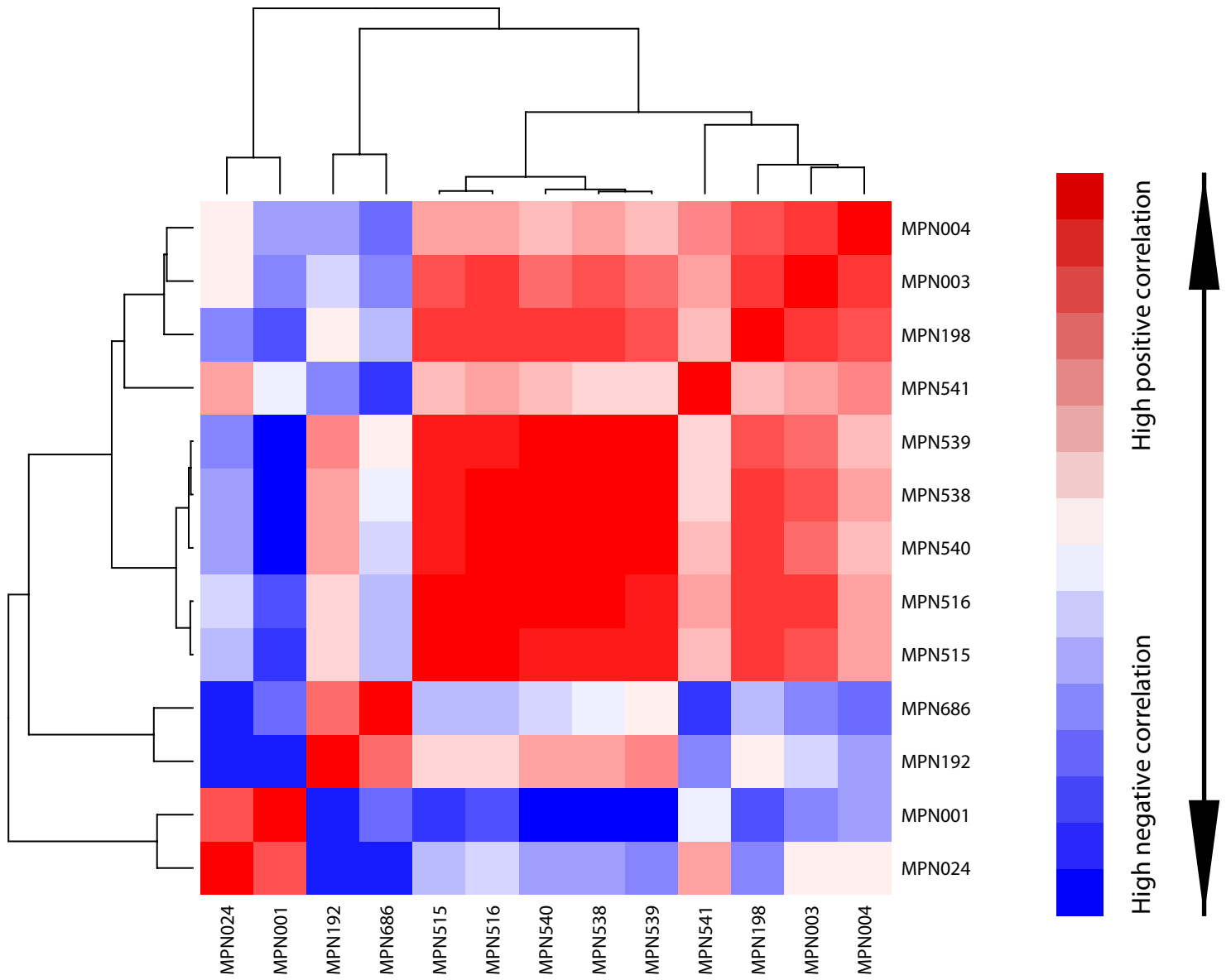

Supplement: Figure S1 — Heatmap of expression data of 13 genes involved in transcription and replication obtained by RNA-seq. The heatmap shows the correlation in gene expression among these genes, using data from 12 different time points from exponential and stationary growth phases. mpn003 and mpn004 codify for the subunits of the DNA gyrase; mpn198 (M.MpnI); mpn515 and mpn516 for the subunits of the RNA polymerase; MPN538, MPN539, MPN540 and MPN541 for ribosomal proteins; mpn001 for the DnaN subunit of the DNA polymerase; mpn686 codifies for the DnaA helicase; mpn024 for the delta subunit of the RNA polymerase and mpn192 for a ribosomal protein. (PDF) [file pgen.1003191.s001.pdf]
