# Supplementary material for: Comprehensive Methylome Characterization of Mycoplasma genitalium and Mycoplasma pneumoniae at Single-Base Resolution
Source: PLoS Genet. 2013 Jan 3;9(1):e1003191. doi: 10.1371/journal.pgen.1003191 (PMC3536716; doi:10.1371/journal.pgen.1003191)
Supplement: Table S1 — Transcriptome and proteome data. MPNr is the nomenclature used for ribosomal RNAs and MPNt is the nomenclature used for tRNAs. MPNs are the non coding RNAs. (PDF) [file pgen.1003191.s002.pdf]

Table S1 – Transcriptome and Proteome data

ORF	RNA micro-arrays 6h	RNA micro-array 96h	RNAseq 6h	RNAseq 96h	Tiling 37 average expression level	Protein copy number per cell 6h	Protein copy number per cell 96h	Protein name	Function
MPN001	12.5	13.4	8.9087	10.42740109	9.95	398.66	393.22	dnaN	DNA polymerase III subunit beta (EC 2.7.7.7)
MPN002	11.3	11.4	6.8444	6.795225738	9.42	13.01	12.78	xdj1,CbpA	DnaJ-like protein
MPN003	13.6	12.5	6.7150	7.78103857	9.71	92.56	96.48	gyrB	DNA gyrase subunit B (EC 5.99.1.3)
MPN004	11.9	11.5	6.9252	6.838304364	9.39	95.03	93.07	gyrA	DNA gyrase subunit A (EC 5.99.1.3)
MPN005	10.3	9.9	6.6187	6.851848769	9.64	83.1	90.77	serS	Seryl-tRNA synthetase (EC 6.1.1.11) (Seryl-tRNA(Ser/Sec) synthetase) (Serine–tRNA ligase) (SerRS)
MPN006	9.9	10.2	6.0342	5.748015003	9.28	26.39	25.22	tmk	Thymidylate kinase (EC 2.7.4.9) (dTMP kinase)
MPN007	9.5	10.3	7.0834	6.271874295	8.96	10.91	9.61	holB	DNA polymerase III subunit delta'
MPN008	8.3	7.9	5.1922	5.462858245	7.83	8.3	7.62	thdF	tRNA modification GTPase mnmE (EC 3.6.-.-)
MPN009	6.8	6.7	4.7531	4.429751741	6.82	7.06	5.75	TatD,yabD, YcfH	Uncharacterized deoxyribonuclease MPN_009 (EC 3.1.21.-)
MPN010	4.9	6.6	7.7564	8.83932842	8.27	0	0		Uncharacterized protein MPN_010
MPN011	8.9	9.6	9.2438	9.518287731	6.9062	12.47	16.89		Conserved hypothetical lipoprotein MPN_011
MPN012	9.2	10.3	9.7538	10.42637605	7.9905	27.64	7.5		Conserved hypothetical protein MPN_012
MPN013	12.0	12.7	7.8809	8.618795769	9.02	12.07	13.11		Uncharacterized protein MPN_013
MPN014	8.9	11.1	3.0188	3.763948015	6.58	0	0		Contains the TOPRIM domain of the DNA primase
MPN015	11.1	10.3	10.5949	10.21408275	8.763	26.85	16.33	rimK	Ribosomal protein S6 modification enzyme
MPN016	10.2	9.0	10.7101	10.94214718	8.9158	2.3	22.41	rimK	Ribosomal protein S6 modification enzyme
MPN017	8.2	6.8	8.6475	7.956958496	7.5571	15.91	10.2	mtd1	Methylenetetrahydrofolate dehydrogenase (EC 1.5.1.5); Methenyltetrahydrofolate cyclohydrolase (EC 3.5.4.9)
MPN018	10.8	11.2	6.4800	7.250034431	9.94	31.31	30.94	pmd1	Putative ABC transporter ATP-binding protein MPN_018
MPN019	10.6	11.3	7.4597	8.528589585	10	24.25	31.1	msbA	Putative ABC transporter ATP-binding protein MPN_019
MPN020	11.3	11.0	7.0314	7.766739618	9.32	58.9	53.8	yb95, HepA, RapA, YabA	Uncharacterized ATP-dependent helicase (EC 3.6.1.-)
MPN021	9.0	9.6	6.6949	8.099278767	9.5	81.6	95.96	dnaJ	Chaperone protein dnaJ
MPN022	9.3	8.3	7.5200	8.135010724	9.66	56.17	79.06	pip	Putative proline iminopeptidase (PIP) (EC 3.4.11.5) (Prolyl aminopeptidase) (PAP)
MPN023	9.1	8.8	5.2865	5.530917249	8.05	15.23	16.71	metS	Methionyl-tRNA synthetase (EC 6.1.1.10) (Methionine–tRNA ligase) (MetRS)
MPN024	13.6	14.5	9.3863	11.0006638	11.2	149.87	133.65	rpoE	DNA-directed RNA polymerase delta subunit
MPN025	12.5	13.4	8.4749	9.017263208	10.9	611.7	556.28	tsr, fba	Fructose-bisphosphate aldolase (FBP aldolase) (FBPA) (EC 4.1.2.13)
MPN026	11.2	9.9	6.2341	5.81465702	8.56	17.26	12.52	EngD..	GTP-dependent nucleic acid-binding protein engD
MPN027	9.5	9.7	7.5821	7.499503779	7.616	12.86	9.18	rimL	Ribosomal-protein alanineacetyltransferase
MPN028	9.7	10.7	7.6328	8.578367331	8.7	0	0	trsB	Uncharacterized glycosyl transferase
MPN029	11.8	12.8	6.7727	8.06109854	10.1	211.63	261.13	efp	Elongation factor P (EF-P)
MPN030	11.5	11.5	7.5935	7.85230699	9.83	10.48	12.82	NusB	Transcription termination/antitermination protein NusB
MPN031	8.8	9.0	5.9707	6.108167189	8.66	13.66	11.69		Uncharacterized protein MPN031
MPN032	11.1	12.0	9.6630	9.317938247	9.4232	0	0	YfkM	DJ-1/PfpI family intracellular protease
MPN033	10.9	11.6	9.6259	9.454946026	9.1181	26.3	26.93	upp	Uracil phosphoribosyltransferase (EC 2.4.2.9) (UMP pyrophosphorylase) (UPRTase)
MPN034	9.9	10.8	8.8726	9.125419407	9.2545	50.33	47.55	polC	DNA polymerase III polC-type (PolIII) (EC 2.7.7.7)
MPN035	8.1	7.2	4.6379	5.057558978	7.64	0	0		Conserved hypothetical protein MPN_035
MPN036	13.5	13.0	9.1417	10.00009517	10.5	13.84	8.26		Conserved hypothetical protein MPN_036
MPN037	9.1	7.9	4.7367	4.529153659	6.68	0	0		Uncharacterized protein MPN_037
MPN038	9.7	10.0	9.2396	8.824242908	6.9846	0	0		Uncharacterized protein MPN_038
MPN039	12.0	12.3	7.1430	7.841499932	8.56	0	0		Conserved hypothetical protein MPN_039
MPN040	6.2	5.6	5.4268	5.538785821	5.9627	0	0		Conserved hypothetical protein MPN_040
MPN041	6.6	NA	0.3188	3.316871187	6.63	0	0		Conserved hypothetical protein MPN_041
MPN042	7.8	8.7	2.7456	4.341014268	6.44	0	0		Conserved hypothetical protein MPN_042
MPN043	11.9	10.9	5.6578	6.047551956	9.04	46.86	66.38	glpF	Glycerol uptake facilitator protein
MPN044	12.3	13.1	10.5883	11.83619518	10.861	112.97	119.44	tdk	Thymidine kinase (EC 2.7.1.21)
MPN045	9.9	9.8	6.1594	7.301165986	9.52	54.1	52.5	hisS	Histidyl-tRNA synthetase (EC 6.1.1.21) (Histidine–tRNA ligase) (HisRS)
MPN046	7.4	5.2	4.8848	5.11560031	7.83	17.12	19.65	aspS	Aspartyl-tRNA synthetase (EC 6.1.1.12) (Aspartate–tRNA ligase) (AspRS)
MPN047	8.8	10.0	4.3615	5.382111537	8.55	10.3	8	PncB	Nicotinate phosphoribosyl transferase (EC 2.4.2.12)
MPN048	8.5	8.5	4.2172	3.482658024	6.32	0	0		Uncharacterized protein MPN_048
MPN049	8.5	9.1	5.1917	4.917562629	6.56	0	0		Uncharacterized protein MPN_049
MPN050	10.1	11.7	9.0633	9.921079422	9.7754	134.86	146.26	glpK	Glycerol kinase (EC 2.7.1.30) (ATP:glycerol 3-phosphotransferase) (GK)
MPN051	10.8	12.4	9.9929	11.1065607	10	188.63	161.29	glpD	Glycerol-3-phospate dehydrogenase (EC 1.1.99.5)
MPN052	12.4	13.0	8.4248	9.959713184	10	343.63	400.29		Uncharacterized lipoprotein MPN_052
MPN053	14.4	14.9	9.5667	10.01355717	10.3	472.99	603.33	ptsH	Phosphocarrier protein HPr (EC 2.7.11.-) (Histidine-containing protein)
MPN054	6.4	7.7	1.6296	3.579035264	5.79	0	0		Conserved hypothetical lipoprotein MPN_054

Continued on next page

Table S1 – Transcriptome and Proteome data – continued from previous page

ORF	RNA micro-arrays 6h	RNA micro-array 96h	RNAseq 6h	RNAseq 96h	Tiling 37 average expression level	Protein copy number per cell 6h	Protein copy number per cell 96h	Protein name	Function
MPN055	8.7	8.8	6.6907	7.108242597	7.99	60.72	50.85	potA	Spermidine/putrescine import ATP-binding protein potA (EC 3.6.3.31)
MPN056	10.5	8.3	5.8116	5.231735313	7.26	0	0	potB	Spermidine/putrescine transport system permease protein potB homolog
MPN057	10.7	9.4	4.7149	3.698925079	7.29	0	0	potI	Spermidine/putrescine transport system permease protein potC homolog
MPN058	7.9	5.6	6.8770	7.596720703	6.81	20.06	22.92	potD	Conserved hypothetical lipoprotein MPN_058
MPN059	12.3	11.7	7.0971	8.138795443	9.68	27.51	25.79	Gcp	DNA-binding/iron metalloprotein/AP endonuclease
MPN060	11.0	9.8	7.3624	7.672368011	8.57	37.42	33.56	metX	S-adenosylmethionine synthetase (EC 2.5.1.6) (Methionine adenosyltransferase) (AdoMet synthetase) (MAT)
MPN061	10.3	10.2	9.7132	9.794444112	10.103	101.01	94.8	ffh	Signal recognition particle protein (Fifty-four homolog)
MPN062	11.3	12.3	8.1661	9.068716635	10.7	481.66	533.49	deoC	Purine nucleoside phosphorylase deoD-type (PNP) (EC 2.4.2.1)
MPN063	12.2	11.7	7.7854	9.341413561	10.4	230.35	246.97	deoD	Deoxyribose-phosphate aldolase (EC 4.1.2.4) (Phosphodeoxyriboaldolase) (Deoxyriboaldolase) (DERA)
MPN064	10.8	9.9	7.5929	8.591796213	10.1	183.21	231.49	deoC	Thymidine phosphorylase (EC 2.4.2.4) (TdRPase)
MPN065	12.0	10.8	8.1344	7.575288743	10.1	18.31	16.68	deoA	Cytidine deaminase (CDA) (EC 3.5.4.5) (Cytidine aminohydrolase)
MPN066	11.8	9.2	6.6005	6.247250458	9.06	76.59	90.09	cdd	Phosphomannomutase (PMM) (EC 5.4.2.8)
MPN067	12.6	10.9	10.7536	9.388360565	8.756	176.58	193.15	cpsG	Transcription antitermination factor
MPN068	13.1	12.9	10.9497	10.67791826	9.4114	13.16	14.33	secE	Preprotein translocase subunit secE
MPN069	10.0	10.7	13.7370	13.6962204	10.59	15.66	6.61	rpmG2	50S ribosomal protein L33 type 2
MPN070	10.4	10.9	8.6793	9.752546239	8.6961	7.96	4.26		Uncharacterized protein MPN_070
MPN071	8.5	9.5	7.6176	6.659296397	7.4779	15.04	12.54	yabC	16S rRNA (cytidine1402-2'-O)-methyltransferase
MPN072	9.7	10.3	8.4662	7.651703531	8.2474	12.56	9.08	rnmV	Ribonuclease M5 (EC 3.1.26.8)
MPN073	11.4	12.3	8.4498	9.277133555	9.2789	128.56	149.77	prs	Ribose-phosphate pyrophosphokinase (RPPK) (EC 2.7.6.1) (Phosphoribosyl pyrophosphate synthetase) (P-Rib-PP synthetase) (PRPP synthetase)
MPN074	9.6	10.7	9.4614	9.332785966	8.5764	3.35	0	smpB	tmRNA-binding protein
MPN075	9.0	8.4	4.3564	5.167584369	6.86	7.29	5.11	ywdF	Uncharacterized glycosyltransferase family 2 (EC 2.4.-.-)
MPN076	8.3	10.0	9.9413	10.67736587	9.8043	240.08	233.95	uhpT	Hexose phosphate transport protein
MPN077	9.5	11.3	9.8453	9.837490384	8.5736	87.14	108.58	uhpT	Hexosephosphate transport protein
MPN078	9.8	9.1	3.9252	4.692020534	7.3	15.65	19.67	fruA	PTS system fructose-specific EIIABC component (EIIABC-Fru) (2.7.1.69)
MPN079	7.7	6.6	3.5346	4.333720175	6.89	13.68	12.78	fruK	Fructose 1-phosphate kinase (EC 2.7.1.56)
MPN080	9.3	9.7	4.8627	4.963934824	7.08	12.62	24.05		MPN074
MPN081	8.3	7.4	4.5290	3.37064193	7.4	10.78	12.68	glnQ	Gln transport; ATP-binding protein
MPN082	8.3	10.9	7.2040	7.921299421	10.5	290.1	311.53	tklB	Transketolase (TK) (EC 2.2.1.1)
MPN083	11.3	12.9	9.0888	10.35086258	11	12.54	9.72		Uncharacterized lipoprotein MPN_083
MPN084	11.0	13.0	9.3182	10.92791575	10.1	10.59	17.2		Conserved hypothetical lipoprotein MPN_084
MPN085	9.2	11.0	6.9872	8.810108433	8.1	0	0.41		Uncharacterized protein MPN_085
MPN086	9.6	8.4	3.2503	5.254441374	6.31	0	0		Uncharacterized protein MPN_086
MPN087	8.0	8.8	4.8338	6.068791301	6.41	0	0		Uncharacterized protein MPN_087
MPN088	7.8	7.9	2.7465	4.51068529	6.58	0	0		Uncharacterized protein MPN_088
MPN089	11.2	11.3	5.6909	6.522610103	7.84	0	0	hsdS	Putative type-1 restriction enzyme specificity protein MPN_089 (S.mpnORFAP) (Type I restriction enzyme specificity protein MPN_089) (S protein)
MPN090	10.3	11.1	7.6213	8.479006671	8.49	21.88	4.82		Uncharacterized protein MPN_090
MPN091	13.9	13.5	3.5740	3.905697055	10.5	0	0		Conserved hypothetical protein protein MPN_091
MPN092	9.8	8.9	1.1453	2.548712999	8.6	0	0		Putative mgpC-like protein MPN_092
MPN093	11.0	11.9	3.8109	4.258311236	9.03	34.81	44.01		Putative mgpC-like protein MPN_093
MPN094	10.8	11.4	8.1266	8.987012909	8.47	50.45	18.38		UPF0134 protein MPN_094
MPN095	10.4	11.8	5.6902	7.442072692	9.07	0.12	9.24		Uncharacterized amino acid permease
MPN096	9.4	10.7	5.5055	6.911074978	8.5	0	0		Uncharacterized amino acid permease
MPN097	10.5	11.5	5.3222	6.046835678	7.93	0	0.06		Conserved hypothetical lipoprotein MPN_097
MPN098	7.0	7.8	2.7696	2.050129258	6.7	12.93	15.14		Conserved hypothetical lipoprotein MPN_098
MPN099	12.7	13.1	3.9832	4.845503119	7.93	88.51	70.22		Putative adhesin P1-like protein MPN_099
MPN100	9.3	12.5	7.9289	9.360048695	8.91	18.7	14.08		Uncharacterized protein MPN_100
MPN101	13.0	13.9	3.7380	4.196268033	8.47	227.16	207.06		Uncharacterized protein MPN_101
MPN102	5.9	6.8	0.8848	2.103934569	6.67	0	8.8		Putative mgpC-like protein MPN_102
MPN103	5.3	10.3	2.2578	4.421818658	6.1433	0	0		Uncharacterized protein MPN_103
MPN104	11.1	11.8	6.7194	7.602791392	8.92	23.12	16.84		Uncharacterized protein MPN_104
MPN105	10.2	11.7	7.1899	8.371312962	9.49	60.24	54.03	pheS	Phenylalanyl-tRNA synthetase alpha chain (EC 6.1.1.20) (Phenylalanine–tRNA ligase alpha chain) (PheRS)
MPN106	10.9	10.4	5.6473	6.30713593	8.76	68.06	65.77	pheT	Phenylalanyl-tRNA synthetase beta chain (EC 6.1.1.20) (Phenylalanine–tRNA ligase beta chain) (PheRS)
MPN107	9.6	10.3	4.6604	6.058956285	7.42	0	0		Possible uncharacterized DNA methylase

Continued on next page

Table S1 – Transcriptome and Proteome data – continued from previous page

ORF	RNA micro-arrays 6h	RNA micro-array 96h	RNAseq 6h	RNAseq 96h	Tiling 37 average expression level	Protein copy number per cell 6h	Protein copy number per cell 96h	Protein name	Function
MPN108	9.3	8.4	4.6601	5.812938626	6.9	0	0		Uncharacterized adenine-specific methylase MPN_108 (EC 2.1.1.72)
MPN109	9.0	10.3	6.9305	7.863888317	7.3	42.02	41.08		Uncharacterized protein MPN_109
MPN110	7.0	7.5	4.1001	5.167440067	6.48	0	0		Conserved hypothetical protein MPN_110
MPN111	7.7	7.3	4.1708	6.01732576	6.58	0	0		Uncharacterized adenine-specific methylase MPN_111 (EC 2.1.1.72)
MPN112	8.1	12.2	5.2524	8.092906313	9.81	0	0		Major facilitator family protein CglT
MPN113	6.7	8.1	3.4046	4.17127706	7.54	0	0		Putative uncharacterized permease MPN_113
MPN114	4.9	5.6	3.0046	4.563364026	7.53	0	0		Putative acetyltransferase MPN_114 (EC 2.3.1.-)
MPN115	12.0	11.7	9.5341	9.851829264	9.97	134.42	124.74	infC	Translation initiation factor IF-3
MPN116	12.5	10.5	8.0275	7.924984222	9.66	16.7	14.47	rpmI	50S ribosomal protein L35
MPN117	10.1	8.3	7.0145	6.444200878	9.15	31.35	33.28	rpLT	50S ribosomal protein L20
MPN118	7.3	10.6	9.2653	9.487432729	8.4946	15.68	12.62	rnhC	Ribonuclease HIII
MPN119	10.8	10.6	7.1649	7.482848743	9.33	103.49	100.82		DnaJ-like, terminal organelle protein
MPN120	11.7	12.1	7.4756	8.552234511	9.42	261.04	222.59	grpE	Heat shock protein GrpE
MPN121	11.5	12.1	7.0030	7.542159442	9.11	113.89	110.15	mpn121	Uncharacterized protein MPN_121
MPN122	10.9	10.2	6.1829	5.898115529	8.77	12.34	9.27	parB	DNA topoisomerase 4 subunit B (EC 5.99.1.-) (Topoisomerase IV subunit B)
MPN123	10.1	9.6	5.1300	5.362686678	8.11	8.98	7.56	parC	DNA topoisomerase 4 subunit A (EC 5.99.1.-) (Topoisomerase IV subunit A)
MPN124	10.3	12.2	10.1397	10.22201945	9.3973	111.36	62.67	hrcA	Heat-inducible transcription repressor hrcA
MPN125	8.7	9.8	4.7814	5.336283837	8.13	9.7	8.02	uvrC	UvrABC system protein C (Protein uvrC) (Excinuclease ABC subunit C)
MPN126	9.5	12.4	7.6068	9.665289259	10.5	29.3	31.13	ysnB	Putative metallophosphoesterase YsnB (EC 3.1.4.-)
MPN127	10.1	11.1	6.6141	6.961725474	8.75	2.31	0.88		Uncharacterized protein MPN_127
MPN128	8.4	8.3	5.6507	5.682180625	7.24	85.32	89.03		Uncharacterized protein MPN_128
MPN129	11.1	11.5	7.6815	8.206883523	8.72	0	0		Uncharacterized protein MPN_129
MPN130	13.8	13.4	8.4620	9.67542116	8.71	68.9	25.12		Uncharacterized protein MPN_130
MPN131	7.0	5.9	0.5397	1.711391669	7.3	0	0		Putative adhesin P1-like protein MPN_131
MPN132	7.4	8.9	4.1161	4.901234687	7.12	87.68	48.39		Putative adhesin P1-like protein MPN_132
MPN133	9.9	10.7	7.7617	8.240515757	9	2.61	1.48		Ca2+-dependent cytotoxic nuclease of Mycoplasma
MPN134	9.9	8.7	5.7310	6.739541262	8.59	139.47	122.39	ugpC	Putative ABC sn-glycerol-3-phosphate transporter ATP-binding protein ugpC
MPN135	10.4	9.9	6.1493	6.374134044	8.66	11.56	7.77	ugpA	sn-glycerol-3-phosphate transport system permease protein ugpA
MPN136	9.7	7.6	5.7303	5.781293195	8.19	10	18.35	ugpE	sn-glycerol-3-phosphate transport system permease protein ugpE
MPN137	11.0	12.1	10.8755	10.85606033	8.6079	0	1.84		Uncharacterized protein MPN_137
MPN138	12.2	12.8	12.2176	12.28869477	9.425	0.48	0		Uncharacterized protein MPN_138
MPN139	11.1	11.9	11.3783	11.0054959	9.4051	8.4	16.44		Uncharacterized protein MPN_139
MPN140	10.9	11.4	7.4501	7.547720204	9.65	186.67	160.18	ytqI	Oligoribonuclease (DHH family phosphoesterases)
MPN141	14.5	15.0	8.4766	8.852374582	11.7	312.74	269.96	mgpA	Adhesin P1 (Cytadhesin P1) (Attachment protein)
MPN142	12.2	13.0	8.3973	8.001675091	11.5	121.02	130.37		Mgp-operon protein 3 (Mgp3) (ORF-3 protein)
MPN143	8.7	8.2	3.8976	0.022025416	7.79	0	0		Uncharacterized protein MPN_143
MPN144	9.6	9.8	4.6840	5.213187888	9.14	189.94	174.57		Putative adhesin P1-like protein MPN_144
MPN145	9.2	9.3	6.5805	6.192579323	7.64	0	0		Uncharacterized protein MPN_145
MPN146	12.8	15.5	9.3329	11.18913477	10.3	0	0		Conserved hypothetical protein MPN_146
MPN147	8.8	10.1	6.8806	6.366981651	7.33	0	0		Conserved hypothetical protein MPN_147
MPN148	12.5	12.4	9.3190	9.520699026	8.34	8.34	4.23		Conserved hypothetical protein MPN_148
MPN149	7.1	8.6	4.8015	5.231891824	8.99	68.78	7.85		Putative mgpC-like protein MPN_149
MPN150	9.7	11.6	1.2921	3.781493248	9.08	0	0		Putative mgpC-like protein MPN_150
MPN151	7.1	9.5	7.2928	8.776205786	8.23	16.33	16.44		Uncharacterized protein MPN_151
MPN152	7.2	7.2	4.2032	5.067256264	7.22	27.86	32.6		Uncharacterized lipoprotein MPN_152
MPN153	11.2	10.9	6.6640	7.587109077	8.97	105.96	104.9		superfamily I DNA and RNA helicase and helicase subunits-like protein (EC 3.6.1.-)
MPN154	11.7	8.1	6.8083	5.475153641	7.72	170.56	149.49	nusA	Transcription elongation protein nusA
MPN155	11.6	10.9	5.9634	6.518262235	9.25	89.83	79.65	infB	Translation initiation factor IF-2
MPN156	11.7	10.7	5.8686	5.248264243	9.08	16.47	18.33	rbfA	Ribosome-binding factor A
MPN157	13.8	12.2	7.8612	7.582945691	9.87	48.25	48.52		Conserved hypothetical protein MPN_157
MPN158	10.7	8.2	4.4951	4.656634263	8.45	12.41	11.25	yaaC	Putative riboflavin biosynthesis protein ribF [Includes: Riboflavin kinase (EC 2.7.1.26) (Flavokinase); FMN adenylyltransferase (EC 2.7.7.2) (FAD pyrophosphorylase) (FAD synthetase)]
MPN159	9.6	9.4	7.1363	7.908740252	9.08	11.94	19.9	tlyC	Hemolysisn, TlyC-like protein
MPN160	9.1	8.5	6.2944	5.925780795	8.17	8.58	8.97		Conserved hypothetical protein MPN_160
MPN161	10.7	11.3	6.9736	8.33624728	8.76	67.44	55.24		Conserved hypothetical protein MPN_161
MPN162	10.7	10.2	8.6593	7.145224444	9.11	69.6	30.57		Conserved hypothetical protein MPN_162
MPN163	10.5	10.9	7.9043	9.635968055	8.62	31.59	22.79		Conserved hypothetical protein MPN_163
MPN164	12.9	9.6	6.1265	4.725599037	8.25	92.89	80.44	rpsJ	30S ribosomal protein S10- NusE termination antitermination factor
MPN165	12.5	10.7	5.9240	5.332758294	8.04	73.11	101.18	rplC	50S ribosomal protein L3
MPN166	14.2	10.9	5.8448	4.551830468	7.65	115.14	120.72	rplD	50S ribosomal protein L4
MPN167	13.7	11.0	5.6162	4.297728156	7.35	161.85	159.79	rplW	50S ribosomal protein L23
MPN168	13.1	10.6	6.6712	5.036205175	7.91	76.59	102.63	rplB	50S ribosomal protein L2
MPN169	14.1	11.4	7.5622	4.833197272	8.55	40.57	61.8	rpsS	30S ribosomal protein S19
MPN170	13.7	10.7	7.2166	4.500139484	8.5	51.61	44.49	rplV	50S ribosomal protein L22

Continued on next page

Table S1 – Transcriptome and Proteome data – continued from previous page									
ORF	RNA micro-arrays 6h	RNA micro-array 96h	RNAseq 6h	RNAseq 96h	Tiling 37 average expression level	Protein copy number per cell 6h	Protein copy number per cell 96h	Protein name	Function
MPN171	13.6	10.6	7.4179	4.354370175	8.43	87.46	93.11	rpsC	30S ribosomal protein S3
MPN172	13.1	10.6	5.1253	4.540566438	8.3	39.76	48.19	rplP	50S ribosomal protein L16
MPN173	14.2	11.1	7.6131	5.075148052	8	35.5	30.34	rpmC	50S ribosomal protein L29
MPN174	11.4	9.9	6.5755	5.570652525	8.38	44.54	56.94	rpsQ	30S ribosomal protein S17
MPN175	10.3	8.5	4.7399	3.202558389	8.52	24.95	31.33	rplN	50S ribosomal protein L14
MPN176	14.7	11.3	7.0599	5.278324122	8.83	83.92	92.34	rplX	50S ribosomal protein L24
MPN177	12.7	8.8	6.4325	4.738655439	8.06	101.93	121.09	rplE	50S ribosomal protein L5
MPN178	12.8	10.5	6.7546	6.533873131	8.63	52.46	52.58	rpsN	30S ribosomal protein S14 type Z
MPN179	13.8	11.9	6.9506	6.260598463	8.84	41.29	48.38	rpsH	30S ribosomal protein S8
MPN180	13.0	10.4	6.5727	5.508056407	8.59	96.38	103.69	rplF	50S ribosomal protein L6
MPN181	13.2	9.9	8.0267	8.005179806	8.96	60.7	68.48	rplR	50S ribosomal protein L18
MPN182	14.3	10.7	6.9459	5.667142715	9.58	86.57	91.08	rpsE	30S ribosomal protein S5
MPN183	13.0	9.4	6.7520	7.496727855	8.87	56.95	44.74	rplO	50S ribosomal protein L15
MPN184	11.8	9.5	6.4802	6.245311478	8.68	23.92	20.14	secY	Preprotein translocase subunit secY
MPN185	11.7	9.3	5.4476	4.81883822	8.06	66.28	62.01	adk	Adenylate kinase (AK) (EC 2.7.4.3) (ATP-AMP transphosphorylase)
MPN186	11.0	6.8	8.9812	8.933159896	8.63	20.34	11.19	map	Methionine aminopeptidase (MAP) (EC 3.4.11.18) (Peptidase M)
MPN187	10.3	6.7	6.0220	5.767260175	8.07	33.88	33.83	infA	Translation initiation factor IF-1
MPN188	10.7	9.9	9.3184	10.34302295	10.4	0.42	16.01	rpmJ	50S ribosomal protein L36
MPN189	13.7	12.1	4.1418	6.618084093	9.35	72.76	67.03	rpsM	30S ribosomal protein S13
MPN190	13.5	11.1	6.9803	5.822695248	9.63	66.45	54.19	rpsK	30S ribosomal protein S11
MPN191	13.5	10.8	7.1970	6.765110115	9.15	329.64	325.62	rpoA	DNA-directed RNA polymerase subunit alpha (RNAP subunit alpha) (EC 2.7.7.6) (Transcriptase subunit alpha)
MPN192	13.2	11.9	6.8911	6.523450942	8.63	44.69	33.43	rplQ	50S ribosomal protein L17
MPN193	12.0	10.2	6.6328	6.137970624	8.38	40.55	32.98	cbiO 1	Cobalt/nickel import ATP-binding protein CbiO 1 (EC 3.6.3.-)
MPN194	11.2	11.4	6.2200	7.362162934	9.03	47.93	41.8	CbiO 2	Cobalt/nickelimport ATP-binding protein CbiO 2 (EC 3.6.3.-)
MPN195	10.6	10.4	6.9720	7.286398563	8.82	23.75	25.95	cbiQ	Similar to Cobalt/nickel ABC transporter CbiQ
MPN196	10.7	9.3	8.1419	9.076739048	8.43	5.82	3.27	hisT	tRNA pseudouridine synthase A, TruA (EC 5.4.99.12) (tRNA-uridine isomerase I) (tRNA pseudouridylate synthase I)
MPN197	11.4	10.2	5.9626	5.624604963	8.2	143.46	146.9	pepF	Oligoendopeptidase F homolog (EC 3.4.24.-)
MPN198	8.7	6.9	6.6593	6.440796779	7.16	4.61	2.36	mte1	Uncharacterized adenine-specific methylase MPN_198 (EC 2.1.1.72) (M.mpnIP)
MPN199	9.2	8.7	6.6917	7.209925956	7.31	2.6	3.37		Conserved hypothetical lipoprotein MPN_199
MPN200	10.0	10.5	6.5446	7.993910452	7.97	31.63	29.7		Conserved hypothetical lipoprotein MPN_200
MPN201	11.0	11.0	6.2861	7.231795532	8.32	2.73	0		Putative type-1 restriction enzyme specificity protein MPN_201 (S.mpnORFFP) (Type I restriction enzyme specificity protein MPN_201) (S protein)
MPN202	8.5	8.0	5.7103	6.523838145	8.82	105.83	130.17		Putative adhesin P1-like protein MPN_202
MPN203	12.8	12.6	5.9828	6.125535661	7.84	0	0		Putative adhesin P1-like protein MPN_203
MPN204	15.7	15.3	6.9709	8.583424067	8.67	61.69	22.85		Conserved hypothetical protein MPN_204
MPN205	11.5	12.1	5.1153	5.546181389	8.04	181.26	175.6		Uncharacterized protein MPN_205
MPN206	5.6	6.3	NAME?	4.771372065	5.6932	0	0		Uncharacterized protein MPN_206
MPN207	13.0	12.8	7.9287	8.444698365	10.7	430.31	442.76	ptsG	PTS system glucose-specific EIICBA component (EIICBA-Glc) (EII-Glc/EIII-Glc) (EC 2.7.1.69)
MPN208	11.7	11.7	9.9918	10.92877528	10.3	117.17	106.58	rpsB	30S ribosomal protein S2
MPN209	9.4	7.2	6.1006	6.307027091	7.87	13.59	10.57	mgtA	Probable cation-transporting P-type ATPase (EC 3.6.3.-)
MPN210	12.0	11.2	7.2344	7.829198106	9.25	135.46	138.07	secA	Protein translocase subunit secA
MPN211	10.2	10.0	5.5771	5.960308618	8.46	41.78	37.4	uvrB	UvrABC system protein B (Protein uvrB) (Excinuclease ABC subunit B)
MPN212	13.9	14.4	10.3174	11.08528433	9.8				Conserved hypothetical protein MPN_212
MPN213	12.9	12.5	8.3788	8.142478174	9.01	19.36	54.02		Conserved hypothetical protein MPN_213
MPN214	10.5	12.2	10.0103	10.30498816	8.5253	11.15	35.23	OppA??	Conserved hypothetical protein MPN_214
MPN215	13.2	10.4	6.8637	6.483690625	8.59	40.78	43.69	oppB	Oligopeptide transport system permease protein oppB
MPN216	11.9	9.1	6.1466	5.620663221	8.38	60.6	59.48	oppC	Oligopeptide transport system permease protein oppC
MPN217	12.9	9.5	5.3897	4.397098822	8.3	50.59	48.73	oppD	Oligopeptide transport ATP-binding protein oppD
MPN218	12.2	10.0	5.3325	3.646431997	7.59	72.58	94.7	oppF	Oligopeptide transport ATP-binding protein oppF
MPN219	11.0	11.2	7.3478	9.008997079	10.2	88.59	87.66	rplK	50S ribosomal protein L11
MPN220	11.2	10.4	7.4165	7.836540893	9.8	115.04	126.97	rplA	50S ribosomal protein L1
MPN221	10.9	11.1	7.1075	6.866314525	8.61	32.16	35.19	pth	Peptidyl-tRNA hydrolase (PTH) (EC 3.1.1.29)
MPN222	11.4	10.1	5.6337	5.656306711	8.41	7.26	5.77	yacA	tRNA(Ile)-lysidine synthase (EC 6.3.4.-) (tRNA(Ile)-lysidine synthetase) (tRNA(Ile)-2-lysyl-cytidine synthase)
MPN223	10.5	8.9	6.6324	7.000980014	8.43	16.72	16.38	hprK	HPr kinase/phosphorylase (HPrK/P) (EC 2.7.11.-) (EC 2.7.4.-) (HPr(Ser) kinase/phosphorylase)
MPN224	11.9	11.5	7.8740	9.154761283	9.2	15.34	7.89	lgt	Prolipoprotein diacylglyceryl transferase (EC 2.4.99.-)

Continued on next page

Table S1 – Transcriptome and Proteome data – continued from previous page

ORF	RNA micro-arrays 6h	RNA micro-array 96h	RNAseq 6h	RNAseq 96h	Tiling 37 average expression level	Protein copy number per cell 6h	Protein copy number per cell 96h	Protein name	Function
MPN225	13.5	12.7	10.5206	10.32570286	9.64	78.34	69.37	rpsL	30S ribosomal protein S12
MPN226	13.3	11.0	7.9615	7.228945989	8.91	102.23	96.53	rpsG	30S ribosomal protein S7
MPN227	13.2	9.8	7.2427	6.503591869	8.83	231.47	243.73	fus	Elongation factor G (EF-G)
MPN228	11.5	9.7	7.7787	7.878321004	10	138.62	142.07	rpsF	30S ribosomal protein S6
MPN229	14.5	12.0	7.9310	7.580367458	9.48	103.69	90.2	ssbA	Single-stranded DNA-binding protein (SSB) (Helix-destabilizing protein)
MPN230	11.4	9.5	6.4896	6.514049976	8.73	119.29	115.06	rpsR	30S ribosomal protein S18
MPN231	14.4	10.7	7.2239	6.293235561	9.06	40.78	50.13	rplI	50S ribosomal protein L9
MPN232	11.8	8.7	5.5071	5.684763242	7.65	30.9	29.81	dnaB	Replicative DNA helicase (EC 3.6.1.-)
MPN233	11.2	9.6	5.1037	5.158679636	7.45	73.12	40.81		Uncharacterized lipoprotein MPN_233
MPN234	9.1	9.2	6.9905	6.472180023	8.11	3.64	0		Conserved hypothetical lipoprotein MPN_234
MPN235	9.5	10.8	5.9229	5.695531837	8.24	3.78	0.85	ung	Uracil-DNA glycosylase (UDG) (EC 3.2.2.-)
MPN236	11.7	10.8	6.9845	7.334048991	8.5	24.36	23.73	gatC	Aspartyl/glutamyl-tRNA(Asn/Gln) amidotransferase subunit C (Asp/Glu-ADT subunit C) (EC 6.3.5.-)
MPN237	10.5	9.5	5.9292	5.746781524	7.86	69.43	73.56	gatA	Glutamyl-tRNA(Gln) amidotransferase subunit A (Glu-ADT subunit A) (EC 6.3.5.-)
MPN238	10.0	8.0	5.6723	6.002810534	7.47	44.84	42.3	gatB	Aspartyl/glutamyl-tRNA(Asn/Gln) amidotransferase subunit B (Asp/Glu-ADT subunit B) (EC 6.3.5.-)
MPN239	11.5	12.2	8.3601	9.269053924	10	92.46	103.53	gntR	Probable HTH-type transcriptional regulator gntR
MPN240	11.0	11.7	6.3574	6.884736761	9.34	84.72	83.49	trxB	Thioredoxin reductase (TRXR) (EC 1.8.1.9)
MPN241	8.6	9.7	5.1357	5.165163587	7.61	260.06	3.37	WhiA	Transcription factor with WhiA C-terminal domain
MPN242	11.3	13.1	8.4038	10.25179978	9.7	0	0	secG	Probable protein-export membrane protein secG
MPN243	10.4	10.5	8.8481	9.139157622	10.5	93.09	98.33	vacB	Ribonuclease R (RNase R) (EC 3.1.-.-) (VacB protein homolog)
MPN244	11.5	11.5	6.9780	6.525063062	9.26	33.97	35.31	disA	DNA integrity scanning protein DisA
MPN245	9.0	10.2	8.4981	9.789968642	8.8543	97.97	94.81	def	Peptide deformylase (PDF) (EC 3.5.1.88) (Polypeptide deformylase)
MPN246	11.4	12.7	7.9619	9.752787685	9.75	145.6	134.84	gmk	Guanylate kinase (EC 2.7.4.8) (GMP kinase)
MPN247	10.2	11.6	7.3608	7.257007041	9.56	63.33	51.16	ptc1	Putative protein phosphatase PrpC (EC 3.1.3.16)
MPN248	10.2	9.7	6.2386	5.757907194	8.38	12.53	11.14	prkC	Putative serine/threonine-protein kinase PrkC (EC 2.7.11.1)
MPN249	8.9	8.4	4.0103	4.47146427	7.32	0	0	CpgA	Putative ribosome biogenesis GTPase rsgA (EC 3.6.1.-)
MPN250	10.8	11.9	8.2698	9.4722519	10.8	432.42	463.01	pgiB	Glucose-6-phosphate isomerase (GPI) (EC 5.3.1.9) (Phosphoglucose isomerase) (PGI) (Phosphohexose isomerase) (PHI)
MPN251	11.9	12.1	6.8971	7.423865733	10.2	20.71	22.89	cfxE	Probable ribulose-phosphate 3-epimerase (EC 5.1.3.1) (Pentose-5-phosphate 3-epimerase) (PPE) (R5P3E)
MPN252	10.8	10.1	6.6340	7.081413565	9.26	68.1	77.15	asnS	Asparaginyl-tRNA synthetase (EC 6.1.1.22) (Asparagine–tRNA ligase) (AsnRS)
MPN253	8.2	7.9	5.8146	5.362140442	8.03	0	0	pgsA	CDP-diacylglycerol–glycerol-3-phosphate 3-phosphatidyltransferase (EC 2.7.8.5) (Phosphatidylglycerophosphate synthase) (PGP synthase)
MPN254	12.2	12.3	7.4485	7.665394093	8.78	52.97	93.44	cinA	Putative competence-damage inducible protein CinA
MPN255	9.6	8.5	7.7666	7.840188711	8.3	111.66	129.88	ygbP	2-C-methyl-D-erythritol 4-phosphate cytidylyltransferase-like
MPN256	12.5	10.1	6.7494	6.5152854	7.72	86.95	93.12		Conserved hypothetical protein MPN_256
MPN257	10.5	8.9	7.1881	5.348754875	7.25	36.52	38.62	galE	UDP-glucose 4-epimerase (EC 5.1.3.2) (UDP-galactose 4-epimerase)
MPN258	10.2	10.4	8.2415	9.547462715	10.3	145.85	158.03	mglA	Sugar (ribose/galactose) ABC transporter ATP-binding subunit
MPN259	12.2	12.8	8.5415	9.498934677	9.93	51.44	67.86	rbsC1	Sugar (ribose/galactose) ABC transporter permease subunit, but probably C-terminal
MPN260	12.6	12.8	8.1097	8.624615012	9.89	91.42	63.85	rbsC	Sugar (ribose/galactose) ABC transporter permease subunit
MPN261	12.4	12.0	8.0152	8.876044157	9.66	133.99	132.88	topA	DNA topoisomerase 1 (EC 5.99.1.2) (DNA topoisomerase I) (Omega-protein) (Relaxing enzyme) (Untwisting enzyme) (Swivelase)
MPN262	10.1	9.7	6.9019	7.164684213	8.03	34.51	32.63		Uncharacterized protein, similar to reticulocyte binding protein
MPN263	12.6	14.0	10.2076	11.06816989	10.7	956.35	832.69	trx	Thioredoxin (Trx)
MPN264	11.5	12.0	7.2512	7.876613018	9.64	41.59	26.59		Uncharacterized hydrolase, haloacid dehalogenase-like family protein (EC 3.1.-.-)
MPN265	10.2	10.5	9.7992	8.527061547	8.0755	46.48	59.88	trpS	Tryptophanyl-tRNA synthetase (EC 6.1.1.2) (Tryptophan–tRNA ligase) (TrpRS)
MPN266	10.1	10.9	7.3576	9.427041749	10	128.95	123.69	spxA	Transcriptional regulatory protein Spx

Continued on next page

Table S1 – Transcriptome and Proteome data – continued from previous page

ORF	RNA micro-arrays 6h	RNA micro-array 96h	RNAseq 6h	RNAseq 96h	Tiling 37 average expression level	Protein copy number per cell 6h	Protein copy number per cell 96h	Protein name	Function
MPN267	9.9	11.0	7.5097	8.305136856	10.1	278.98	266.07	ppnK	Probable inorganic polyphosphate/ATP-NAD kinase (Poly(P)/ATP NAD kinase) (EC 2.7.1.23)
MPN268	12.0	12.6	7.8655	7.385307889	9.27	59.03	60.5	ptsG	Putative phosphotransferase enzyme IIB component MPN_268 (EC 2.7.1.69)
MPN269	8.1	8.9	6.6186	7.226259782	8	25.57	27.25	ymdA	Endoribonuclease Y (Rnase Y)
MPN270	8.5	10.3	7.0802	7.47886218	7.66	0	0		Putative oligopeptide ABC transporter (permease protein)
MPN271	11.6	12.3	6.0810	7.003308706	8.92	18.87	7.71		Conserved hypothetical lipoprotein MPN_271
MPN272	11.9	13.2	8.2799	10.02445308	9.09	54.37	39.83		Uncharacterized protein MPN_272
MPN273	11.1	11.4	11.2106	10.84259325	9.0096	275.02	253.17	hit1	Diadenosine tetraphosphate Ap4A hydrolase (EC 3.6.1.41)
MPN274	11.4	11.1	9.3099	10.06198041	8.4768	0	0		Putative ABC transporter
MPN275	11.7	12.3	5.5401	7.529918551	9.51	57.02	43.2	yaaK	Putative uncharacterized protein yaaK
MPN276	8.6	8.4	8.9969	8.14876542	8.4432	24.81	29.88		Conserved hypothetical protein MPN_276
MPN277	8.2	8.7	6.2505	6.621667689	9.16	91.89	84.58	lysS	Lysyl-tRNA synthetase (EC 6.1.1.6) (Lysine–tRNA ligase) (LysRS)
MPN278	9.2	9.4	4.7923	5.073441	7.9	14.33	10.5	yefE	UDP-galactopyranose mutase (EC 5.4.99.9)
MPN279	7.2	6.5	4.0961	3.950244483	7.87	16.09	21.36	lepA	GTP-binding protein lepA
MPN280	12.9	10.5	7.1221	6.259394244	8.2	115.22	106.77	rnjA	Ribonuclease J1 (RNase J1) (EC 3.1.-.-)
MPN281	11.3	13.3	6.9483	8.714617345	9.66	29.7	26.83		Conserved hypothetical lipoprotein MPN_281
MPN282	11.1	11.5	6.6951	7.416683194	7.64	0	0		Conserved hypothetical protein MPN_282
MPN283	11.2	12.3	7.7609	8.919090926	8.12	0	0		Uncharacterized protein MPN_283
MPN284	11.0	10.5	5.3539	6.504306876	7.04	98.78	118.54		Uncharacterized lipoprotein MPN_284
MPN285	9.4	10.4	6.2355	5.973690682	9.19	4.41	0	prrB	Putative type-1 restriction enzyme specificity protein MPN_285 (S.mpnORFGP) (Type I restriction enzyme specificity protein MPN_285) (S protein)
MPN286	8.4	8.5	2.3210	3.249538104	8.31	136.58	71.29		Putative adhesin P1-like protein MPN_286
MPN287	10.9	12.0	5.8681	7.427747484	8.28	0	6.41		Uncharacterized protein MPN_287
MPN288	10.2	8.8	5.1707	5.558396899	6.83	56.52	66.25		Conserved hypothetical lipoprotein MPN_288
MPN289	5.5	11.9	5.4395	5.352490524	6.96	0	0	hsdS1B	Putative type-1 restriction enzyme specificity protein MPN_289 (S.mpnORFEBP) (Type I restriction enzyme specificity protein MPN_289) (S protein)
MPN290	9.7	10.2	3.9003	3.915598675	6.84	0	0		Putative type-1 restriction enzyme specificity protein MPN_290 (S.mpnORFEAP) (Type I restriction enzyme specificity protein MPN_290) (S protein)
MPN291	9.5	10.1	10.0910	9.434981136	7.9325	18.41	18.41		Probable O-sialoglycoprotein endopeptidase
MPN292	8.6	9.9	9.3500	9.017767439	8.5295	9.58	9.31	rluD	Ribosomal large subunit pseudouridine synthase D (EC 5.4.99.-)
MPN293	10.9	12.5	9.8535	10.42931616	9.067	0	0.22	lsp	Lipoprotein signal peptidase (EC 3.4.23.36) (Prolipoprotein signal peptidase) (Signal peptidase II) (SPase II)
MPN294	11.8	13.1	9.5354	10.65023531	10.4	351.29	311.65	pfpI	SImilar to intracellular protease ThiJ/PfpI;
MPN295	12.2	12.9	9.0397	10.22381469	10.4	404.69	387.09		Conserved hypothetical protein MPN_295
MPN296	14.1	13.6	9.0449	9.453366493	10.9	27.64	24.46	rpsU	30S ribosomal protein S21
MPN297	12.9	12.2	9.7778	9.513159577	10.4	112.43	118.12	gpsB	Similar to cell-division initiation protein DivIVA
MPN298	12.6	11.1	7.8927	7.273139068	9.65	0	3.43	acpS	Holo-[acyl-carrier-protein] synthase (Holo-ACP synthase) (EC 2.7.8.7) (4'-phosphopantetheinyl transferase acpS)
MPN299	9.0	7.7	6.1152	5.323783682	9.46	62.4	62.36	plsC	Probable 1-acyl-sn-glycerol-3-phosphate acyltransferase (1-AGP acyltransferase) (1-AGPAT) (EC 2.3.1.51) (Lysophosphatidic acid acyltransferase) (LPAAT)
MPN300	10.4	9.9	6.8203	6.39532661	8.11	10.98	8.31	dyr	Dihydrofolate reductase (EC 1.5.1.3) and segregation and condensation protein A, ScpA
MPN301	11.0	10.2	4.5537	4.139896535	8.37	17.63	17.91	scpB	Segregation and condensation protein B
MPN302	10.5	12.4	8.2808	9.383157327	11.4	224.48	243.62	pfk	6-phosphofructokinase (Phosphofructokinase) (EC 2.7.1.11) (Phosphohexokinase)
MPN303	11.8	12.6	8.0524	8.379981937	11	320.57	359.48	pyk	Pyruvate kinase (PK) (EC 2.7.1.40)
MPN304	10.9	13.3	9.0011	10.80775093	11.6	5.7	6.98	arcA	Putative arginine deiminase (ADI) (EC 3.5.3.6) (Arginine dihydrolase) (AD) N-terminal fragment
MPN305	9.7	11.6	8.3811	10.33319947	10.7	0	0	arcA	Putative arginine deiminase (ADI) (EC 3.5.3.6) (Arginine dihydrolase) (AD) C-terminal fragment
MPN306	9.6	10.9	3.4836	4.388403179	9.24	0	0	argI	Ornithine carbamoyltransferase, catabolic (OTCase) (EC 2.1.3.3)
MPN307	12.1	13.5	6.6231	7.447124669	9.98	6.56	17.02	arcC	Carbamate kinase-like protein (EC 2.7.2.2)
MPN308	11.7	11.8	6.7349	7.619050623	9.62	7.42	16.55		Uncharacterized amino acid pernease
MPN309	11.6	12.2	10.1356	9.932465309	10.8	69.12	68.92	p65	Proline-rich P65 protein
MPN310	12.5	10.8	7.8180	7.482144455	8.73	146.38	148.75	hmw2	Cytadherence high molecular weight protein 2 (Cytadherence accessory protein 2)
MPN311	10.9	10.3	7.8888	7.653615344	8.5	81.74	93.59		Conserved hypothetical protein MPN_311
MPN312	9.6	9.2	7.0352	6.09233305	8.37	21.38	22.81		Uncharacterized protein MPN_312
MPN313	7.2	9.1	7.2915	8.532033742	6.3631	0	0		Conserved hypothetical protein MPN_313

Continued on next page

Table S1 – Transcriptome and Proteome data – continued from previous page									
ORF	RNA micro-arrays 6h	RNA micro-array 96h	RNAseq 6h	RNAseq 96h	Tiling 37 average expression level	Protein copy number per cell 6h	Protein copy number per cell 96h	Protein name	Function
MPN314	15.5	15.1	10.4355	11.91279155	11.5	977.59	809.35	mraZ	Protein mraZ
MPN315	13.6	13.9	10.2350	10.23637305	10.6	217.05	171.58	mraW	S-adenosyl-L-methionine-dependent methyltransferase mraW (EC 2.1.1.-)
MPN316	14.0	13.9	10.1798	10.10217716	10	11.72	6.74	ftsA	Cell division protein ftsA
MPN317	13.9	12.8	9.2316	9.261811516	9.52	17.99	6.74	ftsZ	Cell division protein ftsZ
MPN318	10.6	10.9	7.8577	8.267327014	8.75	15.28	14.19		Putative amino acid pernease
MPN319	13.1	10.7	5.9842	5.253275729	8.63	7.16	10.34		Putative amino acid pernease
MPN320	12.1	13.1	7.3580	10.42070329	10.6	480.14	400.85	thyA	Thymidylate synthase (TS) (TSase) (EC 2.1.1.45)
MPN321	11.8	13.0	8.2226	10.08929906	11.2	212.48	221.03	dhfr	Dihydrofolate reductase (EC 1.5.1.3)
MPN322	11.6	12.1	7.7761	9.888896918	10.9	827.73	921.4	nrdF	Ribonucleoside-diphosphate reductase subunit beta (EC 1.17.4.1) (Ribonucleotide reductase small subunit)
MPN323	9.8	11.0	8.2062	10.27086366	10.8	492.23	664.84	nrdI	Protein nrdI
MPN324	11.4	12.4	7.1757	8.570768469	10.7	737.03	778.53	nrdE	Ribonucleoside-diphosphate reductase subunit alpha (EC 1.17.4.1) (Ribonucleotide reductase)
MPN325	15.0	13.8	10.2303	10.52977999	10.8	51.96	79.44	rplU	50S ribosomal protein L21
MPN326	12.8	11.5	8.5331	8.004530082	10.1	26.78	16.34	ysxB	Predicted ribosomal protein
MPN327	13.5	11.7	7.4455	7.239591614	9.8	38.11	54.74	rpl27	50S ribosomal protein L27
MPN328	11.8	9.4	7.1565	6.929996371	9.06	53.84	46.92	nfo	Probable endonuclease 4 (EC 3.1.21.2) (Endonuclease IV) (Endodeoxyribonuclease IV)
MPN329	10.4	8.6	7.4904	6.789412346	8.36	11.98	12.22	fur	Ferric uptake regulation protein
MPN330	10.0	9.2	5.3692	4.425883085	7.83	11.34	12.17		Conserved hypothetical protein MPN_330
MPN331	12.4	13.3	8.4771	8.979760926	10.5	215.57	226.96	tig	Trigger factor (TF) (EC 5.2.1.8)
MPN332	13.7	14.2	8.6565	9.710644983	11.1	82.05	76.23	lon	ATP-dependent protease La (EC 3.4.21.53)
MPN333	10.7	11.2	6.2922	6.618382268	8.59	0	0		Putative ABC transport system permease protein
MPN334	9.3	9.1	6.4011	6.945244646	7.99	0	0	bcrA	Putative ABC transporter ATP-binding protein MPN_334
MPN335	7.0	6.2	4.6749	4.896100499	7.26	0	0		Putative ABC transport system permease protein
MPN336	0.0	0.0	8.8355	9.145322207	7.6667	35.16	34.87	nadD	Nicotinate-nucleotide adenylyltransferase (EC 2.7.7.18), phosphopantetheine adenylyltransferase (EC 2.7.7.3) and choline-phosphate cytidylyltransferase (EC 2.7.7.15)
MPN337	9.6	9.6	6.6039	7.871131513	7.37	30.54	25.21		Conserved hypothetical protein MPN_337
MPN338	8.7	8.9	5.8358	6.581124781	7.39	38.35	47.1		Conserved hypothetical protein MPN_338
MPN339	7.6	7.4	5.6966	5.491212497	7.02	2.74	2.24		Conserved hypothetical protein MPN_339
MPN340	10.0	8.7	4.8025	4.712409521	6.63	8.63	9.39		Probable DNA helicase I homolog (EC 3.6.1.-)
MPN341	9.4	8.8	5.0314	5.347448434	6.8	2.1	1.51		Probable DNA helicase I homolog (EC 3.6.1.-)
MPN342	12.0	11.2	7.8154	8.29038936	7.96	19.53	9.16	hsdM	Putative type I restriction enzyme HsdM (EC 2.1.1.72)
MPN343	9.6	9.3	5.3050	5.648484904	8.38	2.73	0		Putative type-1 restriction enzyme specificity protein MPN_343 (S.mpnORFDP) (Type I restriction enzyme specificity protein MPN_343) (S protein)
MPN344	13.3	14.6	7.3809	8.076288938	9.25	10.48	25.22		Uncharacterized protein MPN_344
MPN345	8.8	7.8	4.9655	4.545051808	6.61	0	0	hsdR	Putative type-1 restriction enzyme mpnORFDP R protein part 2 (EC 3.1.21.3) (Putative type I restriction enzyme mpnORFDP R protein part 2) (mpnORFDBP)
MPN346	7.9	9.1	4.6665	6.768324818	6.86	0	0		Uncharacterized protein MPN_346
MPN347	8.0	8.4	3.1450	2.938133465	6.14	0	0	hsdR	Putative type I restriction enzyme mpnORFDP R protein part 1 (EC 3.1.21.3) (mpnORFDAP)
MPN348	7.2	8.9	6.3650	7.362417834	7.5	18.32	13.53	mthfs	5-formyltetrahydrofolate cyclo-ligase (EC 6.3.3.2)
MPN349	9.4	10.0	4.4477	6.330432247	7.6	56.83	51.56	ymdB	M-pesterase
MPN350	10.7	10.3	9.6770	9.505016427	7.8381	0.55	0	plsY	Putative glycerol-3-phosphate acyltransferase PlsY (EC 2.3.1.15)
MPN351	12.1	9.9	9.4396	7.140336267	7.0886	0	0	trmK	tRNA: m1A22 methyltransferase TmrK
MPN352	14.0	11.6	10.9686	9.927154484	8.2674	111.19	107.74	sigA	RNA polymerase sigma factor rpoD (Sigma-A) (EC 2.7.7.6)
MPN353	9.9	10.0	8.3768	7.847467161	7.6365	20.46	19.27	dnaG	DNA primase (EC 2.7.7.-)
MPN354	11.1	11.2	8.7925	9.275646657	9.1662	58.84	64.23	glyS	Glycyl-tRNA synthetase (EC 6.1.1.14) (Glycine–tRNA ligase) (GlyRS)
MPN355	10.4	11.1	8.1279	8.115105184	8.1823	13.45	15.06	yacO	Probable 23S rRNA (guanosine-2'-O-)-methyltransferase rlmB (EC 2.1.1.-)
MPN356	10.9	10.3	8.4014	7.337627939	7.4005	10.16	7.43	cysS	Cysteinyl-tRNA synthetase (EC 6.1.1.16) (Cysteine–tRNA ligase) (CysRS)
MPN357	11.5	9.5	8.9933	6.629779192	7.5627	21.3	21.45	lig	DNA ligase (EC 6.5.1.2) (Polydeoxyribonucleotide synthase [NAD+])
MPN358	10.3	11.0	6.7939	7.402732218	8.45	46.13	41.61		Uncharacterized protein MPN_358
MPN359	8.7	10.0	8.5322	10.33369599	9.54	31.35	35.65		Conserved hypothetical protein MPN_359
MPN360	13.3	13.8	9.9219	10.77235823	9.43	40.8	39.76	rpmE	50S ribosomal protein L31
MPN361	10.6	9.4	8.4331	8.844934584	7.63	24.47	23.52	prfA	Peptide chain release factor 1 (RF-1)

Continued on next page

Table S1 – Transcriptome and Proteome data – continued from previous page									
ORF	RNA micro-arrays 6h	RNA micro-array 96h	RNAseq 6h	RNAseq 96h	Tiling 37 average expression level	Protein copy number per cell 6h	Protein copy number per cell 96h	Protein name	Function
MPN362	10.6	11.4	5.3077	5.162849615	6.73	7.43	7.1	hemK	Protein methyltransferase hemK (EC 2.1.1.-) (Protein-glutamine N-methyltransferase hemK) (Protein-(glutamine-N(5)) MTase hemK)
MPN363	11.2	12.6	7.4273	7.431222121	8.83	9.78	10.65		Conserved hypothetical lipoprotein MPN_363
MPN364	8.4	8.7	3.7373	4.143227472	6.39	19.58	16.92		Conserved hypothetical protein MPN_364
MPN365	-Inf	12.1	5.8848	6.305723759	8.13	2.73	0		Putative type-1 restriction enzyme specificity protein MPN_365 (S.mpnORFCP) (Type I restriction enzyme specificity protein MPN_365) (S protein)
MPN366	13.2	13.3	6.2560	6.479766381	9.41	59.77	42.27		Putative mgpC-like protein MPN_366
MPN367	14.1	13.5	5.8598	6.400861489	10.2	59.32	50.76		Putative mgpC-like protein MPN_367
MPN368	10.0	10.6	7.7625	8.479252876	7.57	14.71	5.33		Uncharacterized protein MPN_368
MPN369	10.7	11.4	9.5656	9.816714646	7.7172	40.03	0		Uncharacterized lipoprotein MPN_369
MPN370	8.4	8.8	4.0743	4.496504566	7.94	219.28	198.7		Putative adhesin P1-like protein MPN_370
MPN371	9.8	10.9	4.2271	4.154731503	7.1591	0	0		Uncharacterized protein MPN_371
MPN372	12.6	10.8	7.1985	6.060665323	8.45	159.46	156.56	ptxA	ADP-ribosylating toxin CARDS (EC 2.4.2.-) (CARDX TX) (ADP-ribosyltransferase CARDS)
MPN373	9.4	10.1	8.3388	8.300329416	7.8128	0	0		Uncharacterized protein MPN_373
MPN374	7.8	8.1	3.6943	5.484332787	5.8542	0	0		Uncharacterized protein MPN_374
MPN375	10.5	10.7	9.0449	6.843123748	8.1331	0	0		Uncharacterized protein MPN_375
MPN376	12.0	13.9	14.3658	14.38426001	11.739	131.22	227.52		Uncharacterized protein MPN_376
MPN377	12.4	14.1	10.3633	12.12925651	11.1	576.82	525.35		Uncharacterized protein MPN_377
MPN378	10.1	9.8	6.5954	6.680968726	8.09	35.75	35.21	dnaE	DNA polymerase III subunit alpha (EC 2.7.7.7)
MPN379	10.7	9.8	7.7099	7.963204776	7.7	29	27.62	polA	5'-3' exonuclease (EC 3.1.11.-)
MPN380	10.0	10.1	5.8731	6.289939983	7.22	31.76	34.06	fpg	Formamidopyrimidine-DNA glycosylase (Fapy-DNA glycosylase) (EC 3.2.2.23) (DNA-(apurinic or apyrimidinic site) lyase mutM) (AP lyase mutM) (EC 4.2.99.18)
MPN381	10.9	11.0	7.3642	7.768973821	8.82	84.01	91.31	yidA	Putative Cof-like hydrolase acting on phosphorylated sugars and small metabolites
MPN382	9.2	10.1	5.7352	6.143801203	7.86	0	2.17	coaE	Dephospho-CoA kinase (EC 2.7.1.24) (Dephosphocoenzyme A kinase)
MPN383	10.5	11.2	10.9076	10.65005685	9.5309	102.58	71.48	yidA	nucleotidase, also common to HADs (works well also with CoA and NADP)
MPN384	8.9	9.2	10.0650	8.437076393	9.4365	109.06	82.6	leuS	Leucyl-tRNA synthetase (EC 6.1.1.4) (Leucine–tRNA ligase) (LeuRS)
MPN385	11.2	11.8	10.7278	10.98860732	9.2234	0	9.9		Conserved hypothetical protein MPN_385
MPN386	12.9	14.4	13.2458	13.57284802	10.429	333.18	345.29	yaaF	Putative deoxynucleoside kinase protein (EC 2.7.1.76, EC 2.7.1.113)
MPN387	10.7	9.7	10.5529	9.414619801	9.2945	60.56	68.99		Conserved hypothetical protein MPN_387
MPN388	11.1	9.6	11.9680	11.85047352	9.5581	54.26	24.1		Conserved hypothetical protein MPN_388
MPN389	13.3	11.6	11.8193	11.20188118	10.055	521.01	539.55	lplA	Probable lipoate-protein ligase A (Lipoate–protein ligase) (EC 2.7.7.63)
MPN390	13.2	12.2	11.7245	11.72294543	10.256	736.37	651.63	pdhD	Dihydrolipoyl dehydrogenase (EC 1.8.1.4) (Dihydrolipoamide dehydrogenase) (E3 component of pyruvate complex)
MPN391	14.3	13.6	11.3070	11.35414511	10.798	740.46	755.66	pdhC	Dihydrolipoyllysine-residue acetyltransferase component of pyruvate dehydrogenase complex (EC 2.3.1.12) (E2) (Dihydrolipoamide acetyltransferase component of pyruvate dehydrogenase complex)
MPN392	13.0	13.8	12.7957	12.78906924	11.797	2017.58	2077.91	pdhB	Pyruvate dehydrogenase E1 component subunit beta (EC 1.2.4.1)
MPN393	13.8	14.2	12.9746	13.10993571	11.614	2228.58	2269.69	pdhA	Pyruvate dehydrogenase E1 component subunit alpha (EC 1.2.4.1)
MPN394	12.1	13.1	11.7369	12.1385719	11.384	1271.12	1095.2	nox	Probable NADH oxidase (NOXase) (EC 1.6.99.3)
MPN395	11.4	10.6	11.1015	10.42744475	9.4092	50.48	49.25	apt	Adenine phosphoribosyltransferase (APRT) (EC 2.4.2.7)
MPN396	11.6	11.2	11.4094	10.59218982	9.3335	48.32	46	secD	Protein-export membrane protein secD
MPN397	8.8	9.4	5.2695	7.256077353	8.01	44.1	50.36	spoT	Probable guanosine-3',5'-bis(diphosphate) 3'-pyrophosphohydrolase (EC 3.1.7.2) (Penta-phosphate guanosine-3'-pyrophosphohydrolase) ((ppGpp)ase)
MPN398	12.2	12.0	12.1043	11.98650881	9.3771	60.31	65.49		Uncharacterized protein MPN_398
MPN399	14.6	14.0	12.8801	12.32454519	10.105	29.33	21.93		Conserved hypothetical protein MPN_399
MPN400	15.1	15.7	14.5366	14.42527765	11.382	56.31	23.52		Conserved hypothetical protein MPN_400
MPN401	12.7	13.4	10.6627	11.07849647	10.109	319.8	321.92	greA	Transcription elongation factor greA (Transcript cleavage factor greA)
MPN402	10.1	9.6	6.8774	6.430852241	7.92	39.71	33.3	proS	Prolyl-tRNA synthetase (EC 6.1.1.15) (Proline–tRNA ligase) (ProRS)
MPN403	10.4	12.9	7.9280	8.633192743	8.63	0	0		Conserved hypothetical protein MPN_403
MPN404	8.1	10.2	5.4119	6.554322124	8.16	0	0		Conserved hypothetical protein MPN_404
MPN405	8.2	9.0	4.5280	5.95272412	7.72	0	0		Conserved hypothetical protein MPN_405
MPN406	10.4	13.5	10.6856	10.86486861	8.64	5.65	15.49	acpA	Acyl carrier protein homolog
MPN407	9.5	11.2	9.1091	9.102839404	8.9523	8.55	14.24		Predicted lipase
MPN408	8.4	7.8	5.7730	4.769088325	7.32	37.81	31.54		Conserved hypothetical lipoprotein MPN_408
MPN409	7.4	6.9	4.0337	4.298243384	8.14	18.46	10.48		Putative adhesin P1-like protein MPN_409
MPN410	13.3	14.8	8.2632	9.098989009	8.92	53.73	16.14		Uncharacterized protein MPN_410

Continued on next page

Table S1 – Transcriptome and Proteome data – continued from previous page

ORF	RNA micro-arrays 6h	RNA micro-array 96h	RNAseq 6h	RNAseq 96h	Tiling 37 average expression level	Protein copy number per cell 6h	Protein copy number per cell 96h	Protein name	Function
MPN411	5.8	8.6	8.7341	8.580727476	6.5504	0.3	0		Conserved hypothetical lipoprotein MPN_411
MPN412	7.7	7.3	3.9692	5.122617701	8.01	120.78	130.65		Uncharacterized protein MPN_412
MPN413	13.3	13.1	0.9066	2.774810995	0	0	0		Uncharacterized protein MPN_413
MPN414	12.8	12.5	3.2915	3.018594469	8.38	63.69	49.01		Putative mgpC-like protein MPN_414
MPN415	8.0	9.3	6.1259	7.10419645	7.64	15.97	16.97	hatA	High affinity transport system protein p37
MPN416	8.8	8.2	5.5722	5.325682192	7.11	4.19	3.02	hatB	Probable ABC transporter ATP-binding protein p29
MPN417	8.4	9.9	4.1451	4.373754138	6.73	0	0	hatC	ABC transport system permease protein p69
MPN418	9.1	8.2	7.0599	5.954294543	7.9404	46.11	41.23	alaS	Alanyl-tRNA synthetase (EC 6.1.1.7) (Alanine–tRNA ligase) (AlaRS)
MPN419	10.4	9.7	9.6024	8.946051982	8.8323	71.78	76.66	ruvX	Putative Holliday junction resolvase (EC 3.1.-.-)
MPN420	11.4	12.5	11.1031	10.79525498	10.269	68.7	64.47	glpQ	Glycerophosphoryl diester phosphodiesterase
MPN421	10.8	12.6	11.4895	12.23087121	10.189	28.13	16.77	cglT	Major facilitator family protein CglT
MPN422	8.4	8.6	8.0982	7.209244327	8.7852	25.12	24.98	mnmA	tRNA-specific 2-thiouridylase mnmA (EC 2.8.1.-)
MPN423	9.8	11.6	8.7477	9.15000199	9.214	20.07	23.42		Conserved hypothetical protein MPN_423
MPN424	10.6	10.5	6.7749	6.65196054	8.108	0	1.42	ylxM	Putative helix-turn-helix protein, YlxM/p13-like protein; Transcription factor.
MPN425	9.7	10.8	8.9568	8.578165683	8.3495	55.25	52.9	ftsY	Cell division protein ftsY homolog
MPN426	9.2	9.0	8.7813	8.246895438	8.3992	47.32	44.47	smc	SMC family, chromosome/DNA binding/protecting functions
MPN427	9.7	12.5	7.2918	8.372548544	10.2	101.12	108.26	yidA	P-sugar phosphatase YidA (Canadian consortium)
MPN428	13.1	14.3	11.8522	11.4177094	11.209	552.01	515.91	pta	Phosphate acetyltransferase (EC 2.3.1.8) (Phosphotransacetylase)
MPN429	10.8	12.3	10.4579	10.62135883	11.026	604.15	513.68	pgk	Phosphoglycerate kinase (EC 2.7.2.3)
MPN430	9.9	12.3	12.7924	13.10390256	11.72	2503.62	2644.96	gap	Glyceraldehyde-3-phosphate dehydrogenase (GAPDH) (EC 1.2.1.12)
MPN431	7.6	6.5	7.9704	6.40166967	7.4386	5.72	7.15	cbiQ	Similar to CbiQ, cobalt ABC transporter ATP-binding protein
MPN432	10.1	9.8	10.7474	11.168127	8.7141	7.58	8.41	cbiO 1	Putative cobalt ABC transporter ATP-binding protein
MPN433	9.2	9.4	8.1271	6.455465238	7.5993	9.44	46.29	cbiO 2	Putative cobalt ABC transporter ATP-binding protein CbiO
MPN434	13.9	14.6	12.5145	13.27757197	11.906	2197.63	2293.61	dnaK	Chaperone protein DnaK (Heat shock protein 70) (Heat shock 70 kDa protein) (HSP70)
MPN435	10.2	10.1	9.2929	8.744411764	8.4209	0	12.61		Uncharacterized permease
MPN436	11.1	10.6	10.0146	9.914815063	8.4387	32.82	26.93		Uncharacterized lipoprotein MPN_436
MPN437	9.1	10.1	7.4593	7.691550718	6.3935	0	0		Uncharacterized protein MPN_437
MPN438	7.9	9.6	6.7694	6.614002022	6.5079	0	0		Uncharacterized protein MPN_438
MPN439	8.5	9.0	7.0260	6.963534751	6.6207	0	0		Uncharacterized lipoprotein MPN_439
MPN440	6.6	6.1	7.3594	6.383480573	6.3179	15.49	0.89		Uncharacterized protein MPN_440
MPN441	0.0	0.0	NAME?	NAME?	5.8	0	0		Conserved hypothetical protein MPN_441
MPN442	10.4	9.0	8.9404	6.834324919	8.0826	0	0		Uncharacterized lipoprotein MPN_442
MPN443	10.8	9.7	9.8963	8.293231413	8.7066	6.47	21.7		Probable ATP-dependent RNA helicase (EC 3.6.1.-)
MPN444	8.8	9.1	10.9617	10.36877183	9.1346	26.7	12.11		Conserved hypothetical lipoprotein MPN_444
MPN445	12.3	12.2	9.2041	9.330774898	9.54	46.84	44.45	lip3	Triacylglycerol lipase (lip) 3 (EC 3.1.-.-)
MPN446	13.4	13.2	13.9940	14.0757868	10.886	114.09	96.29	rpsD	30S ribosomal protein S4
MPN447	11.6	12.0	11.7591	11.15597452	10.061	168.04	200.39	HWM1	Cytadherence high molecular weight protein 1 (Cytadherence accessory protein 1)
MPN448	9.8	10.2	9.6918	8.560992036	7.4278	0	0	fbp	Major ferric iron-binding protein
MPN449	11.3	11.6	11.5308	10.63127286	9.0883	26.01	8.72		Conserved hypothetical protein MPN_449
MPN450	8.9	11.6	10.8908	11.38724989	9.2364	14.75	12.53	holA	DNA polymerase III subunit delta (EC 2.7.7.7)
MPN451	7.4	7.1	10.1230	9.928537701	6.763	0	0	comE3	ComE operon protein 3 related protein
MPN452	11.5	10.8	11.7415	9.779989459	10.03	241.71	209.54	hmw3	Cytadherence high molecular weight protein 3 (Cytadherence accessory protein 3) (Accessory adhesin protein 3)
MPN453	12.6	13.2	10.3531	9.495775937	10.771	46.95	46.92	P3	P30 adhesin (Cytadhesin P30) (30 kDa adhesin-related protein)
MPN454	12.9	14.3	11.1907	11.11458161	11.117	77.64	95.78		Conserved hypothetical protein MPN_454
MPN455	13.0	13.5	11.1410	11.63659957	10.224	57.26	10.82	ctaD	Putative phosphatidic acid phosphatase family protein
MPN456	12.3	11.0	8.8981	6.99413521	7.6894	82.75	84.05		Conserved hypothetical lipoprotein MPN_456
MPN457	9.4	8.3	-0.9971	4.497202676	6.5269	53.92	64.11		Uncharacterized protein MPN_457
MPN458	11.3	9.2	8.5311	5.916935642	7.0119	55.34	65.75		Conserved hypothetical protein MPN_458
MPN459	12.1	8.5	9.0832	7.094513017	7.6138	35.3	40.55		Conserved hypothetical lipoprotein MPN_459
MPN460	10.5	9.7	8.7032	8.755050129	8.8052	11.12	0	ktrB	Ktr system potassium uptake protein B
MPN461	10.6	10.3	6.1048	6.90295566	9.03	40.6	42.79	ktrA	Ktr system potassium uptake protein A
MPN462	9.5	8.6	3.6353	3.957315943	8.46	53.82	96.87		Uncharacterized protein MPN_462
MPN463	10.0	10.5	4.6414	4.869506796	9.06	0	0		Conserved hypothetical protein MPN_463
MPN464	10.0	10.1	4.0596	4.175326644	8.25	44.2	43.01		Putative mgpC-like protein MPN_464
MPN465	11.3	12.7	7.7008	6.225356945	6.6591	0	0		Conserved hypothetical protein MPN_465
MPN466	9.1	9.1	10.7721	10.44062779	7.0813	5.48	5.29		Conserved hypothetical protein MPN_466

Continued on next page

Table S1 – Transcriptome and Proteome data – continued from previous page

ORF	RNA micro-arrays 6h	RNA micro-array 96h	RNAseq 6h	RNAseq 96h	Tiling 37 average expression level	Protein copy number per cell 6h	Protein copy number per cell 96h	Protein name	Function
MPN467	12.5	12.7	9.2279	8.945502339	7.8977	40.03	0		Conserved hypothetical lipoprotein MPN_467
MPN468	10.1	10.4	3.1661	4.626611126	7.45	136.58	71.29		Putative adhesin P1-like protein MPN_468
MPN469	8.9	7.0	10.6086	8.867377315	8.2948	13.49	8.72		Conserved hypothetical protein MPN_469
MPN470	11.2	10.6	11.8748	11.63546094	10.163	431	438.32	pepX	Putative Xaa-Pro aminopeptidase (X-Pro aminopeptidase) (EC 3.4.11.9) (Aminopeptidase P) (APP) (Aminoacylproline aminopeptidase)
MPN471	12.7	13.7	13.7655	13.99363447	10.69	20.56	11.32	rpmG	50S ribosomal protein L33 1
MPN472	12.6	14.3	10.2201	10.71643408	9.4067	37.85	31.95	degV	DegV family protein
MPN473	9.7	11.0	8.8642	9.153605249	9.0668	7.9	5.67	lip2	Putative esterase/lipase 2 (EC 3.1.-.-)
MPN474	12.4	13.2	11.2156	11.36711653	10.4	496.82	461.72		Uncharacterized protein MPN_474
MPN475	9.1	8.7	7.7050	7.587486216	8.0106	13.47	16.51	engA	GTP-binding protein engA
MPN476	7.6	8.2	8.6584	7.452238379	9.2754	36.2	41.25	cmk	Cytidylate kinase (CK) (EC 2.7.4.14) (Cytidine monophosphate kinase) (CMP kinase)
MPN477	10.4	10.6	10.8449	9.034790133	9.376	9.01	7.98		Conserved hypothetical protein MPN_477
MPN478	9.5	9.6	13.1432	13.36847455	10.271	50.29	46.79	YebC	YebC family protein; Transcription factor of the tetR family
MPN479	11.6	12.7	11.2186	11.20692273	10.826	466.06	451.84	acpH	Acyl carrier protein phosphodiesterase (ACP phosphodiesterase) (EC 3.1.4.14)
MPN480	9.9	8.3	7.9353	6.118348466	8.1997	25.16	30.15	valS	Valyl-tRNA synthetase (EC 6.1.1.9) (Valine–tRNA ligase) (ValRS)
MPN481	6.0	7.9	10.3611	7.826324728	8.8179	15.22	14.51	engB	Probable GTP-binding protein engB
MPN482	12.3	12.4	10.9253	10.96012636	10.135	3.04	2.77		Uncharacterized protein MPN_482
MPN483	10.6	8.8	6.1792	7.386260853	7.95	20.24	22.68	yibD	Glycosyltransferase (EC 2.4.1.157, EC 2.4.1.46)
MPN484	6.5	7.5	9.1967	8.684736244	7.5761	22.59	9.4		Uncharacterized protein MPN_484
MPN485	11.6	11.3	8.5766	7.936911725	7.6589	10.95	7.3		Uncharacterized protein MPN_485
MPN486	7.3	8.5	NAME?	0.787094852	6.31	0	0		Uncharacterized protein MPN_486
MPN487	9.1	7.2	4.5305	5.144163615	7.77	26.97	26.58	nifS	Probable cysteine desulfurase (EC 2.8.1.7)
MPN488	8.8	7.0	6.2293	6.629726599	7.93	15.46	23.17	nifU	NifU-like protein
MPN489	9.3	8.0	10.1562	9.526790056	9.4325	26.19	12.6		Uncharacterized lipoprotein MPN_489
MPN490	8.6	9.5	10.0344	9.908580874	8.9376	3.93	16.33	recA	Protein recA (Recombinase A)
MPN491	14.0	13.6	9.2966	10.43026999	10.9	66.07	79.2	mnuA	Membrane nuclease A
MPN492	8.3	8.2	6.3599	6.333971675	7.9604	10.63	12.85	ulaE	Probable L-ribulose-5-phosphate 3-epimerase ulaE (EC 5.1.3.22) (L-xylulose-5-phosphate 3-epimerase) (L-ascorbate utilization protein E)
MPN493	8.9	8.2	6.0177	5.887787667	8.3919	13.72	11.01	ulaD	Probable 3-keto-L-gulonate-6-phosphate decarboxylase (KGPDC) (EC 4.1.1.85) (3-dehydro-L-gulonate-6-phosphate decarboxylase) (L-ascorbate utilization protein D)
MPN494	10.2	11.7	9.5531	9.032080893	9.158	35.37	26.95	ulaC	Ascorbate-specific phosphotransferase enzyme IIA component (EC 2.7.1.-) (PTS system ascorbate-specific EIIA component)
MPN495	9.7	12.6	9.8778	10.31033016	9.1644	35.54	30.26	ulaB	Ascorbate-specific phosphotransferase enzyme IIB component (EC 2.7.1.69) (Ascorbate-specific PTS system EIIB component)
MPN496	8.3	9.2	7.1613	7.596720629	8.0019	13.88	1.86	ulaA	Ascorbate-specific permease IIC component ulaA (Ascorbate-specific PTS system EIIC component)
MPN497	11.6	13.7	11.5128	12.41884071	11.173	0	0	ulaG	Probable L-ascorbate-6-phosphate lactonase ulaG (EC 3.1.1.-)
MPN498	10.6	12.9	6.7628	8.556989124	10.3	24.77	26.94	araD	Probable L-ribulose-5-phosphate 4-epimerase ulaF (EC 5.1.3.4) (Phosphoribulose isomerase) (L-ascorbate utilization protein F)
MPN499	8.8	11.1	6.2134	7.656057312	9.45	13.24	10.77		Uncharacterized protein MPN_499
MPN500	11.8	13.3	3.7723	4.480851253	8.75	63.2	69.53		Putative adhesin P1-like protein MPN_500
MPN501	12.4	12.9	7.9701	9.443330122	9.48	44.56	16.53		Uncharacterized protein MPN_501
MPN502	12.1	13.2	3.5434	4.687400781	9.03	195.92	174.84		Uncharacterized protein MPN_502
MPN503	10.1	11.1	0.3408	1.739053952	8	2.77	5.88		Putative mgpC-like protein MPN_503
MPN504	10.3	11.9	7.0324	8.230194393	8.66	4.54	13.3		Uncharacterized protein MPN_504
MPN505	9.0	11.7	9.5273	10.34681822	8.2244	18.99	9.44		Uncharacterized protein MPN_505
MPN506	11.3	9.3	5.9550	6.893076425	7.38	32.43	51.42		Conserved hypothetical lipoprotein MPN_506
MPN507	9.9	9.3	5.5489	5.789533713	6.29	13.98	1.45		Putative type-1 restriction enzyme specificity protein MPN_507 (S.mpnORFBP) (Type I restriction enzyme specificity protein MPN_507) (S protein)
MPN508	9.7	10.0	9.8613	9.776509623	8.1178	0	0		Putative membrane export protein
MPN509	11.5	11.3	10.7314	9.998152936	8.5561	9.06	2.51		Uncharacterized protein MPN_509
MPN510	9.1	7.5	8.4778	6.996048409	6.9618	0	0		Uncharacterized protein MPN_510
MPN511	7.1	5.8	11.4968	11.47581691	8.1474	0	0		Uncharacterized protein MPN_511
MPN512	11.7	11.6	10.3162	9.379387743	9.3045	7.36	0		Uncharacterized protein MPN_512
MPN513	8.1	7.1	5.3805	2.456925423	6.0047	0	0		Conserved hypothetical protein MPN_513
MPN514	9.0	9.2	7.4415	6.784779081	8.0371	0	0		Conserved hypothetical protein MPN_514
MPN515	13.4	10.4	10.2547	8.218960456	9.1925	220.99	231.33	rpoC	DNA-directed RNA polymerase subunit beta' (RNAP subunit beta') (EC 2.7.7.6) (Transcriptase subunit beta') (RNA polymerase subunit beta')

Continued on next page

Table S1 – Transcriptome and Proteome data – continued from previous page									
ORF	RNA micro-arrays 6h	RNA micro-array 96h	RNAseq 6h	RNAseq 96h	Tiling 37 average expression level	Protein copy number per cell 6h	Protein copy number per cell 96h	Protein name	Function
MPN516	13.3	9.8	10.2742	7.925500936	8.5437	285.85	330.15	rpoB	DNA-directed RNA polymerase subunit beta (RNAP subunit beta) (EC 2.7.7.6) (Transcriptase subunit beta) (RNA polymerase subunit beta)
MPN517	10.2	13.6	10.2514	11.49555615	10.25	253.68	279.25	yhdA	Putative NADPH-dependent FMN reductase
MPN518	10.3	12.4	10.5896	12.08828373	9.5991	162.23	172.32		Conserved hypothetical protein MPN_518
MPN519	8.5	8.9	9.0828	8.91800833	7.5597	15.13	14.92	lip3	Putative esterase/lipase 3 (EC 3.1.-.-)
MPN520	10.6	11.0	9.5991	9.495958563	8.8962	66.46	68.52	ileS	Isoleucyl-tRNA synthetase (EC 6.1.1.5) (Isoleucine–tRNA ligase) (IleRS)
MPN521	9.9	10.6	4.8615	7.265973951	7.9	22.06	17.4	ygl3	Probable tRNA/rRNA methyltransferase SpoU (EC 2.1.1.-)
MPN522	9.4	9.5	6.2631	5.68758888	7.39	13.14	8.74	trmB	tRNA (guanine-N(7)-)-methyltransferase (EC 2.1.1.33) (tRNA(m7G46)-methyltransferase)
MPN523	12.7	14.0	12.5644	12.8637981	10.969	50.63	21.04		Uncharacterized lipoprotein MPN_523
MPN524	12.2	13.6	10.3885	10.51483547	8.1511	70.82	19.63		Uncharacterized protein MPN_524
MPN525	10.0	11.2	6.7651	8.185200435	7.58	0	0	dnaB	Replication initiation and membrane attachment protein
MPN526	10.4	10.8	6.1010	7.224432027	7.29	32.77	33.72		Conserved hypothetical protein MPN_526
MPN527	9.3	10.5	9.4093	10.70679496	7.8816	0	0		Uncharacterized protein MPN_527
MPN528	11.1	11.9	6.4867	8.320341231	9.36	193.49	181.37	ppa	Inorganic pyrophosphatase (EC 3.6.1.1) (Pyrophosphate phospho-hydrolase) (PPase)
MPN529	12.1	11.9	11.0574	11.02897537	9.0712	67.64	54.51	ihf	Histone-like bacterial DNA-binding protein
MPN530	12.7	13.7	9.7146	11.36195807	9.93	511.99	495.83		Conserved hypothetical protein MPN_530
MPN531	10.5	12.1	9.9536	10.2927274	9.8763	164.31	234.46	clpB	Chaperone protein clpB; ClpB is a chaperon but is not a protease, to function as a protease it needs to make a complex with another protein. It is called ClpC in subtilis and the hexameric protease is called ClpP
MPN532	11.1	11.4	11.4921	10.38930937	9.3129	39.94	53.46	licA	Predicted choline kinase (EC 2.7.1.32)
MPN533	12.5	12.7	11.8036	12.03816448	10.682	917.76	1001.36	ackA	Acetate kinase (EC 2.7.2.1) (Acetokinase)
MPN534	8.6	8.6	5.2913	5.669907201	7.36	0	0		Conserved hypothetical protein MPN_534
MPN535	9.3	9.1	4.9262	4.928572472	7.79	0	0	ruvA	Holliday junction ATP-dependent DNA helicase ruvA (EC 3.6.1.-)
MPN536	8.6	7.0	2.6572	2.214125945	7.75	0	0	ruvB	Holliday junction ATP-dependent DNA helicase ruvB (EC 3.6.1.-)
MPN537	10.6	9.8	9.1658	9.356102001	8.0587	0	0	mucB	UV protection protein MucB
MPN538	14.8	12.1	8.3874	7.379315944	9.1	49.02	55.41	rplJ	50S ribosomal protein L10
MPN539	14.6	11.2	8.4141	7.77592838	9.02	232.77	256.42	rplL	50S ribosomal protein L7/L12
MPN540	14.1	10.4	7.5755	6.092594953	8.73	37.36	25.55	rpmF	50S ribosomal protein L32
MPN541	11.1	11.8	11.4092	11.7313086	8.4452	90.78	59.29	rpsT	30S ribosomal protein S20
MPN542	11.2	12.0	7.4881	7.895044432	8.92	9.86	9.85		Uncharacterized protein MPN_542
MPN543	9.8	8.9	5.0118	5.316296254	7.15	8.17	5.79	fmt	Methionyl-tRNA formyltransferase (EC 2.1.2.9)
MPN544	9.4	9.3	7.2914	8.780669385	6.49	6.97	6.16		Conserved hypothetical protein MPN_544
MPN545	10.0	10.1	9.0934	9.009985848	7.481	41.25	36.72	rnc	Ribonuclease 3 (EC 3.1.26.3) (Ribonuclease III) (RNase III)
MPN546	9.8	9.8	10.2239	9.64148941	8.6606	38.77	35.99	plsX	Fatty acid/phospholipid synthesis protein plsX (EC 2.3.1.15)
MPN547	9.0	9.3	9.2973	8.66223707	7.7627	91.54	81.97	dak	Dihydroxyacetone kinase (DHA kinase) (EC 2.7.1.29) (Glycerone kinase)
MPN548	8.4	9.1	6.2148	6.520548865	7.3252	9.63	5.95	truC	RNA pseudouridine synthase TruC (EC 5.4.99.-) (RNA-uridine isomerase) (RNA pseudouridylate synthase)
MPN549	10.3	10.1	8.0777	7.054387783	8.1703	37.02	45.25	recJ	Probable single-stranded-DNA-specific exonuclease recJ (EC 3.1.-.-)
MPN550	9.6	9.5	9.2964	9.647375866	7.9254	130.3	8.78	thiI	Probable thiamine biosynthesis protein thiI or tRNA sulfur transferase modification enzyme (EC 2.8.1.4)
MPN551	9.5	8.3	8.3984	7.09653333	7.0945	7.76	7.64	yqaJ	Uncharacterized protein yqaJ
MPN552	7.5	6.4	8.7341	7.701759495	7.1631	11.25	10.55		Conserved hypothetical protein MPN_552
MPN553	10.6	8.8	9.4050	7.898133332	8.2127	63.45	74.17		Threonyl-tRNA synthetase (EC 6.1.1.3) (Threonine–tRNA ligase) (ThrRS)
MPN554	11.7	11.5	11.1366	10.55233925	9.1992	13.49	20.92	ywpH	Putative single-stranded DNA-binding protein (SSB) (SsbB) involved in genetic recombination during normal transformation
MPN555	13.4	12.9	11.3697	11.82182407	10.656	1073.93	1055.13		DNA/RNA binding
MPN556	11.5	11.6	10.9538	11.25284897	9.8053	146.7	139.02	argS	Arginyl-tRNA synthetase (EC 6.1.1.19) (Arginine–tRNA ligase) (ArgRS)
MPN557	10.1	9.6	6.0569	7.002516355	8.25	28.24	27.59	gidA	tRNA uridine 5-carboxymethylaminomethyl modification enzyme mnmG (Glucose-inhibited division protein A)
MPN558	9.4	8.7	6.3514	6.055610374	7.9	23.47	21.99	gidB	Ribosomal RNA small subunit methyltransferase G (EC 2.1.1.-) (16S rRNA 7-methylguanosine methyltransferase) (16S rRNA m7G methyltransferase) (Glucose-inhibited division protein B)
MPN559	10.0	8.8	5.2969	4.688977493	7.32	3.04	5.85		Conserved hypothetical protein MPN_559
MPN560	9.2	9.4	10.9873	11.06778554	9.9208	83	91.45	arcA	Arginine deiminase-like protein

Continued on next page

Table S1 – Transcriptome and Proteome data – continued from previous page									
ORF	RNA micro-arrays 6h	RNA micro-array 96h	RNAseq 6h	RNAseq 96h	Tiling 37 average expression level	Protein copy number per cell 6h	Protein copy number per cell 96h	Protein name	Function
MPN561	6.8	7.7	5.3955	6.503346771	8.28	18.29	24.75	udk	Uridine kinase (EC 2.7.1.48) (Uridine monophosphokinase) (Cytidine monophosphokinase)
MPN562	8.6	7.6	8.2227	7.756827004	8.8064	40.65	38.71	outB	Probable NH(3)-dependent NAD(+) synthetase (EC 6.3.1.5)
MPN563	9.8	9.8	8.5809	8.789940118	9.1881	23.55	22.93	obg	GTP-binding protein Obg; Involved in stringent response
MPN564	10.0	11.4	11.1329	12.14953752	11.427	17.33	38.32	adh	Probable NADP-dependent alcohol dehydrogenase (EC 1.1.1.2)
MPN565	8.7	9.1	8.3724	5.598861251	6.7414	0	0		Conserved hypothetical protein MPN_565
MPN566	11.2	10.5	9.8974	9.354282528	8.4041	48.54	42.94	glpQ	Glycerophosphoryl diester phosphodiesterase. [EC:3.1.4.46]
MPN567	10.9	10.5	10.1375	9.836410795	8.6206	54.4	31.95	p2	Protein P200
MPN568	7.7	7.2	9.2443	8.812668884	7.1286	14.93	14.5	spg	GTP-binding protein Era homolog
MPN569	8.0	8.1	9.7125	9.70401232	7.4817	10.07	4.96	ybeY	Putative metalloprotease MPN_569 (EC 3.4.24.-)
MPN570	6.9	7.9	10.5676	11.43747112	6.4675	0	0		Conserved hypothetical proteinMPN_570
MPN571	6.5	4.8	5.8517	4.374989212	6.0773	0	0		Hemolysin-type ABC transporter
MPN572	14.3	14.1	11.9942	12.14718877	11.378	1146.1	1066.33	pepA	Probable cytosol aminopeptidase (EC 3.4.11.1) (Leucine aminopeptidase) (LAP) (Leucyl aminopeptidase); DNA binding protein
MPN573	10.9	11.4	10.5699	11.24329581	10.948	1284.93	1359.75	groEL	60 kDa chaperonin (Protein Cpn60) (GroEL protein)
MPN574	13.1	13.0	10.4366	11.50701535	10.31	978.79	1103.42	groES	10 kDa chaperonin (Protein Cpn10) (GroES protein)
MPN575	10.2	9.0	7.9904	6.474341147	7.2206	0	0		Conserved hypothetical protein MPN_575
MPN576	12.3	12.0	9.9341	10.03951885	9.9458	288.85	305.24	glyA	Serine hydroxymethyltransferase (Serine methylase) (SHMT) (EC 2.1.2.1)
MPN577	8.0	8.0	7.1720	5.264755328	7.3666	0	0		Conserved hypothetical protein MPN_577
MPN578	6.0	6.9	3.2635	2.958435469	6.03	0	0		Conserved hypothetical protein MPN_578
MPN579	10.3	11.3	9.6399	9.668651101	8.2162	0	0		Conserved hypothetical protein MPN_579
MPN580	8.7	8.1	6.8015	5.10620228	6.5773	0	0		Putative protease MPN_580
MPN581	8.3	6.4	11.4691	11.15146742	8.4512	0	0		Conserved hypothetical protein MPN_581
MPN582	9.3	9.7	8.2823	8.213140687	7.1578	0	0		Conserved hypothetical lipoprotein MPN_582
MPN583	6.2	5.3	6.9205	7.205040462	7.4028	0	0		Conserved hypothetical protein MPN_583
MPN584	7.8	7.4	8.4112	8.081974641	7.1927	0	0		Conserved hypothetical protein MPN_584
MPN585	6.7	6.0	9.6496	9.463321551	7.4213	0	0		Conserved hypothetical lipoprotein MPN_585
MPN586	6.3	7.7	8.2710	8.893783499	7.505	0	0		Conserved hypothetical protein MPN_586
MPN587	7.9	9.7	9.4681	9.507058481	8.6721	0	0		Conserved hypothetical lipoprotein MPN_587
MPN588	9.8	11.6	11.1805	12.06008306	9.2089	0	0.77		Uncharacterized lipoprotein MPN_588
MPN589	6.4	7.4	8.4550	7.532277855	6.9877	0	0		Conserved hypothetical protein MPN_589
MPN590	11.0	12.6	8.8261	5.997870478	8.0026	14.83	23.46		Conserved hypothetical lipoprotein MPN_590
MPN591	11.1	12.7	11.2805	11.07644168	10.126	29.13	32.71		Conserved hypothetical protein MPN_591
MPN592	13.1	14.1	13.3411	13.68362057	10.778	20.74	25.2		Conserved hypothetical lipoprotein MPN_592
MPN593	11.9	12.6	10.9875	11.10745768	9.1543	13.33	0		Conserved hypothetical protein MPN_593
MPN594	7.7	8.0	9.2722	9.950339447	8.6155	0	0		Conserved hypothetical protein MPN_594
MPN595	7.9	9.3	9.4017	9.650136404	8.8176	43.95	33.83	lacA	Probable ribose-5-phosphate isomerase B (EC 5.3.1.6) (Phosphoriboisomerase B)
MPN596	8.7	9.6	9.8784	9.7729173	8.0077	6.05	5.43	erzA	Negative regulator of FtsZ ring formation
MPN597	10.9	10.6	11.4139	10.35787463	8.8896	28.45	28.36	atpC	ATP synthase epsilon chain (ATP synthase F1 sector epsilon subunit) (F-ATPase epsilon subunit)
MPN598	9.2	9.2	9.9503	9.972642836	10.34	316.92	360.82	atpD	ATP synthase subunit beta (EC 3.6.3.14) (F-ATPase subunit beta) (ATP synthase F1 sector subunit beta)
MPN599	11.8	11.5	9.4160	9.347512829	9.8429	28.92	37.48	atpG	ATP synthase gamma chain (ATP synthase F1 sector gamma subunit) (F-ATPase gamma subunit)
MPN600	12.1	11.6	9.9595	9.837246041	10.07	205.87	200.18	atpA	ATP synthase subunit alpha (EC 3.6.3.14) (F-ATPase subunit alpha) (ATP synthase F1 sector subunit alpha)
MPN601	12.7	11.8	11.0966	10.93095847	9.407	19.82	30.42	atpH	ATP synthase delta chain (F-ATPase delta chain)
MPN602	12.8	11.8	10.9495	11.15350943	10.403	55.88	76.12	atpF	ATP synthase B chain
MPN603	12.9	12.5	11.0452	11.82205861	10.745	0	13.19	atpE	ATP synthase C chain (EC 3.6.3.14) (Lipid-binding protein)
MPN604	11.5	11.3	11.0535	10.39916292	9.9614	26.67	13.88	atpB	ATP synthase A chain (F-ATPase subunit 6) (ATP synthase F0 sector subunit A)
MPN605	10.3	11.0	10.7532	9.926263298	10.089	0	0	mpn65	Conserved hypothetical protein MPN_605
MPN606	12.4	13.0	11.7121	11.77979594	11.338	1183.11	1137.37	eno	Enolase (EC 4.2.1.11) (2-phosphoglycerate dehydratase) (2-phospho-D-glycerate hydro-lyase)
MPN607	10.3	11.3	5.5759	6.683294904	8.75	41.35	40.07	pmsR	Peptide methionine sulfoxide reductase msrA (Protein-methionine-S-oxide reductase) (EC 1.8.4.11) (Peptide-methionine (S)-S-oxide reductase) (Peptide Met(O) reductase)

Continued on next page

Table S1 – Transcriptome and Proteome data – continued from previous page

ORF	RNA micro-arrays 6h	RNA micro-array 96h	RNAseq 6h	RNAseq 96h	Tiling 37 average expression level	Protein copy number per cell 6h	Protein copy number per cell 96h	Protein name	Function
MPN608	7.5	7.6	10.1295	9.064570947	7.6628	29.73	26.48	phoU	Phosphate transport system protein phoU homolog;
MPN609	9.2	8.6	8.6812	7.559711138	7.5233	29.49	32.27	pstB	Phosphate import ATP-binding protein pstB (EC 3.6.3.27) (Phosphate-transporting ATPase) (ABC phosphate transporter)
MPN610	8.2	8.5	7.7674	7.787874801	7.8547	13.08	8.37	pstA	Phosphate transport system permease protein pstA homolog
MPN611	9.9	11.5	10.2302	10.40130076	9.0807	80.45	79.36	pstS	Phosphate-binding protein pstS (PBP)
MPN612	7.5	9.4	7.0237	6.837414234	6.3747	0	0		Conserved hypothetical protein MPN_612
MPN613	5.7	6.1	3.3981	5.178328298	5.8179	0	0		Conserved hypothetical protein MPN_613
MPN614	6.8	7.3	6.1710	5.554810584	6.0143	0	0		Conserved hypothetical protein MPN_614
MPN615	9.0	9.2	8.6913	8.239112106	6.9543	2.49	0.66	hsdS	Putative type I restriction enzyme S protein (HsdS)
MPN616	12.3	11.8	11.5578	10.81408291	9.5657	108.07	100.06	rpsI	30S ribosomal protein S9
MPN617	13.6	13.8	11.4027	12.68473406	10.807	19.99	23.01	rplM	50S ribosomal protein L13
MPN618	8.0	8.2	8.6512	8.858213181	8.5723	86.28	59.85	dnaX	DNA polymerase III subunit gamma/tau (EC 2.7.7.7)
MPN619	9.5	8.3	7.8214	6.704155369	7.7293	36.35	35.53	uvrA	UvrABC system protein A (UvrA protein) (Excinuclease ABC subunit A)
MPN620	7.3	7.7	9.4169	9.360230471	8.2732	15.29	16.82		Conserved hypothetical protein MPN_620
MPN621	12.0	11.1	11.0303	9.614307421	9.3335	82.05	83.58	rnj	Probably non-catalytic ribonuclease J1
MPN622	12.4	13.2	13.4537	13.82020359	10.82	13.52	16.57	rpsO	30S ribosomal protein S15
MPN623	11.9	11.9	6.9699	7.780362263	9.66	32.14	10.04	deaD	Probable ATP-dependent RNA helicase (EC 3.6.1.-)
MPN624	14.9	13.7	9.8112	9.278441875	10.4	30.41	18.04	rpmB	50S ribosomal protein L28
MPN625	13.0	12.8	8.4892	8.34480028	10.5	330.04	350.61	osmC	Peroxiredoxin osmC
MPN626	8.1	10.5	3.2619	6.088133807	6.38	0	0		Probable RNA polymerase sigma-D factor (sigD)
MPN627	11.6	11.4	9.6367	9.42671674	9.5051	186.61	199.76	ptsI	Phosphoenolpyruvate-protein phosphotransferase (EC 2.7.3.9) (Phosphotransferase system, enzyme I)
MPN628	11.2	10.0	8.6135	8.216715494	9.3525	94.91	117.79	pgm	2,3-bisphosphoglycerate-independent phosphoglycerate mutase (Phosphoglyceromutase) (BPG-independent PGAM) (iPGM) (EC 5.4.2.1)
MPN629	11.7	12.0	9.5885	9.83411616	9.575	127.05	126.62	tim	Triosephosphate isomerase (TIM) (EC 5.3.1.1) (Triose-phosphate isomerase)
MPN630	10.3	9.9	7.8441	7.658774135	7.767	13.73	14.34	yfiB	Uncharacterized protein MPN_630
MPN631	11.9	11.4	7.2746	8.785093432	9.92	195.74	195.05	tsf	Elongation factor Ts (EF-Ts)
MPN632	8.6	7.5	5.9258	6.180975056	8.26	13.05	13.14	pyrH	Uridylate kinase (UK) (EC 2.7.4.22) (Uridine monophosphate kinase) (UMP kinase) (UMPK)
MPN633	11.6	10.9	5.5558	4.614668056	7.5	0	0		Conserved hypothetical protein MPN_633
MPN634	8.9	8.0	4.5411	4.56229984	6.26	0	0	ywaC	Putative GTP-pyrophosphokinase YwaC (RelA-like) (EC 2.7.6.5)
MPN635	8.5	7.6	1.9248	2.88507673	6.06	0	0		Conserved hypothetical protein MPN_635
MPN636	11.4	11.5	7.1881	8.167085281	8.2	279.69	265.26	frr	Ribosome-recycling factor (RRF) (Ribosome-releasing factor)
MPN637	9.0	8.5	5.0188	4.614746857	6.83	0	4.06	cdsA	Putative phosphatidate cytidylyltransferase (EC 2.7.7.41) (CDP-diacylglycerol synthase) (CDS) (CTP:phosphatidate cytidylyltransferase) (CDP-DG synthetase) (CDP-DAG synthase)
MPN638	10.2	11.8	7.5620	9.117202813	9.78	321.78	277.22	hsdS	Putative type I restriction enzyme S protein (HsdS)
MPN639	7.5	9.2	8.6698	9.309718846	7.4012	11.59	5.3		Conserved hypothetical lipoprotein MPN_639
MPN640	8.9	9.0	7.3645	5.941547529	6.5489	0	0		Conserved hypothetical lipoprotein MPN_640
MPN641	8.2	8.6	7.0470	7.175741984	6.556	8.06	1.81		Conserved hypothetical lipoprotein MPN_641
MPN642	6.9	8.1	8.9825	8.800049613	6.8233	8.98	4.65		Conserved hypothetical lipoprotein MPN_642
MPN643	6.8	7.8	9.2309	8.706596058	7.0599	6.79	4.16		Conserved hypothetical lipoprotein MPN_643
MPN644	9.3	10.4	7.3862	7.764505313	7.0004	0	0		Conserved hypothetical lipoprotein MPN_644
MPN645	7.5	7.5	6.9245	7.060420549	7.2975	0	0		Conserved hypothetical lipoprotein MPN_645
MPN646	7.8	8.7	7.5050	6.461233715	7.2139	0	0		Conserved hypothetical lipoprotein MPN_646
MPN647	8.6	10.4	8.5288	8.242756215	8.3785	0	2.83		Conserved hypothetical lipoprotein MPN_647
MPN648	9.8	11.2	10.1944	10.69877183	7.8758	0	0		Conserved hypothetical lipoprotein MPN_648
MPN649	7.9	7.1	6.9875	4.509532957	6.3666	0	0		Uncharacterized protein MPN_649
MPN650	10.1	10.3	9.5526	9.548711045	7.1954	0	0		Uncharacterized lipoprotein MPN_650
MPN651	5.2	NA	0.0461	2.449993848	6	0	0	mtlA	PTS system mannitol-specific EIICB component (EIICB-Mtl) (EII-Mtl)
MPN652	6.1	6.0	3.7317	5.466599454	6.79	9.5	18.21	mtlD	Mannitol-1-phosphate 5-dehydrogenase (EC 1.1.1.17)

Continued on next page

Table S1 – Transcriptome and Proteome data – continued from previous page

ORF	RNA micro-arrays 6h	RNA micro-array 96h	RNAseq 6h	RNAseq 96h	Tiling 37 average expression level	Protein copy number per cell 6h	Protein copy number per cell 96h	Protein name	Function
MPN653	8.2	9.9	5.2935	6.476819722	7.6	27.43	18.89	mtlF	Mannitol-specific phosphotransferase enzyme IIA component (EC 2.7.1.-) (PTS system mannitol-specific EIIA component) (EIIA-Mtl) (EIII-Mtl)
MPN654	7.3	8.4	6.8734	7.089065243	6.4475	29.7	0		Conserved hypothetical lipoprotein MPN_654
MPN655	9.2	10.2	7.1377	6.37704332	8.23	7.66	26.61		Uncharacterized protein MPN_655
MPN656	8.2	5.0	8.1144	7.401724192	6.9073	7.17	0.58	rbgA	Ribosome biogenesis GTPase A
MPN657	11.3	9.3	8.6827	7.264252932	7.74	0	1.66		Uncharacterized protein MPN_657
MPN658	11.9	10.6	9.8656	8.818421694	7.9368	89.49	109.31	rplS	50S ribosomal protein L19
MPN659	13.4	12.5	10.7575	9.827984191	8.5378	14.33	5.61	trmD	tRNA (guanine-N(1)-)-methyltransferase (EC 2.1.1.31) (M1G-methyltransferase) (tRNA [GM37] methyltransferase)
MPN660	13.6	13.6	10.2742	10.34076302	10.004	61.11	77.69	rpsP	30S ribosomal protein S16
MPN661	9.4	10.8	9.0375	8.866031467	8.6558	16.13	18.97		Conserved hypothetical protein MPN_661
MPN662	11.4	11.8	11.9366	12.26633675	10.126	162.33	143.14	pilB	Peptide methionine sulfoxide reductase msrB (EC 1.8.4.12) (Peptide-methionine (R)-S-oxide reductase)
MPN663	11.8	11.1	11.1506	11.70210923	9.7544	30.4	30.12	ytpR	Putative tRNA-binding protein ytpR
MPN664	12.6	11.8	11.7497	11.38798559	9.7544	57.99	49.23	degV	Uncharacterized protein, DegV family
MPN665	15.3	14.7	13.6103	13.63930953	11.425	2630.78	2477.83	tuf	Elongation factor Tu (EF-Tu)
MPN666	11.3	12.4	6.9112	8.144969256	9.71	19.15	15.68		Conserved hypothetical protein MPN_666
MPN667	10.8	11.3	4.8886	7.005413111	8.98	37.14	35.55	gtaB	UTP–glucose-1-phosphate uridylyltransferase (EC 2.7.7.9) (UDP-glucose pyrophosphorylase) (UDPGP) (Alpha-D-glucosyl-1-phosphate uridylyltransferase) (Uridine diphosphoglucose pyrophosphorylase)
MPN668	12.1	14.1	11.6042	11.99859475	10.956	412.08	381.82	osmC	Organic hydroperoxide resistance protein-like
MPN669	9.9	10.0	4.8484	5.920052861	8.12	31.93	32.86	tyrS	Tyrosyl-tRNA synthetase (EC 6.1.1.1) (Tyrosine–tRNA ligase) (TyrRS)
MPN670	10.8	10.8	10.7832	11.18135134	9.8024	73.44	80.23		Conserved hypothetical protein MPN_670
MPN671	11.9	11.2	9.6327	8.878397235	9.5159	197.78	188.49	ftsH	Cell division protease ftsH homolog (EC 3.4.24.-)
MPN672	12.1	11.7	10.8436	11.41854351	9.5708	8.37	35.15	hpt	Hypoxanthine-guanine phosphoribosyltransferase (HGPRTase) (HGPRT) (EC 2.4.2.8)
MPN673	13.4	13.3	12.7524	13.09335537	10.107	164.43	155.87	ygbB	2-C-methyl-D-erythritol 2,4-cyclodiphosphate synthase-like
MPN674	12.3	14.5	7.9735	9.470239217	11.4	960.99	995.72	ldh	L-lactate dehydrogenase (L-LDH) (EC 1.1.1.27)
MPN675	8.8	10.3	10.0565	9.921451636	7.9308	5.76	0		Uncharacterized protein MPN_675
MPN676	7.6	7.1	1.8149	3.828331957	6.19	0	0		Uncharacterized protein MPN_676
MPN677	11.0	10.2	9.0345	8.386929044	8.3743	40.29	46.54	ywfO	DGT (deoxyguanosinetriphosphate triphosphohydrolase) (EC 3.1.5.1). Putative Transcription factor?.
MPN678	11.6	10.9	9.8439	9.495711612	9.6892	89.52	95.2	gltX	Glutamyl-tRNA synthetase (EC 6.1.1.17) (Glutamate–tRNA ligase) (GluRS)
MPN679	10.6	7.9	9.3209	9.178558846	9.2182	12.27	6.93	ksgA	Dimethyladenosine transferase (EC 2.1.1.-) (S-adenosylmethionine-6-N', N'-adenosyl(rRNA) dimethyltransferase) (16S rRNA dimethylase) (High level kasugamycin resistance protein ksgA) (Kasugamycin dimethyltransferase)
MPN680	10.7	9.4	8.9790	7.858608713	8.3607	13.48	12.91	yidC	Inner membrane protein oxaA; Membrane insertion of proteins
MPN681	11.2	10.0	10.6819	9.724424036	9.3957	0	0	rnpA	Ribonuclease P protein component (RNaseP protein) (RNase P protein) (EC 3.1.26.5) (Protein C5)
MPN682	12.8	13.3	12.1181	10.54259273	10.543	10.31	25.94	rpmH	50S ribosomal protein L34
MPN683	9.8	10.0	9.5683	8.420612413	8.9524	39.88	20.73	devA	Putative ABC transporter ATP-binding protein MPN_683
MPN684	10.8	11.3	9.4846	8.216992937	8.9067	60.98	26.78	mpn684	Uncharacterized ABC transporter permease MPN_684
MPN685	12.3	13.4	11.5015	10.86204727	10.677	104.82	78.4	cysA	Sulfate/thiosulfate import ATP-binding protein cysA (EC 3.6.3.25)
MPN686	10.2	10.3	9.9552	9.050107193	8.6256	45.6	32.35	dnaA	Chromosomal replication initiator protein dnaA
MPN687	11.4	11.3	10.1436	8.419277128	9.5536	24.95	17.87		Uncharacterized protein MPN_687
MPN688	11.7	11.9	8.8688	9.819301537	9.4511	201.07	199.15	soj	ParA family protein MPN_688
Mpnr01			17.8944	17.72252736	12.4				
Mpnr02			17.4180	17.27251822	12.3				
Mpnr03			14.1692	14.88961205	12				
MPNt01			17.7706	17.50856106	10.141				
MPNt02			15.0814	15.91101901	10.727				
MPNt03			12.7277	12.90097993	10.009				
MPNt04			20.3136	20.30722873	11.217				
MPNt05			13.1869	14.04346899	11.6				
MPNt06			11.1568	11.43924538	11.1				
MPNt07			10.9151	11.38827376	11.3				
MPNt08			11.6073	12.32120901	11.7				
MPNt09			10.5035	11.55565428	12.3				
MPNt10			15.1982	15.83326073	11.4				

Continued on next page

Table S1 – Transcriptome and Proteome data – continued from previous page									
**ORF**	**RNA micro-arrays 6h**	**RNA micro-array 96h**	**RNAseq 6h**	**RNAseq 96h**	**Tiling 37 average expression level**	**Protein copy number per cell 6h**	**Protein copy number per cell 96h**	**Protein name**	**Function**
MPNt11			15.7198	16.38692481	11.6				
MPNt12			11.6135	12.01101993	9.64				
MPNt13			12.7400	13.47278439	10.4				
MPNt14			17.3570	17.37176525	9.9189				
MPNt15			12.2811	13.00307935	11.8				
MPNt16			14.2072	14.83646335	12				
MPNt17			15.3970	15.04770664	10.385				
MPNt18			17.1642	17.14529471	11.248				
MPNt19			14.1501	14.27748833	10.207				
MPNt20			18.3182	18.28466355	11.235				
MPNt21			16.9906	16.52197123	11.86				
MPNt22			15.5768	15.6890072	11.269				
MPNt23			12.4330	12.86204463	10.1				
MPNt24			10.8611	11.78078237	11.4				
MPNt25			8.2994	9.6754879	11.2				
MPNt27			15.1640	14.77145507	8.8129				
MPNt28			16.7656	16.60288071	9.4137				
MPNt29			18.2710	18.06766745	11.883				
MPNt30			16.9007	16.62281683	10.196				
MPNt31			13.1952	13.41201056	11.193				
MPNt32			13.1098	13.30416656	11.16				
MPNt33			15.5803	15.57179877	11.191				
MPNt34			7.2057	7.533684199	9.98				
MPNt35			16.9002	16.94873483	11.301				
MPNt36			14.9706	15.82156448	11.301				
MPNt37			10.9789	11.21225902	11.887				
