# Supplementary material for: Comprehensive Methylome Characterization of Mycoplasma genitalium and Mycoplasma pneumoniae at Single-Base Resolution
Source: PLoS Genet. 2013 Jan 3;9(1):e1003191. doi: 10.1371/journal.pgen.1003191 (PMC3536716; doi:10.1371/journal.pgen.1003191)
Supplement: Table S2 — Enriched regions for 5′-GAN7TAY-3′/3′-CTN7 ATR-5′ (a) and 5′-CTAT-3′ (b) motifs. Table S2c is the legend for the functions assigned to the different COG categories. (PDF) [file pgen.1003191.s003.pdf]

Table S2a – 5'-GAN$_7$TAY-3'/ 3'-CTN$_7$ATR-5' hot spots in *M. pneumoniae* genome

Position	Mean Nr of motifs	ORFs	MPNs	COG Category
682000	5.104	MPN560_MPN561_MPN562	MPNs099	C
797000	5.094	MPN672_MPN673		F
779000	5.042	MPN653_MPN654	MPNs116	G
416500	6.25	MPN348	MPNs324	H
687500	6.612	MPN566_MPN567	MPNs102	I
462000	5.278	MPN384		J
427500	5.128	MPN357	MPNt14	L
741000	5.606	MPN618		L
108000	6.354	MPN085	MPNs235	M
109000	6.334	MPN086	MPNs015_MPNs016	M
109500	5.222	MPN087	MPNs016	M
188500	5.328	MPN142		M
200500	5.366	MPN152		M
339000	6.198	MPN284		M
529000	6.332	MPN436_MPN437		M
552000	7.916	MPN452		M
596500	6.098	MPN489_MPN490	MPNs088_MPNs342	M
616500	6.258	MPN506		M
707000	5.108	MPN584_MPN585		M
708500	5.536	MPN585_MPN586		M
711000	5.726	MPN588_MPN589	MPNs362	M
711500	10.888	MPN588_MPN589	MPNs107_MPNs362	M
713000	5.37	MPN590_MPN591		M
165000	7.438	MPN127		N
177500	8.73	MPN137		N
715000	6.022	MPN592		O
811500	5.528	MPN684		P
111000	6.394	MPN088		S
700500	6.212	MPN577		S
735500	7.316	MPN612		S
736000	5.248	MPN612_MPN613		S
748000	5.524	MPN620_MPN621		S
638000	5.532	MPN518	MPNs093	T
618500	5.408	MPN508	MPNs091	U
347500	5.522	MPN289		V
413000	5.34	MPN345		V
122000	5.276	Mpnr02	MPNs242	
123000	6.634	Mpnr03	MPNs242	
108500	10.526		MPNs015_MPNs235	
172500	5.8		MPNs021	
500	10.004		MPNs200_MPNs201_MPNs381	
113000	5.27		MPNs238	
337500	5.478		MPNs302	
364000	5.7		MPNs310	
364500	7.37		MPNs310	
415000	5.798		MPNs322	
415500	5.526		MPNs322	
180500	7.81		MPNs383	
106000	5.516	Intergenic region		
120500	6.894	Intergenic region		
145000	5.382	Intergenic region		
165500	6.218	Intergenic region		
244000	5.428	Intergenic region		
343500	5.09	Intergenic region		
646500	5.226	Intergenic region		
763500	5.58	Intergenic region		
764000	5.262	Intergenic region		
777500	6.102	Intergenic region		

Table S2b – 5'-CTAT-3' hot spots in *M. pneumoniae* genome

Position	Mean Nr of motifs	ORFs	MPNs	COG Category
452500	3.094	MPN376		A
65000	3.568	MPN051	MPNs221	C
650500	4.302	MPN529	MPNs352	D
651000	3.656	MPN529	MPNs352	D
557000	3.132	MPN456	MPNs078	E
574000	3.548	MPN470_MPN471_MPN472		E
330500	3.402	MPN279		J
676500	3.396	MPN556		J
295500	3.174	MPN243	MPNs288	K
634500	3.388	MPN516		K
203500	3.15	MPN153		L
427500	4.52	MPN357	MPNt14	L
505000	3.522	MPN419		L
61000	3.77	MPN049		M
183000	3.42	MPN141		M
546000	3.388	MPN447_MPN448	MPNs073	M
569500	3.29	MPN465_MPN466_MPN467	MPNs083	M
12500	3.132	MPN010_MPN011		N
646000	3.336	MPN524	MPNs095	N
794500	3.74	MPN671_MPN671a		O
808500	3.73	MPN684		P
810000	5.052	MPN684		P
811500	4.39	MPN684		P
65500	3.658	MPN052	MPNs221	S
135000	4.476	MPN103_MPN104_MPN104a		S
559500	3.21	MPN457_MPN458		S
560500	3.426	MPN458_MPN459	MPNs080	S
625500	3.82	MPN511_MPN512	MPNs346	S
748000	3.3	MPN620_MPN621		S
348000	3.336	MPN290_MPN291		V
627500	3.482	MPN513_MPN514	MPNs347	V
122000	3.038	Mpnr02	MPNs242	
113000	3.05		MPNs238	
365500	3.192		MPNs311	
408000	3.064		MPNs321	
415000	3.068		MPNs322	
409500	5.332		MPNs323	
663000	3.674		MPNs354	
180500	5.666		MPNs383	
12000	3.134	Intergenic region		
59500	4.278	Intergenic region		
75000	3.484	Intergenic region		
148000	3.484	Intergenic region		
268500	3.078	Intergenic region		
371000	3.104	Intergenic region		
410000	3.562	Intergenic region		
777500	4.102	Intergenic region		

Table S2c – COG categories

COG	Function
A	Membrane Proteins of unknown function
C	Energy production and conversion, Coenzyme metabolism
D	Cell division and chromosome partitioning
E	Amino acid transport and metabolism
F	Nucleotide transport and metabolism; coenzyme metabolism
G	Carbohydrate transport and metabolism
H	Coenzyme metabolism
I	Lipid metabolism
J	Translation, ribosomal structure and biogenesis
K	Transcription
L	DNA replication, recombination and repair
M	Cell envelope biogenesis, outer membrane
N	Cell motility and secretion
O	Post-translational modification, protein turnover, chaperones
P	Inorganic ion transport and metabolism
R	General function prediction only
S	Function unknown
T	Signal transduction mechanisms
U	Intracellular trafficking, secretion and vesicular transport
V	Defense mechanisms
