# Supplementary material for: Comprehensive Methylome Characterization of Mycoplasma genitalium and Mycoplasma pneumoniae at Single-Base Resolution
Source: PLoS Genet. 2013 Jan 3;9(1):e1003191. doi: 10.1371/journal.pgen.1003191 (PMC3536716; doi:10.1371/journal.pgen.1003191)
Supplement: Table S3 — ORFs with a 5′-CTAT-3′ motif that changed from non-methylated to methylated state from 6 to 96 h. (PDF) [file pgen.1003191.s004.pdf]

Table S3 – ORFs with a 5'-CTAT-3' motif that changed from non-methylated to methylated state from 6 to 96 h

ORF	Strand	Start codon	Stop codon	RNA arrays 6h	RNA arrays 96h	Protein name	Function	COG category
MPN376	-	452995	449573	12	13.9		Uncharacterized protein MPN_376	A
MPN315	+	373829	374755	13.6	13.9	mraW	S-adenosyl-L-methionine-dependent methyltransferase mraW (EC 2.1.1.-)	D
MPN076	-	90993	89299	8.3	10	uhpT	Hexose phosphate transport protein	G
MPN047	+	55942	57297	8.8	10	PncB	Nicotinate phosphoribosyl transferase (EC 2.4.2.12)	H
MPN018	+	19325	21196	10.8	11.2	pmd1	Putative ABC transporter ATP-binding protein MPN_018	I
MPN023	+	28245	29783	9.1	8.8	metS	Methionyl-tRNA synthetase (EC 6.1.1.10) (Methionine–tRNA ligase) (MetRS)	J
MPN679	-	804225	803434	10.6	7.9	ksgA	Dimethyladenosine transferase (EC 2.1.1.-) (S-adenosylmethionine-6-N', N'-adenosyl(rRNA) dimethyltransferase) (16S rRNA dimethylase) (High level kasugamycin resistance protein ksgA) (Kasugamycin dimethyltransferase)	J
MPN626	+	752121	752543	8.1	10.5		Probable RNA polymerase sigma-D factor (sigD)	K
MPN378	+	453650	456268	10.1	9.8	dnaE	DNA polymerase III subunit alpha (EC 2.7.7.7)	L
MPN039	+	47194	48210	12	12.3		Conserved hypothetical protein MPN_039	M
MPN146	+	193071	193868	12.8	15.5		Conserved hypothetical protein MPN_146	M
MPN200	+	241983	244379	10	10.5		Conserved hypothetical lipoprotein MPN_200	M
MPN284	+	337770	340154	11	10.5		Uncharacterized lipoprotein MPN_284	M
MPN567	-	690453	687343	10.9	10.5	p2	Protein P200	M
MPN590	-	712690	712037	11	12.6		Conserved hypothetical lipoprotein MPN_590	M
MPN100	+	129626	130009	9.3	12.5		Uncharacterized protein MPN_100	N
MPN607	+	727599	728072	10.3	11.3	pmsR	Peptide methionine sulfoxide reductase msrA (Protein-methionine-S-oxide reductase) (EC 1.8.4.11) (Peptide-methionine (S)-S-oxide reductase) (Peptide Met(O) reductase)	OV
MPN684	-	812540	806892	10.8	11.3	mpn684	Uncharacterized ABC transporter permease MPN_684	P
MPN083	+	103941	105542	11.3	12.9		Uncharacterized lipoprotein MPN_083	S
MPN612	-	736070	733077	7.5	9.4		Conserved hypothetical protein MPN_612	S
MPN475	-	580757	579408	9.1	8.7	engA	GTP-binding protein engA	T
MPN680	-	805375	804218	10.7	9.4	yidC	Inner membrane protein oxaA; Membrane insertion of proteins	U
MPN343	+	409871	410863	9.6	9.3		Putative type-1 restriction enzyme specificity protein MPN_343 (S.mpnORFDP) (Type I restriction enzyme specificity protein MPN_343) (S protein)	V
Mpnr01	+	118312	119825					
Mpnr02	+	120057	122962					
