# Supplementary material for: Comprehensive Methylome Characterization of Mycoplasma genitalium and Mycoplasma pneumoniae at Single-Base Resolution
Source: PLoS Genet. 2013 Jan 3;9(1):e1003191. doi: 10.1371/journal.pgen.1003191 (PMC3536716; doi:10.1371/journal.pgen.1003191)
Supplement: Table S4 — Functional analysis of genes located in “hot spots of methylation”. COG categories of genes located in enriched regions for 5′-CTAT-3′ (a) and 5′-GAN7TAY-3′/3′-CTN7 ATR-5′ (b) motifs have been compared to those categories in whole genome by using Fisher's test. A functional enrichment is considered significant when the Pvalue<0.05. (PDF) [file pgen.1003191.s005.pdf]

Table S4a – Functional analysis for 5'-CTAT-3' motif

COG Category	Nr of ORFs in genome	Nr of ORFs in enriched regions	Fisher test ODDS RATIO	P-value (enrichment)
A	26	0	0.00	1.00
C	25	1	0.59	0.82
D	16	0	0.00	1.00
E	19	0	0.00	1.00
F	25	1	0.59	0.82
G	37	1	0.39	0.92
H	12	2	2.56	0.22
I	26	1	0.57	0.83
J	119	2	0.22	1.00
K	19	0	0.00	1.00
L	48	3	0.93	0.63
M*	123	18	2.96	0.00
N	26	2	1.16	0.53
O	32	2	0.93	0.64
P	20	0	0.00	1.00
R	17	0	0.00	1.00
S	54	6	1.76	0.16
T	8	1	1.89	0.44
U	15	1	1.00	0.65
V*	23	5	3.53	0.03

*Enriched COG category

Table S4b – Functional analysis for 5'-GAN_7TAY-3'/ 3'-CTN_7ATR-5' motif

COG Category	Nr of ORFs in genome	Nr of ORFs in enriched regions	Fisher test ODDS RATIO	P-value (enrichment)
A	26	1	0.66	0.79
C	25	1	0.68	0.77
D	16	1	1.08	0.62
E	19	3	2.86	0.11
F	25	0	0.00	1.00
G	37	0	0.00	1.00
H	12	1	1.45	0.52
I	26	1	0.66	0.79
J	119	4	0.53	0.93
K	19	2	1.86	0.32
L	48	3	1.08	0.54
M	123	8	1.15	0.43
N	26	3	2.07	0.21
O	32	1	0.53	0.85
P	20	0	0.00	1.00
R	17	0	0.00	1.00
S*	54	7	2.49	0.04
T	8	0	0.00	1.00
U	15	0	0.00	1.00
V	23	4	3.21	0.05

*Enriched COG category
