# Supplementary material for: Comprehensive Methylome Characterization of Mycoplasma genitalium and Mycoplasma pneumoniae at Single-Base Resolution
Source: PLoS Genet. 2013 Jan 3;9(1):e1003191. doi: 10.1371/journal.pgen.1003191 (PMC3536716; doi:10.1371/journal.pgen.1003191)
Supplement: Table S5 — Methylation in promoter sequences. Genomic regions 40 bp upstream from the TSS are considered as putative promoter sequences. This table shows the 197 out 663 ORFs with assigned TSS that showed methylation at the promoter region. First column indicates the ORF name. The rest of columns indicate the sequence of the motifs and the genome positions, as well as, the strand for these motifs. The two last columns indicate the function and the COG category respectively. If the ORF is a new identified RNA (MPNs) the overlapping ORF is indicated in the function column and the COG category of the overlapping ORF is annotated in the last column. (PDF) [file pgen.1003191.s006.pdf]

Table S5 – Methylation in promoter sequences

ORF	Motif1	Motif2	Motif3	Motif4	Motif5	Motif6	Motif7	Motif8	Motif9	P1	P2	P3	P4	P5	P6	P7	P8	P9	Str1	Str2	Str3	Str4	Str5	Str6	Str7	Str8	Str9	Protein name (Overlapping gene for MPNs)	COG Category
MPN001	ATAA	TGAT								642	650								+	-								dnaN	L
MPN010	CTAT	CGAA	GTAT	CTAT						12321	12336	12307	12340						+	+	-	-						-	N
MPN012	GTAA	AGAT								14315	14323								+	-								-	M
MPN027	CTAT									33054									-									rimL	J
MPN028	CTAT									33011									-									trsB	M
MPN029	CTAT	CTAT								33800	33780								+	-								efp	J
MPN041	GTAG									48513									-									-	M
MPN045	CTAT									53050									-									hisS	J
MPN046	CTAT									54139									+									aspS	J
MPN049	AGAT	ATAC								59443	59451								+	-								-	M
MPN051	CTAT									64667									-									glpD	C
MPN052	CTAT									64798									+									-	S
MPN059	AGAA	GTAA								75172	75180								+	-								Gcp	O
MPN061	CTAT									79016									-									ffh	U
MPN085	CTAT									107237									+									-	M
MPN088	CTAT									110200									-									-	S
MPN091	CTAT									113607									-									-	S
MPN104	CTAT									134175									-									-	N
MPN104a	GTAT	TGAA								134953	134961								+	-								-	-
MPN105	CTAT	CTAT	CTAT							135695	135708	135709							+	+	-							pheS	J
MPN115	ATAA									150552									+									infC	J
MPN127	ATAA	AGAC								164589	164597								+	-								-	N
MPN127a	ATAA	CTAT	AGAG							165753	165776	165761							+	+	-							-	-
MPN129	CTAT									167583									+									-	M
MPN131	GTAT	GGAA								170000	170008								+	-								-	M
MPN137	CTAT									178255									+									-	N
MPN153a	TGAG	CGAA	GTAT							205471	205510	205479							+	+	-							-	-
MPN157	CTAT									209875									+									-	M
MPN188	CTAT									230566									-									rpmJ	J
MPN191	CTAT									231374									+									rpoA	K
MPN194	CTAT									233567									-									CbiO 2	P
MPN210	CTAT									257175									-									secA	U
MPN228	CTAT	CTAT	CTAT							279724	279732	279736							-	-	-							rpsF	J
MPN246	GTAA									297578									+									gmk	F
MPN250	CTAT									301072									+									pgiB	G
MPN265	ATAA									318129									-									trpS	J
MPN266	CTAT									318110									+									spxA	K
MPN274	CTAT									325830									+									-	P
MPN276	ATAA									327071									-									-	S
MPN277	CTAT									326795									-									lysS	J
MPN281	AGAT	GTAA								333219	333227								+	-								-	M
MPN285	AGAA	CTAT	GTAG							340658	340670	340666							+	+	-							prrB	V
MPN289	CTAT									346934									-									hsdS1B	V
MPN302	TGAT	ATAA								355661	355669								+	-								pfk	G
MPN319	GGAT									378385									-									-	E
MPN331	ATAG	CTAT								388904	388903								+	-								tig	O
MPN343	CTAT									409401									-									-	V
MPN348	CTAT	CTAT								415132	415113								+	-								mthfs	H
MPN350	CTAT									418152									+									plsY	I
MPN355	CTAT									424243									+									yacO	J
MPN358	CTAT									427641									+									-	A
MPN373	CTAT	CTAT	GTAA	TGAT						446613	446620	446627	446635						+	+	+	-						-	M
MPN377	CTAT									453354									+									-	A
MPN379	CTAT									456054									-									polA	L
MPN381	AGAG	ATAT								458028	458036								+	-								yidA	R
MPN386	CTAT									463895									-									yaaF	F
MPN394	AGAC									472929									-									nox	C
MPN396	ATAA									476492									-									secD	U

Continued on next page

Table S5 – Methylation in promoter sequences – continued from previous page																													
ORF	Motif1	Motif2	Motif3	Motif4	Motif5	Motif6	Motif7	Motif8	Motif9	P1	P2	P3	P4	P5	P6	P7	P8	P9	Str1	Str2	Str3	Str4	Str5	Str6	Str7	Str8	Str9	Protein name (Overlapping gene for MPNs)	COG Category
MPN397	TGAC									476484									+									spoT	TF
MPN400	CTAT	GGAG	AGAA							482119	482145	482114							+	+	-							-	A
MPN407	CTAT									490223									+									-	I
MPN412a	CGAT									497073									+									-	-
MPN427	GGAC	CTAT	GTAT							514447	514454	514455							+	+	-							yidA	R
MPN456	TGAT	CTAT	ATAA							558438	558453	558446							+	+	-							-	E
MPN471	GTAT	AGAT	CTAT	AGAA	ATAA					574257	574263	574260	574265	574271					+	+	-	-	-					rpmG	J
MPN474a	CGAC	ATAA								579421	579429								+	-								-	-
MPN482	AAGC	AGAT	GTAT							586897	586902	586910							+	+	-							-	A
MPN487	CTAT									590468									-									nifS	O
MPN488a	GTAA									592613									+									-	-
MPN498	CTAT									604881									+									araD	G
MPN506a	CTAT									616157									-									-	-
MPN508	CTAT	CTAT								619751	619776								+	+								-	U
MPN517	CTAT	ATAA								637073	637076								+	-								yhdA	H
MPN521	CTAT									642724									-									ygl3	J
MPN531	CTAT									653975									-									clpB	O
MPN537	CTAT									659763									+									mucB	L
MPN542	CGAT	ATAC								661142	661150								+	-								-	A
MPN549	CTAT									670464									+									recJ	L
MPN557	TGAA	GTAA								677297	677305								+	-								gidA	D
MPN567	CTAT									690539									-									p2	M
MPN582a	CGAA									705563									+									-	-
MPN594	CTAT									717065									+									-	M
MPN607	ATAT	AGAA								727545	727553								+	-								pmsR	OV
MPN620	CTAT									747747									-									-	S
MPN625	CTAT									751457									-									osmC	OV
MPN652	CTAT	CTAT								777436	777444								+	+								mtlD	G
MPN665	TGAA									789406									-									tuf	J
MPN670a	CAGC									792899									+									-	-
MPN676	AGAA	CTAT	GTAT							799762	799747	799770							+	-	-							-	S
Mpnr01	CTAT									118194									-									-	-
MPNs005	CTAT									13367									+									MPN011/MPN012	M
MPNs010	CTAT									88154									-									MPN074	J
MPNs013	CTAT	CTAT								96163	96136								+	-								MPN079/MPN080	G
MPNs015	CTAT									108593									-									MPN085/MPN086	M
MPNs019	CTAT									166996									+									MPN128/MPN129	M
MPNs026	CTAT									240980									+									MPN199	M
MPNs027	CTAT	ATAA	CTAT							253186	253203	253175							+	+	-							MPN207/MPN208	G
MPNs036	CTAT									398580									+									MPN336	H
MPNs040	CTAT									450678									-									MPN376	A
MPNs044	CTAT	CTAT								460383	460378								+	-								MPN384	J
MPNs047	CTAT									468448									-									MPN391	C
MPNs048	ATAG	CTAT	TGAT							470444	470443	470452							+	-	-							MPN393	C
MPNs051	CTAT									482007									-									MPN400/MPN401	A
MPNs052	CTAT									482218									-									MPNt18	-
MPNs054	CTAT	CTAT								485121	485160								+	+								MPN403	S
MPNs055	CTAT									490295									+									MPN408	M
MPNs061	CTAT									517143									+									MPN429	G
MPNs062	ATAG	AGAG								518830	518838								+	-								MPN431	P
MPNs063	CTAT									530396									+									MPN437	M
MPNs066	CTAT									533770									-									MPN440	M
MPNs067	AGAG									538211									-									MPN444	M
MPNs069	ATAA	TGAG								541478	541486								+	-								MPN444	M
MPNs072	ATAA	TGAT								544782	544790								+	-								MPN447	M
MPNs076	CTAT									550519									+									MPN451/MPN452	L
MPNs077	TGGT									556300									+									MPN456	E
MPNs078	CTAT									556607									+									MPN476	F
MPNs080	ATAG	CTAT								560134	560133								+	-								MPN459	E

Continued on next page

Table S5 – Methylation in promoter sequences – continued from previous page

ORF	Motif1	Motif2	Motif3	Motif4	Motif5	Motif6	Motif7	Motif8	Motif9	P1	P2	P3	P4	P5	P6	P7	P8	P9	Str1	Str2	Str3	Str4	Str5	Str6	Str7	Str8	Str9	Protein name (Overlapping gene for MPNs)	COG Category
MPNs081	CTAT									562623									+									MPN460	P
MPNs088	CTAT									596478									+									MPN489/MPN490	M
MPNs095	AGAA									645856									+									MPN524	N
MPNs100	GGAA	TGAA	ATAA							683611	683647	683619							+	+	-							MPN563	J
MPNs102	CTAT									687557									-									MPN567	M
MPNs106	CTAT									708511									-									MPN586	O
MPNs107	CTAT	CTAT								711416	711408								+	-								MPN589	S
MPNs108	CTAT									716544									-									MPN594	M
MPNs111	CTAT									752332									+									MPN627/MPN626	G
MPNs112	GGAA									757435									+									MPN630	M
MPNs122	ATAG	CTAT								786498	786497								+	-								MPN663	J
MPNs124	CTAT									797281									-									MPN673/MPN674	I
MPNs127	CTAT									813583									+									MPN686	LD
MPNs128	CTAT									701158									-									MPN577/MPN578	S
MPNs200	CTAT									207									-									MPN688/MPN001	D
MPNs201	CTAT									597									-									MPN688/MPN001	D
MPNs206	CTAT									19445									+									MPN018	I
MPNs213	CTAT									33800									+									MPN028	M
MPNs214	CTAT									41208									+									MPN034	L
MPNs217	AGAT	ATAA	ATAT	CGAT						48836	48838	48844	48846						+	+	-	-						MPN041	M
MPNs220	CTAT									57466									-									MPN047/MPN048	H
MPNs221	GTAA									65572									-									MPN052	S
MPNs226	CTAT									82047									+									MPN065	F
MPNs229	CTAT									69326									+									MPN054/MPN055	M
MPNs232	ATAA	CTAT	AGAC							73024	73026	73032							+	-	-							MPN056/MPN057	E
MPNs233	CTAT									96163									+									MPN079	G
MPNs235	CGAC	TGAA	ATAT	ATAT						108245	108251	108253	108259						+	+	-	-						MPN085	M
MPNs238	CTAT									113421									+									MPN090	M
MPNs239	ATAC									114898									+									MPN092	M
MPNs240	CTAT									118112									+									MPN094/MPNr01	N
MPNs242	CTAT									122795									+									MPNr02	-
MPNs244	CTAT									124857									+									MPN096	E
MPNs246	CTAT									126575									+									MPN097	M
MPNs251	CTAT									141666									-									MPN108/MPN109	L
MPNs261	ATAA	CTAT	AGAT							162114	162127	162122							+	+	-							MPN125	L
MPNs263	CTAT									164398									-									MPN126/MPN127	R
MPNs271	ATAC									197932									+									MPN149	M
MPNs274	ATAT	CTAT	AGAA							213983	213988	213991							+	+	-							MPN160	M
MPNs281	CTAT									243010									+									MPN200	M
MPNs287	CTAT									292596									+									MPN240	O
MPNs288	CTAT									296086									+									MPN243	K
MPNs292	GGAA	ATAA								305051	305059								+	-								MPN253	I
MPNs294	CTAT									309613									+									MPN258	G
MPNs296	AGAC	ATAA								313856	313864								+	-								MPN261	L
MPNs297	TGAT	CGAT	GTAA	GTAT						320766	320787	320774	320795						+	+	-	-						MPN269	R
MPNs298	CTAT									323155									-									MPN271/MPN272	M
MPNs300	CTAT									328364									-									MPN277/MPN278	J
MPNs302	CTAT									337972									+									MPN284	M
MPNs303	CTAT									340902									+									MPN285	V
MPNs310	CGAT	CTAT	ATAG							364709	364718	364717							+	+	-							MPN309	S
MPNs311	TGAT	ATAC								365523	365531								+	-								MPN309/MPN310	S
MPNs314	GGAT									378385									-									MPN318	E
MPNs316	ATAG	CTAT	AGAT							388904	388903	388912							+	-	-							MPN330	S
MPNs317	CTAT									395887									-									MPN334	R
MPNs323	CTAT	ATAG	CTAT							409391	409390	409401							+	-	-							MPN342	V
MPNs327	ATAT	CTAT	TGAA							443077	443081	443085							+	+	-							MPN144	M
MPNs329	CTAT									457915									+									MPN380	L
MPNs330	GTAT	TGAT	CTAT							473216	473224	473227							+	-	-							MPN395	F
MPNs341	CTAT	GGAG	ATAC							591713	591728	591736							+	+	-							MPN487	O

Continued on next page

Table S5 – Methylation in promoter sequences – continued from previous page																													
**ORF**	**Motif1**	**Motif2**	**Motif3**	**Motif4**	**Motif5**	**Motif6**	**Motif7**	**Motif8**	**Motif9**	**P1**	**P2**	**P3**	**P4**	**P5**	**P6**	**P7**	**P8**	**P9**	**Str1**	**Str2**	**Str3**	**Str4**	**Str5**	**Str6**	**Str7**	**Str8**	**Str9**	**Protein name (Overlapping gene for MPNs)**	**COG Category**
MPNs342	CTAT									596768									+									MPN490/MPN489	-
MPNs344	GTAT	AGAA								620513	620521								+	-								MPN508/MPN509	U
MPNs347	AGAA									627543									-									MPN513/MPN514	V
MPNs349	CTAT	CTAT								642755	642724								+	-								MPN520	J
MPNs354	CTAT									663390									-									MPN544/MPN545	S
MPNs360	ATAT									691911									-									MPN570	A
MPNs362	CTAT	CTAT	CTAT							711220	711233	711255							+	-	-							MPN588	M
MPNs364	AGAA									738937									-									MPN615	V
MPNs365	GTAG									742388									+									MPN618	L
MPNs367	TGAT	GTAA								759252	759260								+	-								MPN632	F
MPNs370	ATAA									778330									-									MPN652	G
MPNs373	AGAG	ATAA								789590	789598								+	-								MPN666	R
MPNs374	CTAT	ATAA	CTAT							791996	791998	792032							+	-	-							MPN669	J
MPNs380	GTAG									813555									+									MPN686	LD
MPNs381	CTAT									572									+									MPN688/MPN001	D
MPNs382	CTAT									134175									-									MPN103	S
MPNs383	CTAT	TGAA	CTAT	ATAT	ATCT	GGAT	CTAT	ATAG	AGAT	180395	180413	180422	180424	180426	180431	180435	180421	180432	+	+	+	+	+	+	+	-	-	MPN140	J
MPNt17	CTAT	CTAT								453354	453343								+	-								-	-
MPNt24	CTAT									555134									-									-	-
MPNt26	ATAA	AGAA								558597	558605								+	-								-	-
MPNt35	CTAT									645079									-									-	-
