# Supplementary material for: Comprehensive Methylome Characterization of Mycoplasma genitalium and Mycoplasma pneumoniae at Single-Base Resolution
Source: PLoS Genet. 2013 Jan 3;9(1):e1003191. doi: 10.1371/journal.pgen.1003191 (PMC3536716; doi:10.1371/journal.pgen.1003191)
Supplement: Table S6 — Functional enrichment of COG categories for promoter (a) and 5′ UTR regions (b). The functional enrichment was measured by comparing all the function of all the ORFs that have an associated promoter sequence or 5′UTR with those that have these regions methylated. Enrichment is measured by Fisher's test and significant enrichment is considered when the Pvalue<0.05 (marked with “*”). (PDF) [file pgen.1003191.s007.pdf]

Table S6a – COG enrichment for ORFs with assigned TSS

COG Category	Total Genes with Promoter	Genes Methylated in Promoter region	Odd ratio	(P-value)
A	11	8	1.3560588	0.337952212
C	12	4	0.605730992	0.873307704
D	6	4	1.233560811	0.491355483
E	7	7	1.8762773	0.184123405
F	7	6	1.599130388	0.286561276
G	16	10	1.160894875	0.433359699
H	4	4	1.860643562	0.29613475
I	12	5	0.761385794	0.774150416
J	45	21	0.840928447	0.772666182
K	11	3	0.494443032	0.921123563
L	19	13	1.281654672	0.312586286
M	57	35	1.164943555	0.300204486
N	26	6	0.405186307	0.989363724
O	19	9	0.866227611	0.703924001
P	8	4	0.919790845	0.660458326
R	6	6	1.871003323	0.213965107
S	24	15	1.166017928	0.387240443
T	2	1	0.921198924	0.728481606
U	10	5	0.919310222	0.656213483
V	15	6	0.728161659	0.809322491

Table S6b – COG enrichment for ORFs with assigned 5'UTR

COG Category	Total Genes with Promoter	Genes Methylated in Promoter region	Odd ratio	(P-value)
A	11	2	0.662952178	0.804855802
C	12	0	0	1
D	6	2	1.233445564	0.537087098
E	7	2	1.054287304	0.60575899
F	7	2	1.054287304	0.60575899
G	16	2	0.448654767	0.92639658
H	4	1	0.920775687	0.70089579
I	12	1	0.299637934	0.958173591
J	45	9	0.707032347	0.860967027
K	11	1	0.327914484	0.946379915
L	19	6	1.175858749	0.449968372
M	57	20	1.381069514	0.170785316
N	26	10	1.471129105	0.215261901
O	19	2	0.374106198	0.960458487
P	8	3	1.394824418	0.427579819
R	6	2	1.233445564	0.537087098
S	24	9	1.425582177	0.252481007
T	2	1	1.849590528	0.514285099
U	10	1	0.361839883	0.931310218
V*	15	10	2.641204604	0.022796312

*Enriched COG category
