# Supplementary material for: Comprehensive Methylome Characterization of Mycoplasma genitalium and Mycoplasma pneumoniae at Single-Base Resolution
Source: PLoS Genet. 2013 Jan 3;9(1):e1003191. doi: 10.1371/journal.pgen.1003191 (PMC3536716; doi:10.1371/journal.pgen.1003191)
Supplement: Table S7 — Methylation in 5′UTR regions. Start and End regions are the genome positions comprising the 5′UTR region. Column named “motifs” indicates the number of motifs identified. Str is the abbreviation for strand and indicates the gene orientation (“+” forward strand, “−” reverse strand). The rest of columns indicate the sequence of the motifs and the genome positions as well as, the strand for these motifs. The two last columns indicate the function and the COG category respectively. (PDF) [file pgen.1003191.s008.pdf]

Table S7 – Methylation in 5'UTR regions

ORF	Start	End	5'UTR size (bp)	Nr of motifs	Str	Motifs	P1	P2	P3	P4	P5	P6	P7	P8	P9	P10	P11	P12	P13	P14	Str1	Str2	Str3	Str4	Str5	Str6	Str7	Str8	Str9	Str10	Str11	Str12	Str13	Str14
MPN412a	497073	497073	0	2	+	CGAT CGAT	497073	497073													+	+												
MPN194	233600	233604	4	1	+	CTAT	233604														+													
MPN188	230585	230590	5	1	+	CTAT	230590														+													
MPN354	423493	423488	5	1	-	CTAT	423488														-													
MPNt24	555135	555142	7	1	+	AGAA	555137														+													
MPN208a	253325	253317	8	1	-	AGAT	253317														-													
MPN521	642750	642760	10	1	+	CTAT	642755														+													
MPN555	675658	675648	10	2	-	ATAT CGAT	675649	675657													+	-												
MPN083	103930	103941	11	1	+	CTAT	103934														-													
MPN592	716007	715996	11	1	-	CTAT	715996														-													
MPN652	777470	777481	11	2	+	AGAA ATAC	777471	777479													+	-												
MPN394	472927	472915	12	1	-	GTAA	472921														+													
MPN483	586760	586772	12	1	+	CTAT	586761														-													
MPN028	33046	33059	13	1	+	CTAT	33054														-													
MPN331	388910	388923	13	1	+	AGAT	388912														-													
MPN336	399405	399392	13	1	-	CTAT	399394														+													
MPN450	549604	549589	15	1	-	CTAT	549589														-													
MPN517	637070	637054	16	1	-	TGAT	637068														+													
MPN044	53069	53050	19	1	-	CTAT	53050														-													
MPN263	315855	315874	19	1	+	CTAT	315868														-													
MPN472	575170	575151	19	1	-	CTAT	575159														+													
MPN533	656172	656153	19	1	-	CTAT	656164														-													
MPN254	305047	305066	19	2	+	GGAA ATAA	305051	305059													+	-												
MPN411	496060	496040	20	1	-	CTAT	496053														-													
MPN666	789390	789410	20	2	+	GTAG TGAA	789398	789406													+	-												
MPN396	476487	476466	21	1	-	TGAC	476484														+													
MPN397	476485	476509	24	1	+	ATAA	476492														-													
MPN386	463855	463831	24	2	-	AGAA GTAA	463841	463849													+	-												
MPN606	727568	727544	24	2	-	ATAT AGAA	727545	727553													+	-												
MPN062	79008	79033	25	1	+	CTAT	79016														-													
MPN258	307970	307996	26	1	+	CTAT	307972														-													
MPN531	653975	653949	26	2	-	CTAT CTAT	653952	653975													+	-												
MPN246	297580	297610	30	2	+	TGAT TACG	297586	297591													-	-												
MPN129	167600	167632	32	1	+	CTAT	167632														+													
MPN426	514575	514542	33	1	-	CTAT	514566														-													
MPN266	318110	318144	34	4	+	CTAT AGAC CTAT ATAA	318110	318121	318118	318129											+	+	-	-										
MPN489	596335	596300	35	1	-	CTAT	596326														-													
MPN593	716500	716465	35	3	-	ATAT CTAT AGAC	716473	716465	716481												+	-	-											
MPN001	655	692	37	1	+	CTAT	681														+													
MPN035a	42050	42013	37	1	-	CTAT	42022														+													
MPN337	399440	399477	37	1	+	CTAT	399447														-													
MPN430	518890	518850	40	1	-	CTAT	518888														+													
MPN265	318128	318087	41	3	-	CTAT AGAC CTAT	318110	318121	318118												+	+	-											
MPN309	364188	364231	43	1	+	CTAT	364191														-													
MPN012	14310	14265	45	2	-	CTAT CTAT	14287	14294													+	-												
MPN484	588658	588613	45	4	-	ATAG CTAT TGAT CTAC	588619	588618	588627	588632											+	-	-	-										
MPN332	390282	390328	46	1	+	CTAT	390328														+													
MPN014	15890	15939	49	2	+	CTAT CTAT	15910	15929													+	-												
MPN541	661175	661126	49	2	-	CGAT ATAC	661142	661150													+	-												
MPN630	757832	757783	49	2	-	ATAT GGAC	757784	757792													+	-												
MPN675	799516	799467	49	4	-	ATAG CTAT TGAT CTAT	799473	799472	799481	799486											+	-	-	-										
MPN010	12342	12392	50	1	+	ATAC	12344														-													
MPN139	179670	179620	50	2	-	ATAA AGAA	179629	179637													+	-												
MPN439	532715	532662	53	1	-	CTAT	532670														-													
MPN043	51580	51634	54	1	+	CTAT	51581														-													
MPN071	86485	86428	57	1	-	GTAT	86428														-													
MPN342	407804	407861	57	5	+	ATAA ATAA CTAT TGAA TGAG	407820	407843	407856	407828	407851										+	+	+	-	-									
MPN013	15030	15088	58	1	+	CTAT	15068														+													
MPN275	325620	325680	60	2	+	CTAT CTAT	325645	325621													+	-												
MPN485	590042	589980	62	1	-	CTAT	589990														+													
MPN633	759515	759578	63	1	+	CTAT	759578														+													
MPN579	702258	702192	66	1	-	CTAT	702226														+													
MPN487	590500	590569	69	1	+	CTAT	590509														-													
MPN101a	131776	131848	72	2	+	CTAT CTAT	131784	131777													+	-												
MPN665	789400	789325	75	1	-	GTAG	789398														+													
MPN407	490223	490147	76	1	-	CTAT	490223														+													
MPN224	275422	275499	77	1	+	CTAT	275442														+													
MPN086	108780	108857	77	2	+	CTAT CTAT	108857	108802													+	-												
MPN676	799780	799858	78	1	+	CTAT	799842														+													
MPN371	444266	444187	79	3	-	CTGT CGAT ATAA	444191	444196	444204												+	+	-											
MPN154	205722	205801	79	4	+	ATAT GACG TGAC TTAA	205736	205743	205744	205749											+	-	-	-										

Continued on next page

Table S7 – Methylation in 5'UTR regions – continued from previous page

ORF	Start	End	5'UTR size (bp)	Nr of motifs	Str	Motifs	P1	P2	P3	P4	P5	P6	P7	P8	P9	P10	P11	P12	P13	P14	Str1	Str2	Str3	Str4	Str5	Str6	Str7	Str8	Str9	Str10	Str11	Str12	Str13	Str14
MPN164	217550	217630	80	2	+	AGAA ATAT	217621	217629													+	-												
MPN650	775425	775339	86	4	-	GTAA CTAT AGAT CTAT	775392	775363	775400	775416											+	-	-	-										
MPN035	41322	41409	87	1	+	CTAT	41401														+													
MPN538	659695	659782	87	1	+	CTAT	659763														+													
MPN115	150556	150643	87	2	+	CTAT GGAG	150614	150560													+	-												
MPN005	7225	7312	87	3	+	AGAC CTAT ATAG	7251	7260	7259												+	+	-											
MPN137	178230	178143	87	3	-	ATAC CTAT AGAA	178152	178185	178160												+	+	-											
MPN148	195450	195539	89	2	+	CTAT CTAT	195492	195482													+	-												
MPN651	776242	776337	95	1	+	CTAT	776284														-													
MPN520a	640400	640304	96	2	-	ATAA AGAC	640305	640313													+	-												
MPN488a	592578	592479	99	1	-	CTAT	592529														-													
MPN104a	134985	135088	103	2	+	TGAA ATAA	135017	135025													+	-												
MPN412	496525	496634	109	1	+	CTAT	496557														+													
MPN400	482110	481997	113	4	-	CTAT ATAG CTAT CTAT	482095	482106	482007	482105											+	+	-	-										
MPN198	238246	238364	118	2	+	GGAT GTAA	238257	238265													+	-												
MPN346	413310	413431	121	4	+	CTAT TGAA CTAT ATAT	413335	413342	413426	413350											+	+	+	-										
MPN623	749726	749850	124	2	+	CTAT CTAT	749837	749850													+	+												
MPN049	59480	59619	139	2	+	ATAC TGAA	59485	59493													+	-												
MPN016	18345	18205	140	1	-	CTAT	18295														+													
MPN089	111470	111610	140	1	+	ATAC	111541														+													
MPN506a	616120	615980	140	1	-	CTAT	616114														+													
MPN074	88485	88341	144	1	-	CTAT	88407														+													
MPN287	343620	343764	144	6	+	ATAA TTAC TGAT CTAT AGAC ATAG	343643	343745	343750	343759	343651	343758									+	+	+	+	-	-								
MPN347a	415550	415697	147	3	+	CTAT CTAT CTAT	415594	415560	415660												+	-	-											
MPN274	325807	325658	149	3	-	TGAG CTAT ATAG	325768	325777	325776												+	+	-											
MPN375	449345	449194	151	2	-	GGAA GTAA	449234	449242													+	-												
MPN335a	397770	397616	154	1	-	CTAT	397700														+													
MPN199a	241420	241575	155	1	+	CTAT	241489														+													
MPN482	586885	586713	172	3	-	CTAT ATAT CTAT	586718	586713	586761												+	-	-											
MPN128	166310	166483	173	1	+	CTAT	166406														+													
MPN374	448632	448459	173	1	-	CTAT	448536														-													
MPN655	780185	780008	177	1	+	CTAT	780035														+													
MPN029	33800	33979	179	4	+	CTAT AGAT CTAT ATAT	33800	33970	33967	33978											+	+	-	-										
MPN091	113646	113838	192	5	+	CTAT ATAA GGAT CTAT GGAA	113693	113763	113715	113766	113771										+	+	-	-	-									
MPN544	662582	662783	201	3	+	GTAA CTAT TGAA	662769	662653	662777												+	-	-											
MPN619	745407	745199	208	1	-	CTAT	745372														-													
MPN497	604801	604586	215	1	-	CTAT	604637														-													
MPN577	701322	701107	215	6	-	GTAA CTAT AGAG CTAT TGAA GTAG	701216	701242	701281	701158	701224	701289									+	+	+	-	-	-								
MPN379	456054	456272	218	1	+	CTAT	456054														-													
MPN047a	56979	56759	220	2	-	GGAG GTAC	56852	56860													+	-												
MPN151	198910	199139	229	4	+	ATAA TGAT AGAC GTAG	199018	199125	199026	199133											+	+	-	-										
MPN319	378420	378658	238	3	+	AGAG CTAT ATAA	378427	378658	378435												+	+	-											
MPN427	514480	514727	247	2	+	CTAT CTAT	514686	514566													+	-												
MPN276	327065	326811	254	1	-	CTAT	326950														-													
MPN344	411098	411383	285	1	+	CTAT	411234														-													
MPN365	435610	435924	314	5	+	TGAT CTAT CTAT ATAT CTAT	435778	435751	435760	435786	435820										+	-	-	-	-									
MPN098	126625	126947	322	3	+	CTTA TGAT GTAT	126808	126813	126821												+	+	-											
MPN042	48961	49292	331	1	+	CTAT	48995														+													
MPN596	718749	719144	395	2	-	ATAA CGAT	719064	719072													+	-												
MPN104	134175	134583	408	1	+	CTAT	134175														-													
MPN441	535050	535468	418	1	+	CTAT	535116														-													
MPN262	313950	314381	431	1	+	CTAT	314159														+													
MPN343	409438	409871	433	8	+	ATAA ATAA GATC TGAT AGAT AGAA TGAC GTAT	409501	409643	409717	409722	409749	409509	409651	409730							+	+	+	+	+	-	-	-						
MPN583	706680	706231	449	6	-	CTAT ATAG CTAT CTAT CTAT GGAT	706281	706569	706631	706523	706568	706577									+	+	+	-	-	-								
MPN285	340673	341207	534	3	+	CTAT GGAG GTAT	340902	340948	340956												+	+	-											
MPN154a	206820	206273	547	3	-	CGGT AGAT ATAT	206645	206650	206658												+	+	-											
MPN088	110230	110813	583	8	+	GTAA CTAT CTAT CTAT CTAT CTAT CGAT CTAT	110263	110585	110634	110646	110671	110765	110271	110377							+	+	+	+	+	+	-	-						
MPN111	144431	145021	590	11	+	TGAA AGAA ATAA ATAA GTAT CTAT CTAT GGAT CTAT CTAT CTAT	144490	144563	144795	144498	144571	144710	144764	144803	144887	144953	144989				+	+	+	-	-	-	-	-	-	-	-			
MPN432	521685	520972	713	2	-	ACAA CTAT	521323	521648													+	-												
MPN512	626858	626139	719	7	-	ATAT GTAA TGAC TGAC CAAG CTAT CTAT	626239	626547	626247	626555	626560	626688	626793								+	+	-	-	-	-	-							
MPN509	622592	621844	748	3	-	CTAT CTAT CTAT	621860	622355	621861												+	+	-											
MPN348	415140	416070	930	14	+	CTAT AGAA CTAT CGAT CTAT AGAA CTAT ATAG ATAA CTAT CTAT CTAT CTAT ATAT	415161	415243	415252	415353	415594	416061	415152	415251	415361	415368	415405	415560	415660	416069	+	+	+	+	+	+	-	-	-	-	-	-	-	-
