# Supplementary material for: Comprehensive Methylome Characterization of Mycoplasma genitalium and Mycoplasma pneumoniae at Single-Base Resolution
Source: PLoS Genet. 2013 Jan 3;9(1):e1003191. doi: 10.1371/journal.pgen.1003191 (PMC3536716; doi:10.1371/journal.pgen.1003191)
Supplement: Table S8 — Study of transcription in ORFs containing 5′-CTAT-3′ motifs that showed changes in methylation between 96 and 6 h. First column indicates methylated positions that show an increase of methylation in different phases of growth. Second column shows the strand where the motif is found. Third column indicates the IPD ratio between 6 h and 96 h. Fourth column, the Qmod values. Two next columns, show the genome location. Columns named as regions indicate if the region is intergenic (IG) coding or promoter. The columns named as “name” indicate the protein name of overlapping ORFs and the ones named as “category” the associated COG categories. The values of gene expression determined by Deep Sequencing Strand Specific (DSSS) at 6 and 96 hours are indicated in the columns named as ExpDSS 6 h and ExpDSS 96 hours respectively. Two last columns indicate changes in gene expression. Significant increase in expression is considered when the difference in expression between 96 and 6 hours higher than 1,5 exp increase). Significant decrease is considered when this difference is lower than −1,5 (Exp decrease) and no significant changes are considered when the value of the difference is between −1.5 and 1.5. (PDF) [file pgen.1003191.s009.pdf]

Table S8 – Study of transcription in ORFs containing 5’-CTAT-3’ motifs that showed changes in methylation between 96 and 6 h

Position	Str	IpdRatio 96hs vs 6hs	Qmod	ORFs	ORFs	Region	Region	name	name	category	category	Exp DSSS 6h	Exp DSSS 6h	Exp DSSS 96h	Exp DSSS 96h	change	change
109890	-	1.57	110.29	Intergenic		-		-		-		-		-		-	
116033	-	1.66	74.89	Intergenic		-		-		-		-		-		-	
119092	-	1.53	68.58	Mpnr01		Coding		-		-		17.89		17.72		UNCHANGED	
120911	-	1.42	81.6	Mpnr02		Coding		-		-		17.41		17.27		UNCHANGED	
165257	-	1.61	73.64	Intergenic		-		-		-		-		-		-	
315868	-	1.55	66.57	Intergenic		-		-		-		-		-		-	
323547	-	1.77	93.58	Intergenic		-		-		-		-		-		-	
364143	-	1.38	65.88	Intergenic		-		-		-		-		-		-	
495138	-	1.47	63.1	Intergenic		-		-		-		-		-		-	
717703	-	1.52	97.72	Intergenic		-		-		-		-		-		-	
450927	-	1.49	76.01	MPN376		Coding		-		A		14.36		14.38		UNCHANGED	
373908	+	1.68	65.25	MPN315		Coding		mraW		D		10.23		10.23		UNCHANGED	
90519	-	1.32	75.35	MPN076		Coding		uhpT		G		9.94		10.67		UNCHANGED	
56417	+	1.3	65.21	MPN047		Coding		PncB		H		4.36		5.38		UNCHANGED	
20637	+	1.47	101.63	MPN018		Coding		pmd1		I		6.48		7.25		UNCHANGED	
28642	+	1.65	108.22	MPN023		Coding		metS		J		5.28		5.53		UNCHANGED	
804220	+	1.45	84.56	MPN679	MPN680	Coding	Coding	ksgA	yidC	J	U	9.32	8.97	9.17	7.85	UNCHANGED	UNCHANGED
752332	+	1.59	81.11	MPN626	MPNs111	Coding	Promoter	-	-	K	-	3.26	2.19	6.08	5.12	INCREASES	INCREASES
454794	-	1.55	79.45	MPN378		Coding		dnaE		L		6.59		6.68		UNCHANGED	
47612	-	1.97	73.89	MPN039		Coding		-		M		7.14		7.84		UNCHANGED	
193237	-	1.43	89.73	MPN146		Coding		-		M		9.33		11.18		INCREASES	
243531	+	1.48	74.24	MPN200		Coding		-		M		6.54		7.99		UNCHANGED	
338057	-	1.61	74.31	MPN284		Coding		-		M		5.35		6.5		UNCHANGED	
626793	-	1.33	61.41	MPN512		5’UTR		-		M		10.31		9.37		UNCHANGED	
689027	+	1.45	68.51	MPN567		Coding		p2		M		10.13752776		9.83		UNCHANGED	
712185	+	1.49	60.72	MPN590		Coding		-		M		8.82		5.99		DECREASES	
130007	-	1.6	69.34	MPN100		Coding		-		N		7.92		9.36		UNCHANGED	
727612	+	1.47	78.69	MPN607		Coding		pmsR		OV		5.57		6.68		UNCHANGED	
727996	+	1.46	64.08	MPN607		Coding		pmsR		OV		5.5758665		6.683294904		UNCHANGED	
810078	-	1.7	69.52	MPN684		Coding		-		P		9.48		8.21		UNCHANGED	
104806	+	1.51	65.91	MPN083		Coding		-		S		9.08		10.35		UNCHANGED	
700173	-	1.4	60.03	MPN577		Coding		-		S		7.17		5.26		DECREASES	
735450	-	1.64	110.3	MPN612		Coding		-		S		7.02		6.83		UNCHANGED	
580030	+	1.41	78.52	MPN475		Coding		engA		T		7.7		7.58		UNCHANGED	
410575	+	1.36	76.88	MPN343		Coding		-		V		5.3		5.64		UNCHANGED	
