# Supplementary material for: Comprehensive Methylome Characterization of Mycoplasma genitalium and Mycoplasma pneumoniae at Single-Base Resolution
Source: PLoS Genet. 2013 Jan 3;9(1):e1003191. doi: 10.1371/journal.pgen.1003191 (PMC3536716; doi:10.1371/journal.pgen.1003191)
Supplement: Table S9 — a) Table of primers used in this study. b) Table of vectors used to clone and express different putative methyltransferases of M. pneumoniae in ER2796 strain. (PDF) [file pgen.1003191.s010.pdf]

Table S9a – Primers used in this study

Primer name	Sequence (5'-3')
5MPN107	ATACTGCAGTTAAGGTTAATCATATGACAAACCAAAACCTACAAGA
3MPN107	GATGGATCCCTAATTAATTTGAAATTGA
5-CTAT-Biot	CTATATCCTATCGACTATCTTCTATCACTATA
3-CTAT	TATAGTGATAGAAGATAGTCGATAGGATATAG
5-GA(N7)TAY-Biot	TCTTGATGGCGAATACGTTACCTGAACTACAGATGGTATATATGCAG
3-GA(N7)TAY	CTGCATATACCATCTGTAGTTCAGGTAACGTATTCGCCATCAAGA

Table S9b – Vectors used to clone and express different putative methyltransferases

Plasmid Name	Description
pRRS	Clonning vector (New England Biolabs; Genbank accession number JN569339)
pRSS107	Plasmid derived from pRSS where mpn107 gene was clonned PstI-BamHI
pRSS198	Plasmid derived from pRSS where mpn198 gene was clonned PstI-BamHI
pRSS108	Plasmid derived from pRSS where mpn108 gene was clonned PstI-BamHI
